# A DFT calculation-inspired Rh(i)-catalyzed reaction *via* suppression of α-H shift in α-alkyldiazoacetates†
†Electronic supplementary information (ESI) available: DFT calculation details, optimized structures, experimental data and biological activity tests. CCDC 984118. For ESI and crystallographic data in CIF or other electronic format see DOI: 10.1039/c7sc00257b



**DOI:** 10.1039/c7sc00257b

**Published:** 2017-03-22

**Authors:** Shunying Liu, Jun Jiang, Jianghui Chen, Qinghua Wei, Wenfeng Yao, Fei Xia, Wenhao Hu

**Affiliations:** a Shanghai Engineering Research Center of Molecular Therapeutics and New Drug Development , East China Normal University , Shanghai , 200062 , China . Email: fxia@chem.ecnu.edu.cn ; Email: whu@chem.ecnu.edu.cn; b School of Chemistry and Chemical Engineering , Guangxi University , Nanning , 530004 , China; c NYU-ECNU Center for Computational Chemistry at NYU Shanghai , Shanghai , 200062 , China

## Abstract

Metal-associated carbenes from diazo compounds promote many useful chemistry transformations in modern organic chemistry. However, compared to α-aryldiazoacetate-derived carbenes (ArDCs), the synthetic application of α-alkyldiazoacetate-derived carbenes (AlDCs) is greatly limited due to intramolecular α-H transfer (elimination) that results in alkenes as the main by-products. An intriguing α-alkyldiazoacetate-involved three-component reaction has been developed following DFT calculation inspiration to provide β-hydroxyl α-alkyl-α-amino acid derivatives in good yields. The intramolecular α-H shift of an α-alkyldiazoacetate-derived carbene was successfully suppressed by the association of a Rh(i) complex to form the corresponding active ammonium ylide, which was trapped before the fast 1,2-H transfer process. A Rh(i)-chiral diene complex was identified as an effective catalyst to give an asymmetric version of the reaction with good enantioselectivity. This reaction provides insight into extending the efficient transformation of α-alkyldiazoacetate-derived carbenes and their synthetic application.

## Abstract 2

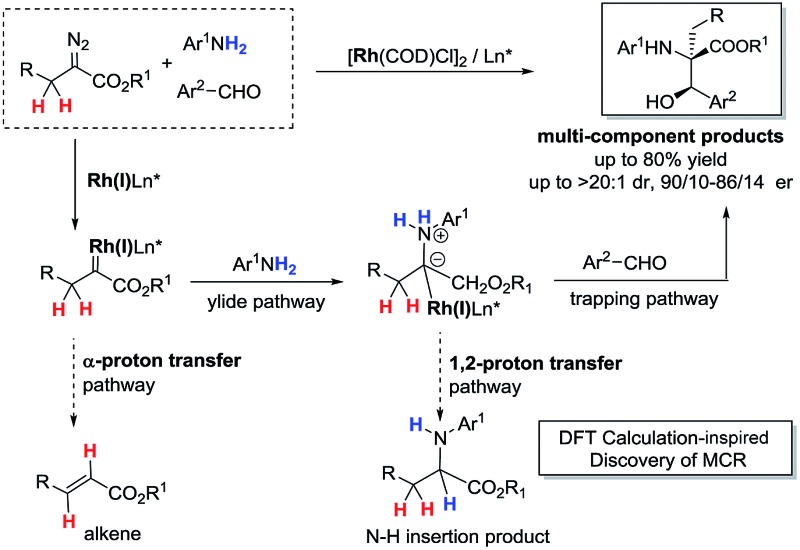
An α-alkyldiazoacetate-involved three-component reaction has been developed *via* the suppression of intramolecular α-H shift and 1,2-proton transfer by Rh(i) complexes.

## Introduction

**Scheme 1:**
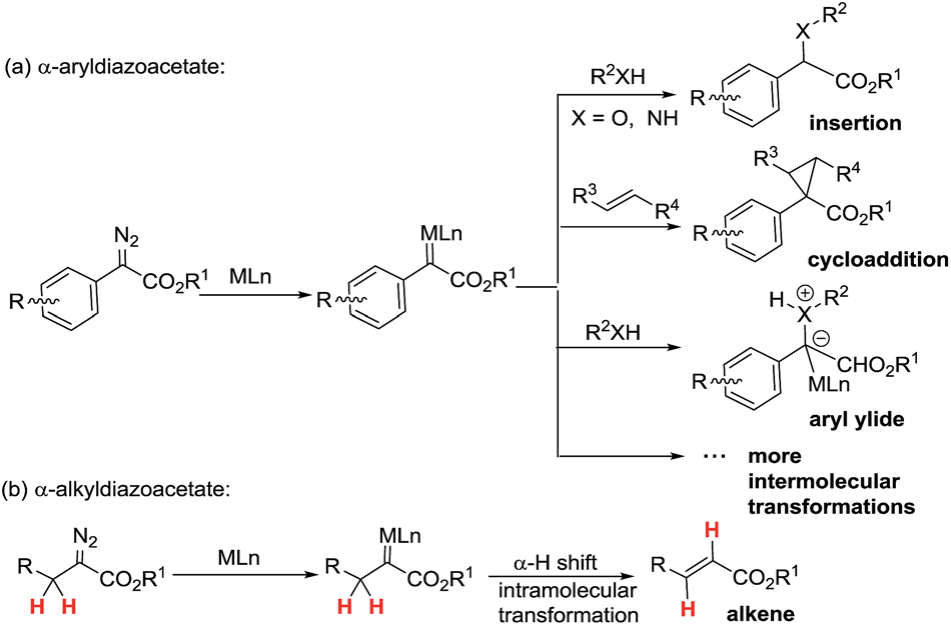
The main transformations of ArDCs (a) *vs.* AlDCs (b).

Metal-associated carbenes from diazo compounds play important roles in modern organic chemistry as active intermediates in useful chemistry transformations including X–H (X = C, O, N, *etc.*) insertions,^1^ cyclopropanations,^2^ ylides,^3^ 1,2-migration,^4^ *etc.* ^5^ Among these transformations, efficiently developed transformations of metal-associated diazo carbenes are mainly established from α-aryldiazoacetate-derived carbenes (ArDCs) without α-protons (Scheme 1a). Compared to ArDCs, the synthetic application of α-alkyldiazoacetate-derived carbenes (AlDCs) is greatly limited. Their limitation in synthetic application is mainly attributed to the fact that AlDCs can readily undergo intramolecular α-H transfer (elimination), which results in alkenes as the main by-products (Scheme 1b). Even though there are several elegant examples of AlDCs for X–H insertion that were developed by Zhou,^6^ Feng^7^ and others,^8^ the development of AlDC chemistry is still greatly in demand.

Very recently, a Rh(i)-associated three-component reaction of α-phenyldiazoacetate, aniline and β-nitroacrylates was successfully developed by our group.^9^ For this reaction, we found that Rh_2_(OAc)_4_ only afforded a trace amount of the desired product even though the diazo compounds were completely decomposed. This result indicated that there was an obvious difference in the activation energies of Rh(i)- and Rh(ii)-associated carbenes, and this prompted us to perform density functional theory (DFT) calculations. The investigations were conducted using the M06/Lanl2dz+6-31G* method^10,11^ in Gaussian 09 software,^12^ which is commonly used for describing metal carbene reactions.^11,13^ The frequency analyses were performed on optimized structures in the gas phase to verify whether they were transition states or stable structures. The solvent effect of CH_2_Cl_2_ was evaluated using the integral equation formalism model (IEFPCM).^14^ More computational details are provided in the section on DFT calculations in the ESI.†

**Scheme 2:**
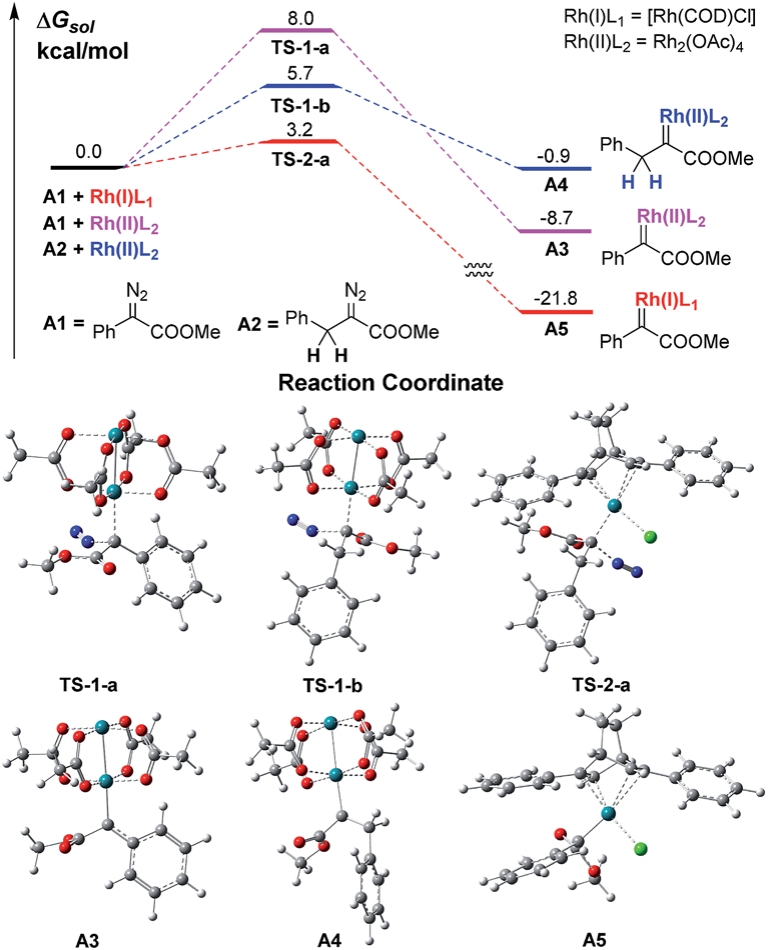
The free energy profiles of Rh(i)- and Rh(ii)-associated PhDCs.

We firstly conducted a conformation search on the generated Rh carbenes **A3**, **A4** and **A5**, and their most stable conformers are shown in Scheme 2 and in the ESI.† It was found that the energy of Rh(ii)-associated PhDC **A3** was much higher than that of Rh(i)-associated PhDC **A5** by 13.1 kcal mol^–1^. The calculated barriers of nitrogen extrusion *via* **TS-1-a**, **TS-1-b** and **TS-2-a** are 8.0, 5.7 and 3.2 kcal mol^–1^, respectively, relative to their reactants, and are consistent with the values reported in the literatures^13a,b^ for other reactions. These results confirm that the catalyst’s metal centre has an obvious effect on the activation energies of the reactions.

A further DFT calculation revealed that when using Rh_2_(OAc)_4_ as the decomposition catalyst, the energy of a benzyldiazoacetate-derived carbene (BnDC), one of the most easily-prepared AlDCs, is actually much higher than that of a PhDC by 7.8 kcal mol^–1^, denoted as **A4** and **A3** in Scheme 2, respectively, which indicates that a BnDC is a more active intermediate than a PhDC. These results highly inspired us to hypothesize that Rh(i) complexes could possibly lead to lower energy metal-associated AlDCs that are relatively stable, to provide useful transformations before α-H transfer.

## Results and discussion

The *in situ* generation of active ylide intermediates is one of the most important transformations of diazo carbenes, and can lead to further new transformations.^15^ We have been interested in discovering multicomponent reactions (MCRs) *via* a strategy of trapping Rh(ii)-associated active ylides with appropriate electrophiles before rapid 1,2-proton transfer^16^ to construct α-aryl-α-amino acids.^15b,17^ For instance, trapping oxonium ylides with imines provided rapid access to β-hydroxyl α-aryl-α-amino acids.^17^ MCRs are among the most powerful transformations for constructing complex molecules from simple starting materials,^18^ thus we decided to develop a Rh(i)-catalyzed MCR by trapping alkyl ylides *via* AlDCs to validate our hypothesis.

**Scheme 3:**
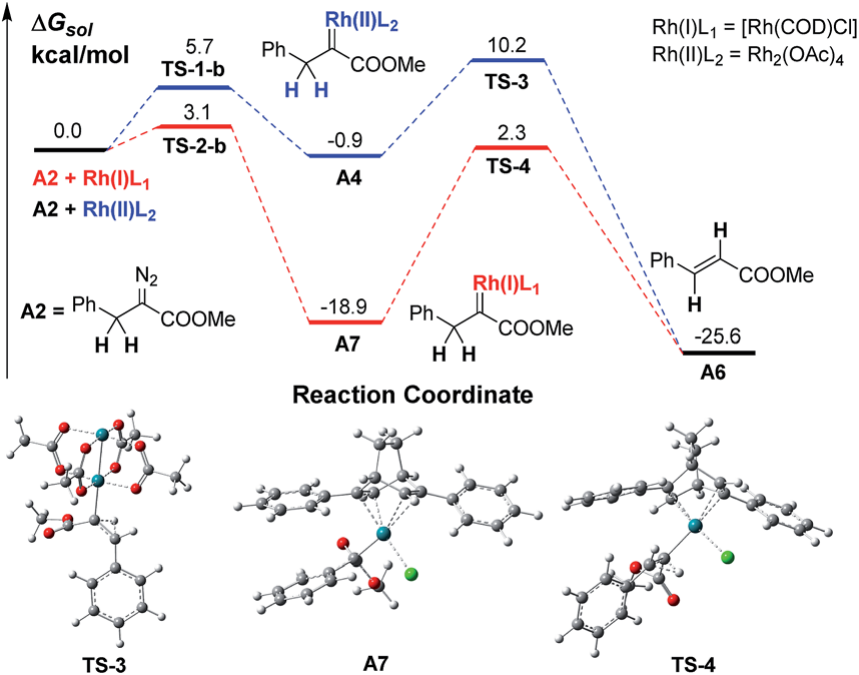
The free energy profile of the transformation of Rh(i)- and Rh(ii)-associated α-H transfer in BnDCs.

A preliminary investigation using DFT calculations of a Rh(i)-associated BnDC and its transformation into an α-H transfer product *vs.* a Rh(ii)-associated BnDC was conducted (Scheme 3). As expected, the energy of the Rh(i)-associated BnDC is much lower than that of the Rh(ii)-associated BnDC by 18.0 kcal mol^–1^. This suggests that the Rh(i)-associated BnDC possibly favors an ylide generation pathway over an α-H transfer pathway. The energy barriers of the transition states from the Rh(ii)- and Rh(i)-associated BnDCs (**TS-3** and **TS-4**, respectively) to their corresponding α-H transfer products were further investigated. The energy barrier of **TS-3** is 11.1 kcal mol^–1^, illustrating a very fast α-H transfer process. The energy barrier of **TS-4** is 21.2 kcal mol^–1^, which suggests the possibility that a Rh(i)-associated BnDC can be transformed into an ylide before α-H transfer. Thus, it is promising to develop MCR involving α-alkyldiazo compounds. To promote the desired MCR by trapping the alkyl ylide with a third component, both of the two very fast intramolecular processes, the α-H transfer in the metal carbene and the 1, 2-H transfer in the resulting ylide, should be overcome.

To confirm the theoretical results from the DFT investigations, our investigation of transformations began with the reaction of 2-diazo-3-phenylpropanoate (**1a**), 2-methoxyaniline (**2a**) and 4-nitrobenzaldehyde (**3a**) with 10 mol% catalyst (Table 1). The common catalyst for diazo decomposition, Rh_2_(OAc)_4_, was initially used to catalyze the reaction. A desired product **4a** was obtained in a moderate 50% isolated yield but with poor diastereoselectivity (1.6 : 1 dr, entry 1). Rh_2_(TFA)_4_ and [RuCl_2_(*p*-cymene)]_2_ were also employed but no desired products were observed (entries 2–3). Significant amounts of imines from the condensation of **2a** and **3a** were observed as the main side products. [{PdCl(η^3^-C_3_H_5_)}_2_] catalyzed this reaction in only 10% yield and with no diastereoselectivity (entry 4). Then, [Rh(COD)Cl]_2_ was used and, as expected, was found to be a much more effective catalyst to give product **4a** in 75% yield and with a 7 : 1 dr favoring the *threo* isomer (entry 5). Reducing the amount of [Rh(COD)Cl]_2_ seriously decreased the yield (entries 6–7). Dichloromethane (DCM) was identified as a superior solvent over dichloroethane (DCE) and toluene to improve the diastereoselectivity and retain a good yield, even with a smaller amount of catalyst (65% yield, 12 : 1 dr, entry 9 *vs.* 5 and 6). A higher temperature was better for obtaining a higher yield but unfavorable to the diastereoselectivity (entry 10 *vs.* 11).

**Table 1:** Catalyst screening and optimization of the reaction conditions^*a*^

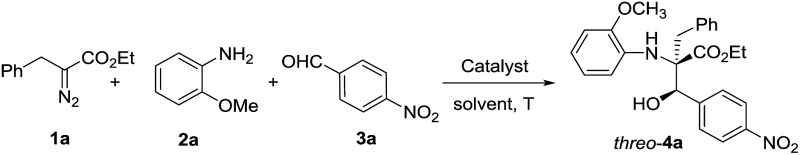					
Entry	Catalyst	Solvent	*T* (°C)	Yield^*b*^ (%)	dr^*c*^
1	Rh_2_(OAc)_4_	Toluene	rt	50	1.6 : 1
2	Rh_2_(TFA)_4_	Toluene	rt	N.R.^*d*^	—
3	[RuCl_2_(*p*-cymene)]_2_	Toluene	rt	N.R.	—
4	[{PdCl(η^3^-C_3_H_5_)}_2_]	Toluene	rt	10	1 : 1
5	[Rh(COD)Cl]_2_	Toluene	rt	75	7 : 1
6^*e*^	[Rh(COD)Cl]_2_	Toluene	rt	55	7 : 1
7^*f*^	[Rh(COD)Cl]_2_	Toluene	rt	34	4 : 1
8^*e*^	[Rh(COD)Cl]_2_	DCE	rt	57	10 : 1
9^*e*^	[Rh(COD)Cl]_2_	DCM	rt	65	12 : 1
10^*e*^	[Rh(COD)Cl]_2_	DCM	0	58	13 : 1
11	[Rh(COD)Cl]_2_	DCM	40	80	2 : 1

^*a*^Unless otherwise noted, all of the reactions were carried out at a 0.1 mmol scale, 10 mol% catalyst and **1a** : **2a** : **3a** = 1.0 : 1.2 : 2.0.

^*b*^Isolated yields.

^*c*^Detected using ^1^H NMR.

^*d*^N.R. = no reaction.

^*e*^Carried out using 4 mol% [Rh(COD)Cl]_2_.

^*f*^Carried out using 2 mol% [Rh(COD)Cl]_2_.

The efficiency of [Rh(COD)Cl]_2_ in promoting the alkyldiazoacetate-involved MCR compared to that of Rh_2_(OAc)_4_ was further illustrated using ethyl 2-diazopropanoate and ethyl 2-diazopentanoate, both of which gave a higher yield and much better diastereoselectivity (Table 2).

**Table 2:** Alkyldiazoacetate-involved MCR promoted by Rh_2_(OAc)_4_ and [Rh(COD)Cl]_2_ ^*a*^

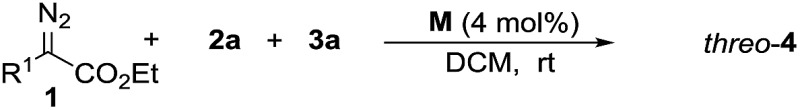			
Entry	**1**	Yield (%)/Dr/**4**	
		Rh_2_(OAc)_4_	[Rh(COD)Cl]_2_
1	R^1^ = Me	70/1 : 1/**4b**	80/4 : 1/**4b**
2	R^1^ = *n*-Pr	30/1 : 1/**4c**	66/10 : 1/**4c**

^*a*^The reactions were carried out in the same way as those in Table 1.

Under the optimized reaction conditions, a wide range of aldehydes, amines and diazo compounds was evaluated (Table 3). In most cases, a more electron-withdrawing aromatic aldehyde gave a higher product yield (entries 1–5), while a low yield of the three-component product was obtained with benzaldehyde and only a trace amount of product was obtained with electron-rich anisaldehyde (entries 6 and 7). These results are possibly due to the relatively weak electrophilic features of the electron-rich compounds to the active ammonium ylide. This process was also tolerant to other aromatic amines with different substituents on the aromatic rings to give a moderate yield and good diastereoselectivity up to >20 : 1 dr (entries 8–14). The diazo compounds were further extended to include *tert*-butyl-2-diazoacetate, giving the corresponding product in 41% yield and 2 : 1 dr (entry 15). The relative configuration of (2R*, 3R*)-*threo*-**4o** was established using X-ray single crystal analysis (see the figure in Table 3 and the ESI†).

**Table 3:** *threo*-Selective three-component reactions catalyzed by [Rh(COD)Cl]_2_ ^*a*^

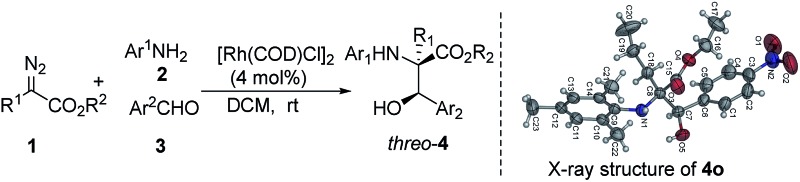					
Entry	R^1^/R^2^	Ar^1^	Ar^2^	Yield (%)	dr
1	Bn/Et	*o*-MeOC_6_H_4_	*p*-NO_2_C_6_H_4_	**4a**, 65	12 : 1
2	Bn/Et	*o*-MeOC_6_H_4_	*o*-NO_2_C_6_H_4_	**4d**, 50	2 : 1
3	Bn/Et	*o*-MeOC_6_H_4_	*p*-CNC_6_H_4_	**4e**, 30	7 : 1
4	Bn/Et	*o*-MeOC_6_H_4_	*p*-BrC_6_H_4_	**4f**, 50	6 : 1
5	Bn/Et	*o*-MeOC_6_H_4_	*m*-BrC_6_H_4_	**4g**, 56	3 : 1
6	Bn/Et	*o*-MeOC_6_H_4_	C_6_H_5_	**4h**, 18	4 : 1
7	Bn/Et	*o*-MeOC_6_H_4_	*p*-MeOC_6_H_4_	Trace	—
8	Bn/Et	2,4,6-CH_3_C_6_H_2_	*p*-NO_2_C_6_H_4_	**4i**, 31	3 : 1
9	Bn/Et	C_6_H_5_	*p*-NO_2_C_6_H_4_	**4j**, 55	>20 : 1
10	Bn/Et	*p*-EtOC_6_H_4_	*p*-NO_2_C_6_H_4_	**4k**, 45	2 : 1
11	Bn/Et	3,4,5-MeOC_6_H_2_	*p*-NO_2_C_6_H_4_	**4l**, 44	4 : 1
12	Bn/Et	*p*-MeOC_6_H_4_	*p*-NO_2_C_6_H_4_	**4m**, 45	10 : 1
13	Bn/Et	*o*-EtOC_6_H_4_	*p*-NO_2_C_6_H_4_	**4n**, 60	10 : 1
14	*n*-Pr/Et	2,4,6-CH_3_C_6_H_2_	*p*-NO_2_C_6_H4	**4o**, 52	>20 : 1
15	H/Bu^*t*^	*o*-MeOC_6_H_4_	*p*-NO_2_C_6_H4	**4p**, 41	2 : 1

^*a*^The reactions were carried out in the same way as those in Table 1.

Our next efforts were focused on achieving enantioselective control of the reaction. Chiral Rh_2_(*S*-NTTL)_4_ and Rh_2_(*S*-DOSP)_4_, which are widely employed for the decomposition of diazo compounds, gave products that were almost completely racemic in very low yields in this case (3% ee and 4% ee, respectively). Using a synergistic catalytic system comprising Rh_2_(OAc)_4_ and chiral Zr/binol/molecular sieves^17*b*^ or phosphoric acids,^17*c*^ three-component products of skeletal β-hydroxyl α-alkyl-α-amino acid were obtained in less than 10% ee. Inspired by the success of using chiral diene ligands in Rh(i) catalysis as reported by Hayashi and Carreira,^19^ the Rh(i)/diene complexes were used to catalyze the reaction. Excitingly, the enantioselective three-component reaction was effectively accomplished to afford chiral β-hydroxyl α-alkyl-α-amino acid derivatives with moderate to good diastereoselectivity and enantiostereoselectivity (3 : 1 to 4 : 1 dr, 86 : 14 to 91 : 9 er, Table 4). β-Hydroxyl-α-amino acid derivatives including the β-hydroxyl-α-alkyl-α-amino acid moiety have been widely found in peptides (such as droxidopa, cyclosporin, vancomycin, *etc.*), enzyme inhibitors and other physiologically active compounds.^20^ Numerous efforts have been made to develop synthetic approaches for β-hydroxyl-α-amino acid core structures.^21^ Herein, the established method provides a facile construction strategy for these compounds from simple starting materials under mild conditions.

**Table 4:** Rh(i)-catalyzed asymmetric three-component reactions of α-alkyldiazoacetate^*a*^

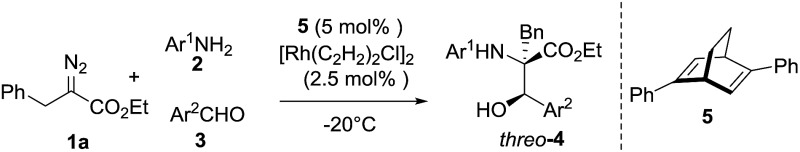					
Entry	Ar^1^	Ar^2^	Yield (%)	dr	er^*b*^
1	*o*-MeOC_6_H_4_	*p*-NO_2_C_6_H_4_	**4a**, 51	4 : 1	91 : 9
2	*o*-MeOC_6_H_4_	*o*-NO_2_C_6_H_4_	**4d**, 39	3 : 1	88 : 12
3	*o*-MeOC_6_H_4_	*p*-BrC_6_H_4_	**4f**, 42	4 : 1	86 : 14
4	3,4,5-MeOC_6_H_2_	*p*-NO_2_C_6_H_4_	**4l**, 41	4 : 1	86 : 14

^*a*^All of the reactions were carried out at a 0.1 mmol scale, and **1** : **2** : **3** = 2.0 : 1.0 : 1.2.

^*b*^Determined using HPLC.

**Scheme 4:**
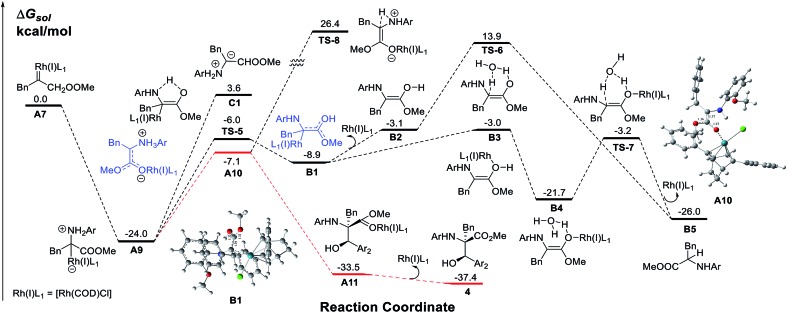
The calculated free energy profiles for the Rh(i) carbene in the N–H process and the multi-component process.

To obtain insight into the reaction mechanism, a detailed investigation using DFT calculations on the N–H insertion process *via* 1, 2-H transfer and the MCR process was conducted (Scheme 4). The results show that the nucleophilic attack of amines on the Rh(i) carbene is very facile with no energy barrier while the free ylide **C1** pathway has an energy barrier as high as 27.6 kcal mol^–1^. A Rh(i)-associative enol intermediate **B1** was formed *via* the transition state **TS-5**. The water-associated transition states **TS-6** (metal-dissociative pathway) and **TS-7** (metal-associative pathway)^22^ were well placed to be involved in the N–H insertion process. These results are consistent with those from previous theoretical studies on X–H bond insertion mechanisms performed by Yu and co-workers.^23^

Then, we examined the trapping of ammonium ylides by aldehydes. Interestingly, for the trapping pathway, the precursor for the attack of **A9** by aldehydes is preferred to be the enolate intermediate **A10**, where the Rh(i)-ligand is attached to the oxygen atom of the carbonyl group of the carbene, rather than the enol intermediate **B1**. The DFT calculations (Fig. S1 in the ESI†) indicate that the MCR process has no energy barrier, with the release of a considerable amount of energy of 33.5 kcal mol^–1^ due to the C–C bond formation, concomitant with a spontaneous proton transfer from the amide group to the carbonyl oxygen atom on the aldehyde. The release of the Rh(i)-ligand from **A11** yields the stable MCR product **4**, with an exothermicity of 37.4 kcal mol^–1^. A comparison of the calculated pathways for N–H bond insertion and MCR clearly reveals that the associative pathway of N–H bond insertion is still less favorable in terms of kinetics and thermodynamics than that of MCR. These calculated results are in good agreement with our observations in experiments.

**Scheme 5:**
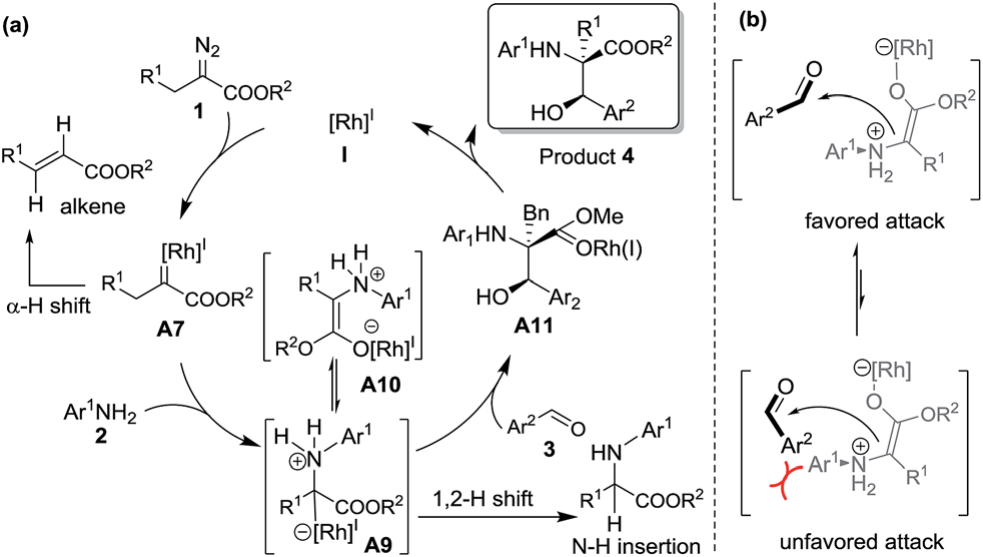
Plausible reaction mechanism and transition states.

The proposed reaction mechanism is shown in Scheme 5. The reaction proceeds through Rh(i)-associated ammonium ylide intermediates **A9**/**A10**, which are generated from Rh(i)-associated carbene **A7** and **2**. The intermediates **A9**/**A10** are trapped by electrophilic aldehyde **3** *via* a favored attack model, leading to the addition intermediate **A11**. The dissociation of the Rh(i)-ligand from **A11** with simultaneous 1, 2-proton transfer gives rise to the desired product **4**.

We then investigated the protein tyrosine phosphatase 1B (PTP1B) inhibitory activity of the products from this novel Rh(i)-catalyzed reaction (**4a–d**), with the aim of searching for new ways to treat type 2 diabetes mellitus (T2DM) and obesity.^24^ **4a** and **4c** showed significant inhibitory activity against PTP1B, and the IC_50_ values were 10.04 and 5.73 μg mL^–1^, respectively.

## Conclusions

In summary, we have discovered the first DFT-calculation inspired Rh(i)-catalyzed three-component reaction of α-alkyldiazoacetates *via* trapping of ammonium ylides before two rapid intramolecular processes: α-H transfer in alkyl carbenes and 1,2-H transfer in the resulting alkyl ylide. The α-H transfer in the α-alkyldiazoacetate carbene precursor was efficiently suspended by the association of the Rh(i) complex. An attempt to develop an asymmetric version of the Rh(i)/diene system was also successful with good enantioselectivity, and the biological activity of the products was primarily validated. The developed method provides an insight into extending the efficient transformation of α-alkyldiazoacetate-derived carbenes and affords β-hydroxyl α-alkyl-α-amino acids in moderate to good yields with diastereoselectivity.
