# Supplementary material for: A DFT calculation-inspired Rh(i)-catalyzed reaction via suppression of α-H shift in α-alkyldiazoacetates
Source: Chem Sci. 2017 Mar 22;8(6):4312–7. doi: 10.1039/c7sc00257b (PMC5635810; doi:10.1039/c7sc00257b)
Supplement: Supplementary file 1 [file SC-008-C7SC00257B-s001.pdf]

Electronic Supplementary Material (ESI) for Chemical Science.
This journal is © The Royal Society of Chemistry 2017

A DFT Calculation-inspiring Rh(I)-catalyzed Reaction via Suppression of α-H Shift in α-Alkyldiazoacetates

Shunying Liu, [a‡] Jun Jiang, [b‡] Jianghui Chen, [a] Qinghua Wei, [a] Wenfeng Yao, [a] Fei Xia[a,c*], and Wenhao Hu[a*]

[a] Shanghai Engineering Research Center of Molecular Therapeutics and New Drug Development, East China Normal University, Shanghai, 200062, P. R. China

[b] School of Chemistry and Chemical Engineering, Guangxi University, Nanning, 530004, P. R. China

[c] NYU-ECNU Center for Computational Chemistry at NYU Shanghai, Shanghai 200062, China

Corresponding Author

*E-mail: fxia@chem.ecnu.edu.cn; whu@chem.ecnu.edu.cn

Contents:

General remarks and materials……………………………………………………….…S-2

General procedure for the three-component reaction………………………….…….…S-2

^{1}H NMR, ^{13}C NMR and HPLC data for new compound……………………………..S-3

X-ray diffraction parameters and data of *threo*-4c………………………………………...S-9

^{1}H NMR and ^{13}C NMR spectra for new compounds………………………….……………...S-11

HPLC spectra for chiral compounds…………………….……………………………….……..S-26

DFT calculation details………….…….…………………..……………………………….....S-32

Figure S1…………………………………………………………………………………….....S-33

Optimized structures and coordinates in Schemes 2, 3 and 4……………………………..S-34

Biological activity test…………………………………….…..……………………………....S-79

General Remarks and Materials:

All reactions and manipulations were carried out under an argon atmosphere, in a flame-dried or oven-dried flask containing magnetic stir bar. All ^{1}H NMR, and ^{13}C NMR spectra were recorded using a Brucker 400 MHz or 500 MHz spectrometer in $CDCl_3$. Tetramethylsilane (TMS) served as an internal standard (δ = 0) for ^{1}H NMR, and $CDCl_3$ was used as internal standard (δ 77.0) for ^{13}C NMR. Chemical shifts are reported in parts per million as follows: chemical shift, multiplicity (s =singlet, d = doublet, t = triplet, q = quartet, m = multiplet, br = broad). HRMS (ESI) Mass spectra were recorded on IonSpec FT-ICR mass spectrometer. HPLC analysis was performed on Dalian Elite (UV230+ UV/Vis Detector and P230P High Pressure Pump). Chiralpak AD-H, IA was purchased from Daicel Chemical Industries, LTD. The racemic standards used in HPLC studies were prepared according to the general procedure by using racemic BINOL derived phosphoric acid catalysts. Dichloromethane (DCM), 1, 2-dichloroethane (DCE) and toluene was distilled over calcium hydride. Hayashi chiral diene ligand was prepared according to the literature procedure.[1]

General Procedure for catalyst screening and optimization of reaction conditions (Table 1):
A mixture of metal catalyst (0.01 mmol), aldehyde **3a** (0.12 mmol) in 1.0 mL of solvent under an argon atmosphere was stirred under the indicated temperature in table 1. Diazo compound **1a** (0.20 mmol) and amine **2a** (0.1 mmol) in 1.0 mL of solvent was then added over 1 h via a syringe pump. After completion of the addition, the reaction mixture was filtrated and evaporated *in vacuo* to give the crude product. The crude product was purified by flash chromatography on silica gel (eluent: EtOAc/light petroleum ether = 1:20 ~ 1:5) to give the pure product.

General Procedure for the *threo*-Selective three-component Aldol-type reactions catalyzed by $[Rh(COD)Cl]_2$ (Table 2):
A mixture of $[Rh(COD)Cl]_2$ (0.004 mmol), aldehyde **3** (0.10 mmol) in 1.0 mL of dichloromethane under an argon atmosphere was stirred under rt. Diazo compound **1** (0.20 mmol) and amine **2** (0.12 mmol) in 1.0 mL of dichloromethane was then added over 1 h via a syringe pump. After completion of the addition, the reaction mixture was filtrated and evaporated *in vacuo* to give the crude product. The crude product was purified by flash chromatography on silica gel (eluent: EtOAc/light petroleum ether = 1:20 ~ 1:5) to give the pure product.

General Procedure for Rhodium(I)-catalyzed asymmetric three-component Aldol-type reactions (Table 3):
A mixture of $[Rh(C_2H_2)_2Cl]_2$ (0.0025 mmol) and chiral diene complexin 1.0 mL of solvent of toluene and cyclohexane (1:1) was stirred under room temperature for 1 hour. Aldehyde **3** (0.12 mmol) was added. Diazo compound **1** (0.20 mmol) and amine **2** (0.10 mmol) in 1.0 mL of dichloromethane was then added in -20 ℃ over 1 h via a syringe pump. After completion of the addition, the reaction mixture was filtrated and evaporated *in vacuo* to give the crude product. The crude product was purified by flash chromatography on silica gel (eluent: EtOAc/light petroleum ether = 1:20 ~ 1:5) to give the pure product.

References: [1] S. Abele; R. Inauen; D. Spielvogel; C. Moessner, *J. Org. Chem.* **2012**, *77*, 4765-4773.

^{1}H NMR, ^{13}C NMR and HPLC data for new compounds:

Ethyl (2*R*, 3*R*)-2-benzyl-3-hydroxy-2-((2-methoxyphenyl) amino)-3-(4-nitrophenyl) propanoate (4a)

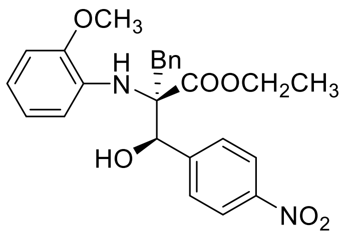

^{1}H NMR (400 MHz, $CDCl_3$, 25 ℃, TMS) *δ* 8.16 (d, *J* = 7.0 Hz, 2H), 7.54 (d, *J* = 7.0 Hz, 2H), 7.21 (m, *J* = 2.8 Hz, 3H), 7.00 (m, 2H), 6.89-6.86 (m, 2H), 6.85-6.78 (m, 2H), 5.69 (s, 1H), 5.14(s, 1H), 4.57 (s, 1H), 4.02-3.96 (m, 1H), 3.85 (d, *J* = 6.0 Hz, 1H), 3.81 (s, 3H), 3.49 (d, *J* = 11.3 Hz, 1H), 3.00 (d, *J* = 11.3 Hz, 1H), 0.87 (t, *J* = 5.7Hz, 3H). **^{13}C NMR** (100 MHz, $CDCl_3$, 25 ℃, TMS) δ 174.17, 147.70, 147.41, 146.34, 135.10, 134.54, 129.95, 128.69, 128.05, 126.96, 122.48, 120.85, 112.98, 110.42, 74.14, 68.04, 61.69, 55.70, 37.61, 13.34. **HRMS** (ESI) Calcd for $C_{25}H_{26}N_2O_6$ $(M+Na)^+$ 473.1477, Found: 473.1783. **HPLC** (Chiral OD-H, λ = 254 nm, hexane/2-propanol = 5:1, Flow rate = 0.8 mL/min), t_R= 11.58 min, 12.70 min.

Ethyl (2*R**, 3*R**) -3-hydroxy-2-((2-methoxyphenyl)amino)-2-methyl-3 -(4-nitrophenyl) propanoate (4b)

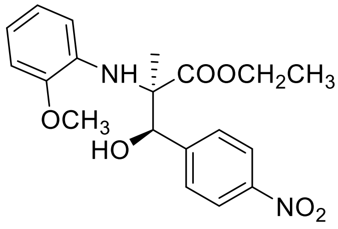

^{1}H NMR (400 MHz, $CDCl_3$, 25 ℃, TMS) *δ* 8.10(d, *J* = 8.0 Hz, 2H), 7.41(d, *J* = 8.0 Hz, 2H), 6.86-6.76(m, *J* = 8.0 Hz, 3H), 6.68 (m, 2H), 5.60 (d, *J* = 4.0 Hz, 1H), 4.45(m, 2H), 4.22-4.16 (d, *J* = 8.0 Hz, 2H), 3.80 (s, 3H), 1.39 (s, 3H), 1.11 (t, *J* = 8.0 Hz, 3H). **^{13}C NMR** (100 MHz, $CDCl_3$, 25 ℃, TMS) *δ* 176.59, 147.41, 146.06, 134.51, 128.54, 122.40, 121.01, 118.60, 112.40, 110.20, 73.11, 63.13, 62.00, 55.50, 19.45, 13.81. **HRMS** (ESI) Calcd for $C_{19}H_{22}N_2O_6$ $(M+Na)^+$ 397.1376 , Found: 397.1385.

Ethyl(2*R**,3*R**)-2-propyl-3-hydroxy-2-((2-methoxyphenyl)amino)-2-methyl-3 -(4-nitrophenyl) propanoate (4c)

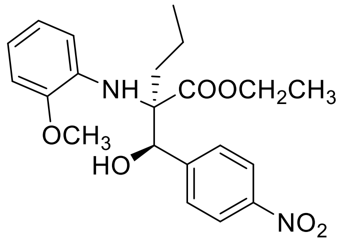

^{1}H NMR (400 MHz, $CDCl_3$, 25 °C, TMS) δ 8.12 (d, *J* = 8.4 Hz, 2H), 7.43 (d, J = 8.3 Hz, 2H), 6.82-6.72 (m, *J* = 8.0 Hz, 3H), 6.43 (m, 2H), 5.42 (s, 1H), 5.34 (d, *J* = 8.3 Hz, 1H), 4.20 (q, *J* = 7.0 Hz, 2H), 3.85 (s, 3H), 3.71 (d, *J* = 8.5 Hz, 1H), 2.16 – 1.99 (m, 2H), 1.25 (m, *J* = 4.1 Hz, 5H), 0.79 (t, *J* = 7.2 Hz, 3H). **^{13}C NMR** (100 MHz, $CDCl_3$, 25 ℃, TMS) *δ* 173.41, 147.95, 147.95, 134.25, 128.19, 122.82, 120.76, 118.36, 113.49, 110.10, 75.09, 69.21, 62.36, 55.78, 34.80, 29.70, 14.07. **HRMS** (ESI) Calcd for $C_{21}H_{26}N_2O_6$ $(M+Na)^+$ 425.1325 , Found: 425.1342.

Ethyl (2*R*, 3*R*)-2-benzyl-3-hydroxy-2-((2-methoxyphenyl)amino)-3-(2-nitrophenyl) propanoate (4d)

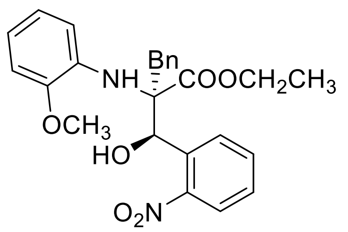

^{1}H NMR (400 MHz, $CDCl_3$, 25 ℃, TMS) *δ* 7.94 (s, 1H), 7.82 (s, 1H), 7.57 (s, 1H), 7.44 (s, 1H), 7.25(s, 1H), 6.84-7.11 (m, 9H), 6.31 (s, 1H), 5.57 (s, 1H), 3.86 (s, 1H), 3.78 (s, 3H), 3.75 (s, 1H), 3.42 (dd, *J* = 7.5 Hz, 1H), 3.20 (dd, *J* = 12 Hz, 1H), 0.86 (t, *J* = 8.0 Hz, 3H). **^{13}C NMR** (100 MHz, $CDCl_3$, 25 ℃, TMS) *δ* 172.26, 149.43, 148.42, 135.61, 134.28, 133.76, 131.94, 130.58, 129.94, 128.51, 128.02, 126.79, 124.09, 121.06, 118.94, 114.60, 110.45, 70.35, 61.70, 59.91, 36.54, 29.69, 13.34. **HRMS** (ESI) Calcd for $C_{21}H_{26}N_2O_6$ $(M+Na)^+$ 473.1655 Found: 473.1689 **HPLC** (Chiral IA, λ = 254 nm, hexane/2-propanol = 5:1, Flow rate = 0.8 mL/min), t_R= 11.98 min, 15.46 min.

Ethyl (2*R, 3*R**)-2-benzyl-3-(4-cyanophenyl)-3-hydroxy-2-((2-methoxyphenyl) amino) propanoate (4e)**

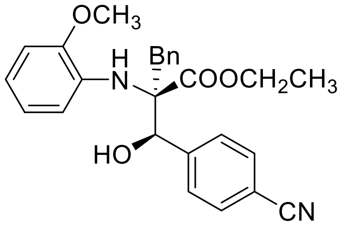

^{1}H NMR (400 MHz, $CDCl_3$, 25 ℃, TMS) *δ* 7.58 (d, *J* = 8.0 Hz, 2H), 7.45 (d, *J* = 8.0 Hz, 2H), 7.18 (m, 3H), 6.97 (s, 2H), 6.84-6.81 (m, 2H), 6.78-6.73 (m, 2H), 5.61 (s, 1H), 5.10 (s, 1H), 4.49 (s, 1H), 3.96 (m, *J* = 4.0 Hz, 1H), 3.77(s, 3H), 3.46 (d, *J* = 16.0 Hz, 1H), 2.96 (d, *J* = 16.0 Hz, 1H), 0.84 (t, *J* =

8.0 Hz, 3H). **^{13}C NMR** (100 MHz, $CDCl_3$, 25 ℃, TMS) *δ* 174.16, 147.66, 144.30, 135.16, 134.55, 131.17, 129.95, 128.54, 128.06, 126.95, 120.84, 118.85, 118.30, 112.98, 111.39, 110.36, 74.27, 68.03, 61.64, 55.69, 37.53, 13.34. **HRMS** (ESI) Calcd for $C_{26}H_{26}N_2O_4$ $(M+Na)^+$ 453.1790, Found: 453.1768.

Ethyl (2*R, 3*R**)-2-benzyl-3-(4-bromophenyl)-3-hydroxy-2-((2-methoxyphenyl) amino)propanoate (4f)**

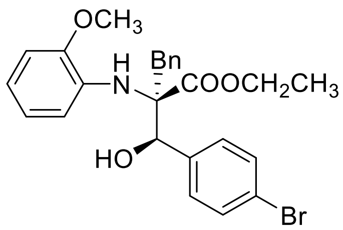

^{1}H NMR (400 MHz, $CDCl_3$, 25 ℃, TMS) *δ* 7.36 (d, *J* = 12.0 Hz, 2H), 7.14 (d, *J* = 12.0 Hz, 2H),7.08 (m, 3H), 6.94 (m, 2H), 6.81-6.84 (m, 2H), 6.71-6.76 (m, 2H), 5.26-5.32 (m, 2H), 4.04-3.97 (m, 2H), 3.85 (d, *J* = 8.0 Hz, 1H), 3.72-3.68 (m, 4H), 3.53-3.49 (d, *J* = 8.0 Hz, 1H),1.05 (t, *J* = 8.0 Hz, 3H). **^{13}C NMR** (100 MHz, $CDCl_3$, 25 ℃, TMS) *δ* 172.54, 147.86, 138.86, 135.66, 134.73, 130.77, 130.19, 128.78, 127.89, 126.68, 121.75, 120.90, 117.75, 113.40, 110.47, 74.45, 69.65, 61.93, 55.78, 37.76, 13.75. **HRMS** (ESI) Calcd for $C_{25}H_{26}BrNO_4$ $(M+Na)^+$ 506.0943 , Found: 506.0952. **HPLC** (Chiral IA, λ = 254 nm, hexane/2-propanol = 5:1, Flow rate = 0.8 mL/min), t_R= 9.83 min, 10.57 min.

Ethyl (2*R, 3*R**)-2-benzyl-3-(3-bromophenyl)-3-hydroxy-2-((2-methoxyphenyl) amino)propanoate (4g)**

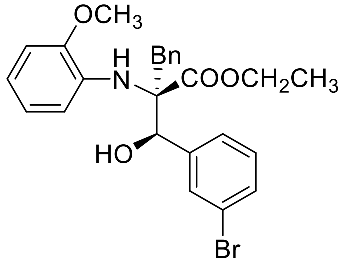

^{1}H NMR (400 MHz, $CDCl_3$, 25 ℃, TMS) *δ* 7.47-7.42 (m, 2H), 7.29 (s, 1H), 7.16 (d, *J* = 7.4 Hz, 4H), 7.00 (s, 2H), 6.86-6.82 (m, 2H), 6.79-6.73 (m, *J* = 6.5 Hz, 4H), 5.47 (s, 1H), 5.15 (s, 1H), 4.27 (s, 1H), 3.98-3.96 (m, 1H), 3.87-3.80 (m, 4H), 3.46 (d, *J* = 14.2 Hz, 1H), 3.03 (d, *J* = 16 Hz, 4H), 0.91 (t, *J*= 7.1 Hz, 3H). **^{13}C NMR** (100 MHz, $CDCl_3$, 25 ℃, TMS) *δ* 173.23, 146.80, 140.17, 133.63, 129.84, 129.10, 128.06, 127.05, 125.84, 125.49, 120.83, 119.86, 117.24, 112.18, 109.40, 73.28, 67.31, 60.66, 54.75, 48.28, 36.57, 34.60, 28.69, 27.46, 12.44. **HRMS** (ESI) Calcd. for $C_{25}H_{26}BrNO_4$ $(M+Na)^+$ 506.0943, Found: 506.0936.

Ethyl (2*R*[*], 3*R*[*])-2-benzyl-3-hydroxy-2-((2-methoxyphenyl)amino)-3-phenylpropanoate (4h)

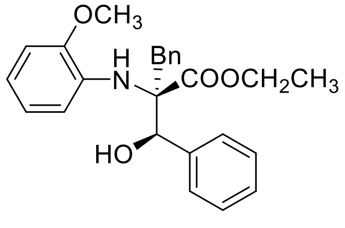

^{1}H NMR (400 MHz, $CDCl_3$, 25 ℃, TMS) *δ* 7.48-7.39 (m, 2H), 7.29 (s, 1H), 7.18 (dd, *J* = 6.0, 2.1 Hz, 4H), 7.00 (dd, *J* = 6.4, 2.8 Hz, 2H), 6.87-6.80 (m, 2H), 6.76 (dd, *J* = 15.4, 7.0 Hz, 2H), 5.47 (s, 1H), 5.15 (s, 1H), 4.26 (s, 1H), 3.98 (dd, *J* = 10.7, 7.2 Hz, 1H), 3.86 (dt, *J* = 6.6, 5.5 Hz, 1H), 3.80 (s, 3H), 3.46 (d, *J* = 14.2 Hz, 1H), 3.03 (d, *J* = 14.2 Hz, 1H), 0.91 (t, *J* = 7.2 Hz, 4H). **^{13}C NMR** (100 MHz, CDCl3, 25 ℃, TMS) *δ* 172.19, 146.18, 135.54, 128.27, 127.37, 125.81, 120.12, 116.53, 109.52, 108.93, 59.90, 56.81, 54.48, 37.86, 13.05. **HRMS** (ESI) Calcd. for $C_{25}H_{27}NO_4$ $(M+Na)^+$ 428.1838, Found: 428.1828.

Ethyl (2*R*[*], 3*R*[*])-2-benzyl-3-hydroxy-2-(mesitylamino)-3-(4-nitrophenyl) propanoate (4i)

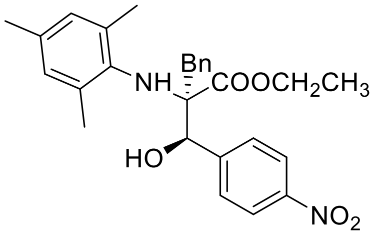

^{1}H NMR (400 MHz, $CDCl_3$, 25 ℃, TMS) *δ* 8.20 (d, *J* = 8.0 Hz, 2H), 7.60 (d, *J* = 8.0 Hz, 2H), 7.05 (d, *J* = 4.0 Hz, 1H), 6.92-6.96 (m, *J* = 8.0 Hz, 2H), 6.72(s, 2H), 6.55 (d, *J* = 8.0 Hz, 2H), 5.12(s, 1H), 4.74(s, 2H), 4.11-4.07 (m, 1H), 3.88-3.83 (m, 1H), 3.34 (d, *J* = 16.0 Hz, 1H), 3.00 (d, *J* = 16.0 Hz, 1H), 2.24 (s, 3H), 2.11 (s, 6H), 0.97 (t, *J*=8.0 Hz, 3H). **^{13}C NMR** (100 MHz, $CDCl_3$, 25 ℃, TMS) *δ* 172.13, 148.19, 147.31, 139.32, 135.11, 133.66, 133.64, 129.84, 129.75, 128.51, 127.51, 126.51, 122.67, 72.36, 61.67, 38.78, 20.47, 19.96, 13.44. **HRMS** (ESI) Calcd for $C_{27}H_{30}N_2O_5$ $(M+Na)^+$, Found: 428.1828.

Ethyl (2*R*[*], 3*R*[*])-2-benzyl-3-hydroxy-3-(4-nitrophenyl)-2-(phenylamino) propanoate (4j)

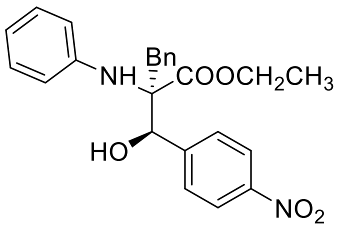

^{1}H NMR (400 MHz, $CDCl_3$, 25 ℃, TMS) *δ* 8.09(d, *J* = 7.0 Hz, 2H), 7.41 (d, *J* = 7.0 Hz, 2H), 7.23 (m, *J* =13.1 Hz, 2H), 7.17-7.15 (m, 3H), 6.97 (m, 2H),

6.83(m,*J* = 5.8 Hz, 2H), 5.59 (d, *J* =7.3 Hz, 1H), 4.58(s, 1H), 4.06-3.99 (m, 3H), 3.83 (d, *J* = 11.3 Hz, 1H), 3.56 (d, *J* = 11.3 Hz, 1H), 1.09 (t, *J* = 5.7Hz, 3H). **^{13}C NMR** (100 MHz, $CDCl_3$, 25 ℃, TMS) *δ* 172.02, 147.50, 147.34, 144.08, 135.23, 130.19, 129.64, 128.17, 127.97, 127.07, 122.85, 118.69, 115.73, 73.89, 69.84, 62.51, 36.71, 29.69, 13.88. **HRMS** (ESI) Calcd for $C_{24}H_{24}N_2O_5$ $(M+Na)^+$ 443.1583, Found: 443.1568 .

Ethyl (2*R, 3*R**)-2-benzyl-2-((4-ethoxyphenyl)amino)-3-hydroxy-3-(4-nitrophenyl) propanoate (4k)**

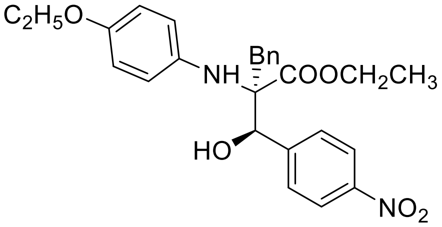

^{1}H NMR (400 MHz, $CDCl_3$, 25 ℃, TMS) *δ* 8.10 (d, *J* = 8.8 Hz, 2H), 7.49 (d, *J* = 8.7 Hz, 2H), 7.13 (d, *J* = 3.0 Hz, 3H), 6.91-6.86 (m, 2H), 6.75-6.70 (m, 2H), 6.63-6.58 (m, 2H), 5.51 (s, 1H), 4.26 (s, 1H), 4.21 (s, 1H), 3.96-3.88 (m, 3H), 3.77 (m, 1H), 3.36 (d, *J* = 14.2 Hz, 1H), 2.94 (d, *J* = 14.2 Hz, 1H), 1.33 (t, *J* = 7.0 Hz, 3H), 0.83 (t, *J* = 7.2 Hz, 3H). **^{13}C NMR** (100 MHz, $CDCl_3$, 25 ℃, TMS) *δ* 172.91, 151.66, 146.55, 145.60, 136.99, 134.19, 129.00, 127.79, 127.26, 126.11, 121.65, 116.85, 115.43, 114.69, 114.60, 112.38, 73.77, 67.81, 62.92, 60.79, 36.49, 28.69, 13.94, 12.49. **HRMS** (ESI) Calcd for $C_{26}H_{28}N_2O_6$ $(M+Na)^+$ 487.1845, Found: 487.1859.

Ethyl (2R, 3R)-2-benzyl-3-hydroxy-3-(4-nitrophenyl)-2-((3,4,5-trimethoxyphenyl) amino) propanoate (4l)

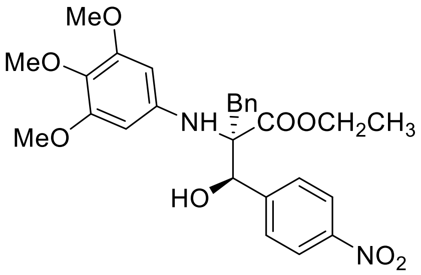

^{1}H NMR (400 MHz, $CDCl_3$, 25 ℃, TMS) δ 8.10 (d, *J* = 8.4 Hz, 2H), 7.48 (d, *J* = 8.8 Hz, 2H), 7.14(t, *J* =3.2 Hz, 3H), 6.91(t, *J* =3.2 Hz, 2H), 5.92(s, 2H), 5.55(d, *J* = 2.4 Hz, 1H), 4.33 (s, 1H), 4.02 (d, *J* = 2.4 Hz, 1H), 3.72-3.97 (m, 2H), 3.73 (s,6H), 3.72 (s, 3H), 3.38 (d, *J* = 14.0 Hz, 1H), 3.06 (d, *J* = 14.0 Hz, 1H), 0.91 (t, *J* = 6.8 Hz, 3H). **^{13}C NMR** (100 MHz, $CDCl_3$, 25 ℃, TMS) δ 172.87, 152.89, 146.62, 145.39, 140.07, 134.11, 130.48, 129.04, 127.78, 127.32, 127.21, 126.22, 121.75, 93.15, 74.17, 67.74, 61.06, 60.10, 55.12, 36.68, 12.64. **HPLC** (Chiral IA, λ = 254 nm, hexane/2-propanol = 5:1, Flow rate = 0.8 mL/min), t_R= 13.06 min, 15.35 min. **HRMS** (ESI) Calcd for $C_{27}H_{30}N_2O_8$ $(M+Na)^+$ 511.2080 Found: 511.2043

Ethyl (2*R, 3*R**)-2-benzyl-3-hydroxy-2-((4-methoxyphenyl)amino)-3 -(4 -nitrophenyl) propanoate (4m)**

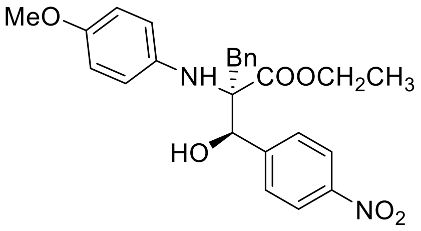

^{1}H NMR (400 MHz, $CDCl_3$, 25 ℃, TMS) δ 8.14 (d, *J* = 8.0 Hz, 2H), 7.54 (d, *J* = 8.7 Hz, 2H), 7.20(d, *J* = 4.0 Hz, 3H), 6.96(s, 2H), 6.81-6.78 (m, 2H), 6.69 (m, 2H), 5.58(s, 1H), 4.40 (s, 1H), 4.33 (s, 1H), 4.01-3.96 (m, 1H), 3.87-3.82 (m, 1H), 3.76 (s,3H), 3.41 (d, *J* = 12.0 Hz, 1H), 3.04 (d, *J* = 12.0 Hz, 1H), 0.90 (t, *J* = 8.0 Hz, 3H). **^{13}C NMR** (100 MHz, $CDCl_3$, 25 ℃, TMS) δ 173.79, 153.13, 147.37, 146.58, 137.99, 135.13, 129.90, 128.70, 128.17, 127.02, 122.57, 117.69, 114.71, 74.65, 68.73, 61.75, 55.33, 37.33, 13.44. **HRMS** (ESI) Calcd for $C_{25}H_{26}N_2O_6$ $(M+Na)^+$ 473.1689, Found: 473.1694 .

Ethyl (2*R, 3*R**)-2-benzyl-2-((2-ethoxyphenyl)amino)-3-hydroxy-3 -(4 -nitrophenyl) propanoate(4n)**

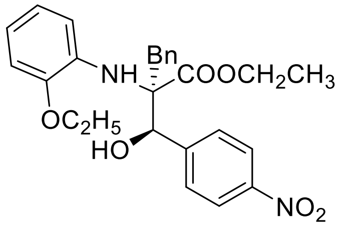

^{1}H NMR (400 MHz, $CDCl_3$, 25 ℃, TMS) δ 8.09 (d, *J* = 8.6 Hz, 2H), 7.43 (d, *J* = 8.6 Hz, 2H), 7.16 (d, *J* = 5.1 Hz, 3H), 6.98-6.93 (m, 2H), 6.88-6.74 (m, 4H), 5.50 (d, *J* = 8.8 Hz, 1H), 5.35 (s, 1H), 4.27 (d, *J* = 8.9 Hz, 1H), 4.02 –

3.87 (m, 4H), 3.75 (d, J = 14.0 Hz, 1H), 3.52 (d, J = 14.0 Hz, 1H), 1.22 (t, J = 6.9 Hz, 3H), 1.01 (t, J = 7.1 Hz, 3H). **^{13}C NMR** (100 MHz, $CDCl_3$, 25 ℃, TMS) δ 171.55, 146.51, 146.28, 134.27, 133.51, 129.25, 127.20, 127.01, 125.92, 121.69, 119.93, 117.29, 112.45, 110.64, 73.43, 68.56, 63.20, 61.12, 36.17, 28.69, 13.83, 12.69. **HRMS** (ESI) Calcd for $C_{26}H_{28}N_2O_6$ $(M+Na)^+$ 487.1845 , Found: 487.1861.

Ethyl (*R)-2-((*R**)-hydroxy(4-nitrophenyl)methyl)-2-(mesitylamino) pentanoate (4o)**

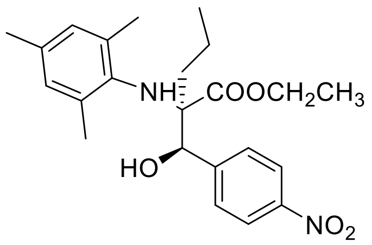

^{1}H NMR (400 MHz, $CDCl_3$, 25 ℃, TMS) δ 7.69 (d, J = 8.0 Hz, 2H), 7.06 (d, J = 8.0 Hz, 2H), 6.35(s, 2H), 4.70 (s, 1H), 4.38 (dd, J = 12 Hz, 1H), 3.54-3.49 (m, 1H), 3.07-3.03 (m, 1H), 1.81 (s, 6H), 1.75 (s, 3H), 0.37-0.33 (m, 4H), 0.18 (s, 1H), 0.74-0.58 (m, 1H), 0.00-0.02 (m, 3H). **^{13}C NMR** (100 MHz, $CDCl_3$, 25 ℃, TMS) δ 172.61, 148.27, 147.37, 139.12, 133.46, 133.03, 129.76, 128.18, 122.75, 71.63, 63.31, 33.75, 20.52, 19.91, 17.18, 14.40, 13.44. **HRMS** (ESI) Calcd for $C_{23}H_{30}N_2O_5$ $(M+Na)^+$ 435.1896 , Found: 435.1793.

***tert*-Butyl (2*R**, 3*R**)-3-hydroxy-2-((2-methoxyphenyl)amino)-3-(4-nitrophenyl) propanoate (4p)**

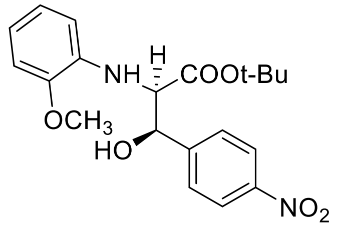

Chemical Formula: $C_{20}H_{24}N_2O_6$
Exact Mass: 388.16

^{1}H NMR (400 MHz, $CDCl_3$, 25 ℃, TMS)δ 8.22 (dd, J = 12.0, 8.8 Hz, 2H), 7.62 (d, J = 8.1 Hz, 2H), 6.85-6.69 (m, 3H), 6.44 (d, J = 8.3 Hz, 1H), 5.15 (dd, J = 41.7, 24.8 Hz, 2H), 3.87 (s, 3H), 3.43 (d, J = 5.2 Hz, 1H), 1.33 (s, 6H), 1.24 (s, 3H). **^{13}C NMR** (100 MHz, $CDCl_3$, 25 ℃, TMS) δ 170.59, 147.72, 147.67, 147.46, 136.15, 127.59, 127.11, 123.43, 123.35, 121.01, 118.93, 111.65, 110.08, 83.16, 73.46, 73.13, 63.82, 55.62, 27.88. **HRMS** (ESI) Calcd for $C_{20}H_{24}N_2O_6$ $(M+Na)^+$ 413.1689 , Found: 413.1529.

Single Crystal X-ray Structure Determinations of Compound *threo*-4o

CCDC No.: 984118

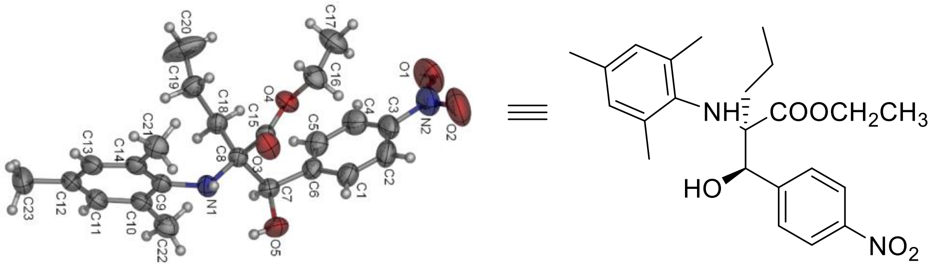

(2*R**, 3*R**)-*threo*-**4o**

Bond precision: C-C = 0.0032 A Wavelength=0.71073

Cell: a=12.0803(4) b=14.8270(5) c=13.7314(5)

alpha=90 beta=114.984(1) gamma=90

Temperature: 296 K

	Calculated	Reported
Volume	2229.35(13)	2229.35(13)
Space group	P 21/c	P2(1)/c
Hall group	-P 2ybc	?
Moiety formula	C23 H30 N2 O5	?
Sum formula	C23 H30 N2 O5	C23 H30 N2 O5
Mr	414.49	414.49
Dx,g cm-3	1.235	1.235
Z	4	4
Mu (mm-1)	0.087	0.087
F000	888.0	888.0
F000'	888.43	
h,k,lmax	14,17,16	14,17,16
Nref	3934	3936
Tmin,Tmax	0.973, 0.983	0.973,0.983

Correction method= MULTI-SCAN

Data completeness= 1.001 Theta(max)= 25.000

R(reflections)= 0.0470(2847) wR2(reflections)= 0.1483(3936)

S = 1.038 Npar= 276

^{1}H NMR and ^{13}C NMR spectra for new compounds

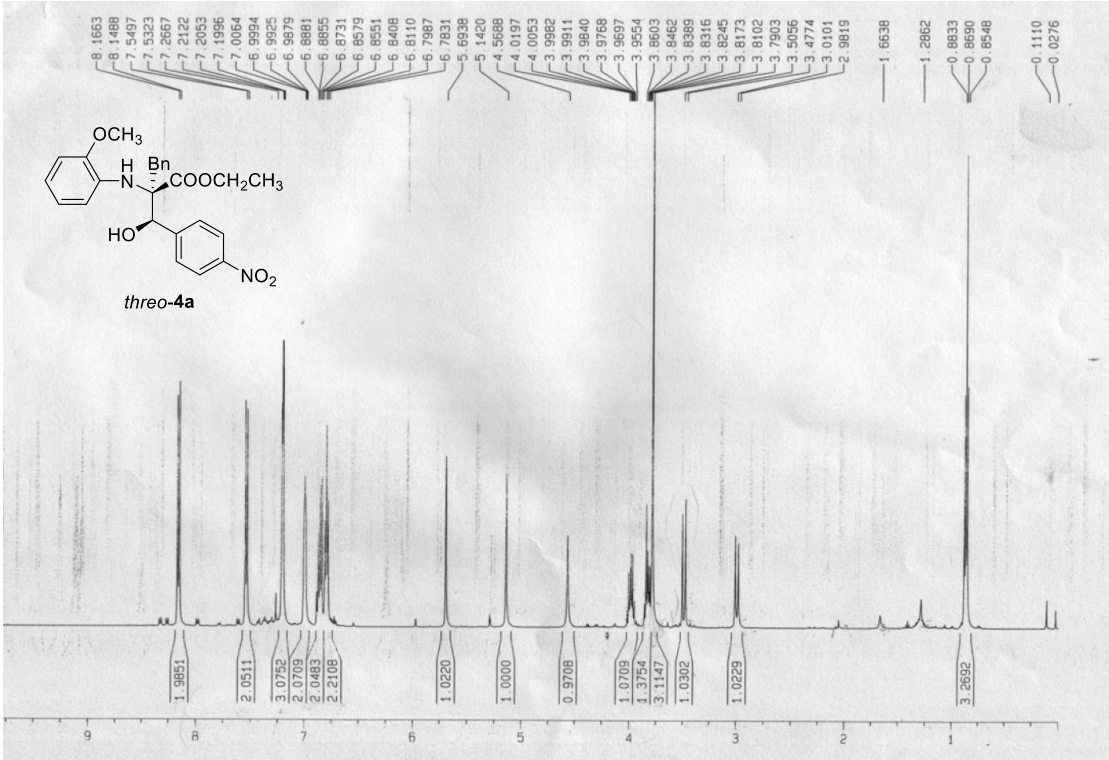

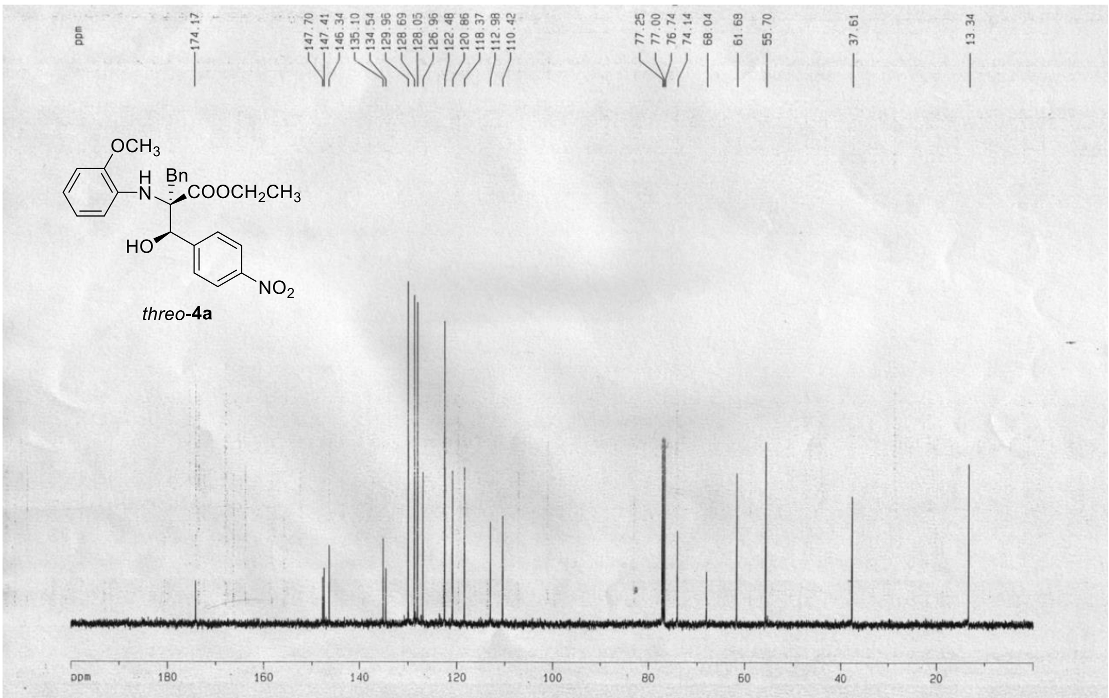
threo-4a

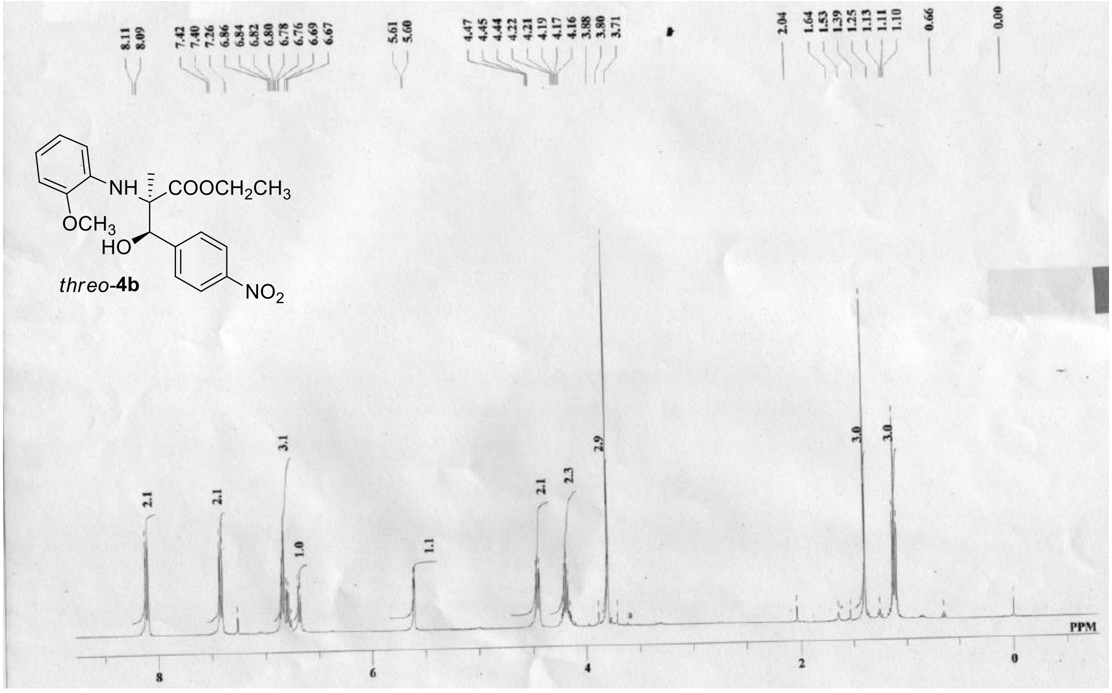
threo-4b

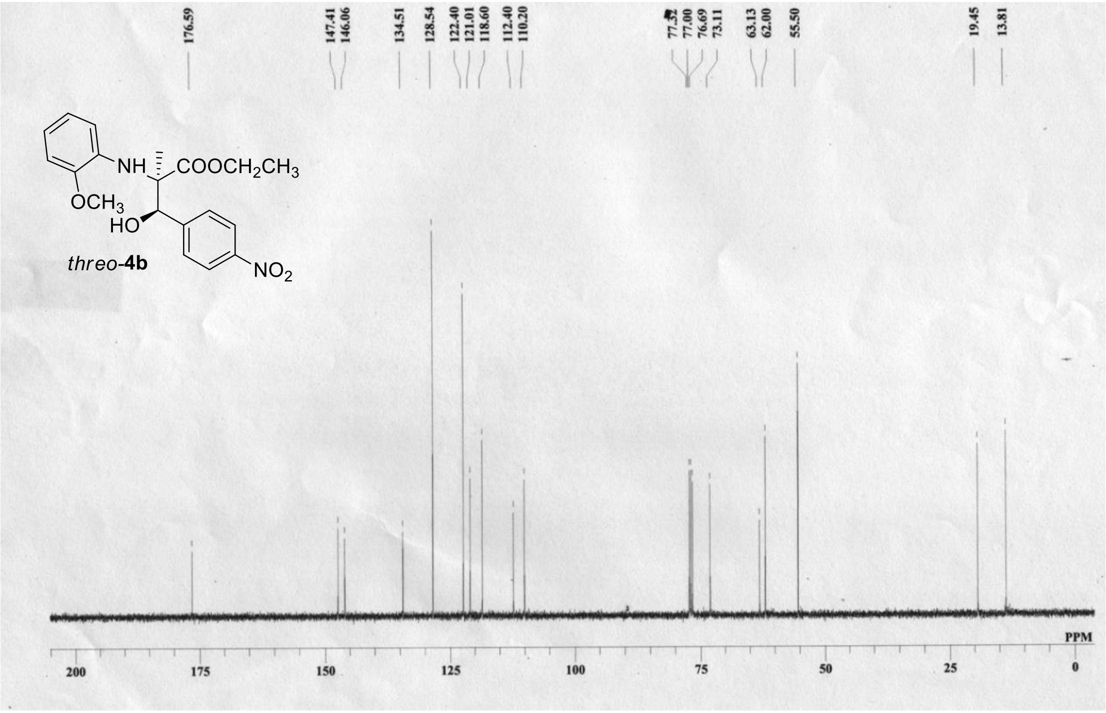
threo-4b
NH
COOCH2CH3
OCH3
HO
NO2
176.59
147.41
146.06
134.51
128.54
122.40
121.01
118.60
112.40
110.20
77.00
76.69
73.11
63.13
62.00
55.50
19.45
13.81
PPM

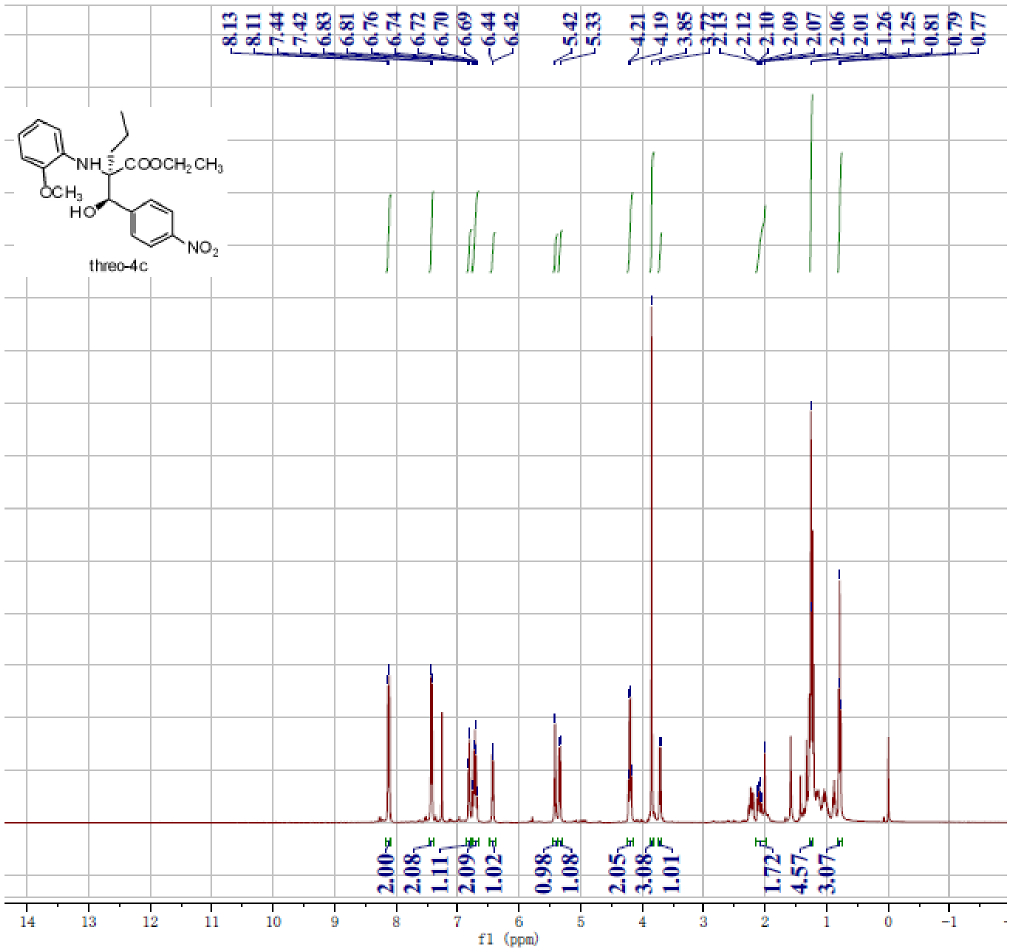
threo-4c
NH
COOCH2CH3
OCH3
HO
NO2
8.13
8.11
7.44
7.42
6.83
6.81
6.76
6.74
6.72
6.70
6.69
6.44
6.42
5.42
5.33
4.21
4.19
3.85
2.13
2.12
2.10
2.09
2.07
2.06
2.01
1.26
1.25
0.81
0.79
0.77
2.00
2.08
1.11
2.09
1.02
0.98
1.08
2.05
3.08
1.01
1.72
4.57
3.07
f1 (ppm)

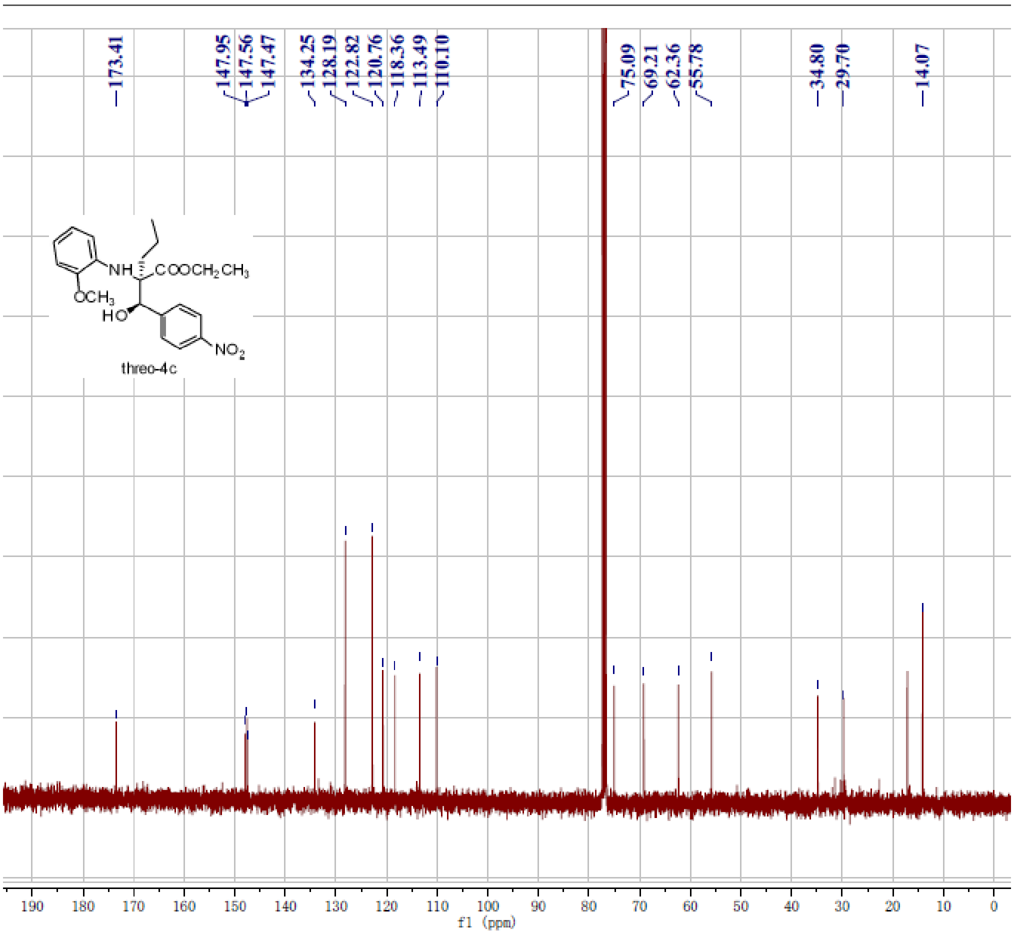
173.41
147.95
147.56
147.47
134.25
128.19
122.82
120.76
118.36
113.49
110.10
75.09
69.21
62.36
55.78
34.80
29.70
14.07
NH
COOCH2CH3
OCH3
HO
NO2
threo-4c
190
180
170
160
150
140
130
120
110
100
90
80
70
60
50
40
30
20
10
0
f1 (ppm)

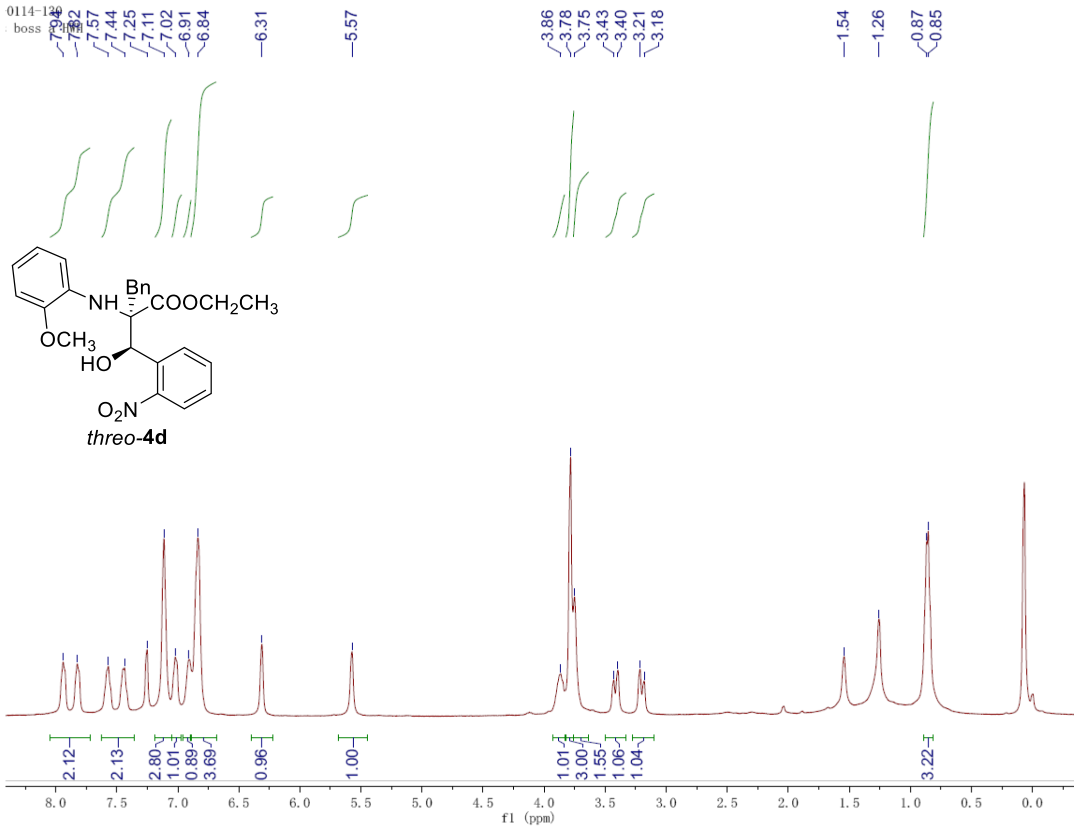
7.94 7.82 7.57 7.44 7.25 7.11 7.02 6.91 6.84
6.31
5.57
3.86 3.78 3.75 3.43 3.40 3.21 3.18
1.54
1.26
0.87 0.85
NH
Bn
COOCH2CH3
OCH3
HO
O2N
threo-4d
2.12 2.13 2.80 1.01 0.89 3.69 0.96 1.00 1.01 3.00 1.55 1.06 1.04 3.22
8.0 7.5 7.0 6.5 6.0 5.5 5.0 4.5 4.0 3.5 3.0 2.5 2.0 1.5 1.0 0.5 0.0
f1 (ppm)

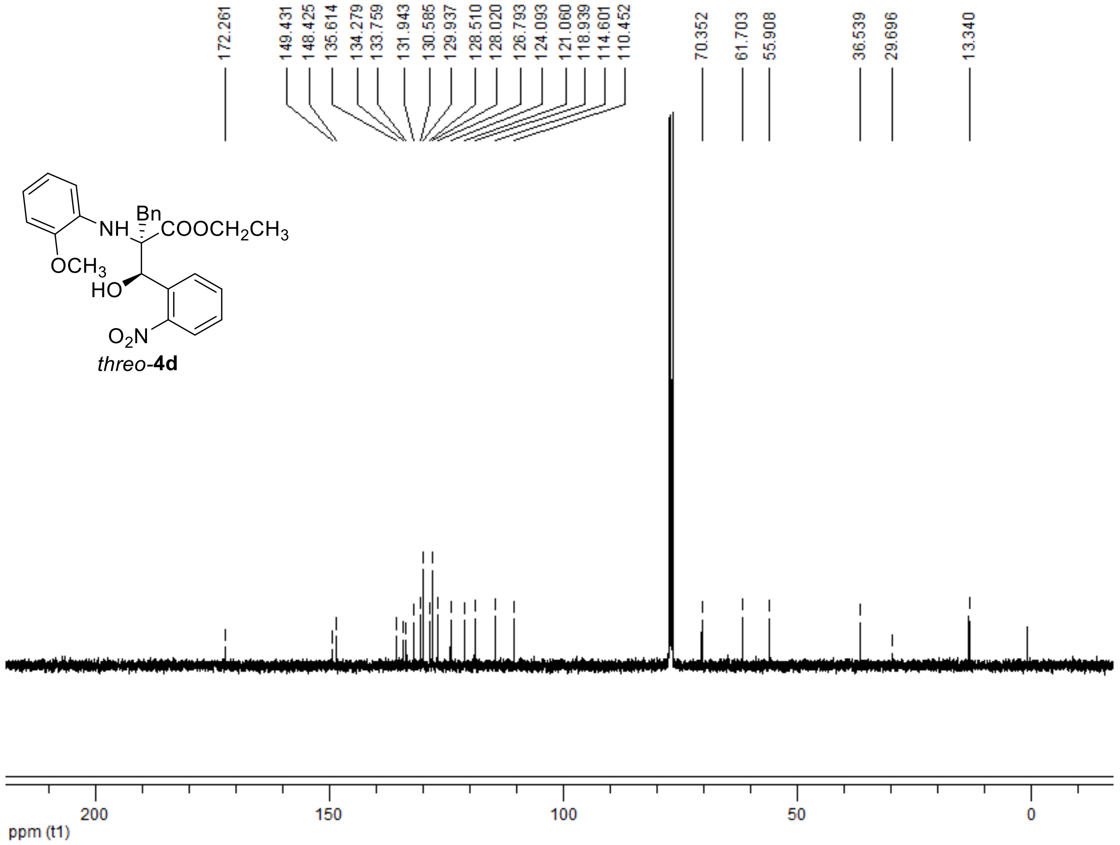
172.261
149.431 148.425 135.614 134.279 133.759 131.943 130.585 129.937 128.510 128.020 126.793 124.093 121.060 118.939 114.601 110.452
70.352 61.703 55.908
36.539 29.696 13.340
NH
Bn
COOCH2CH3
OCH3
HO
O2N
threo-4d
200 150 100 50 0
ppm (t1)

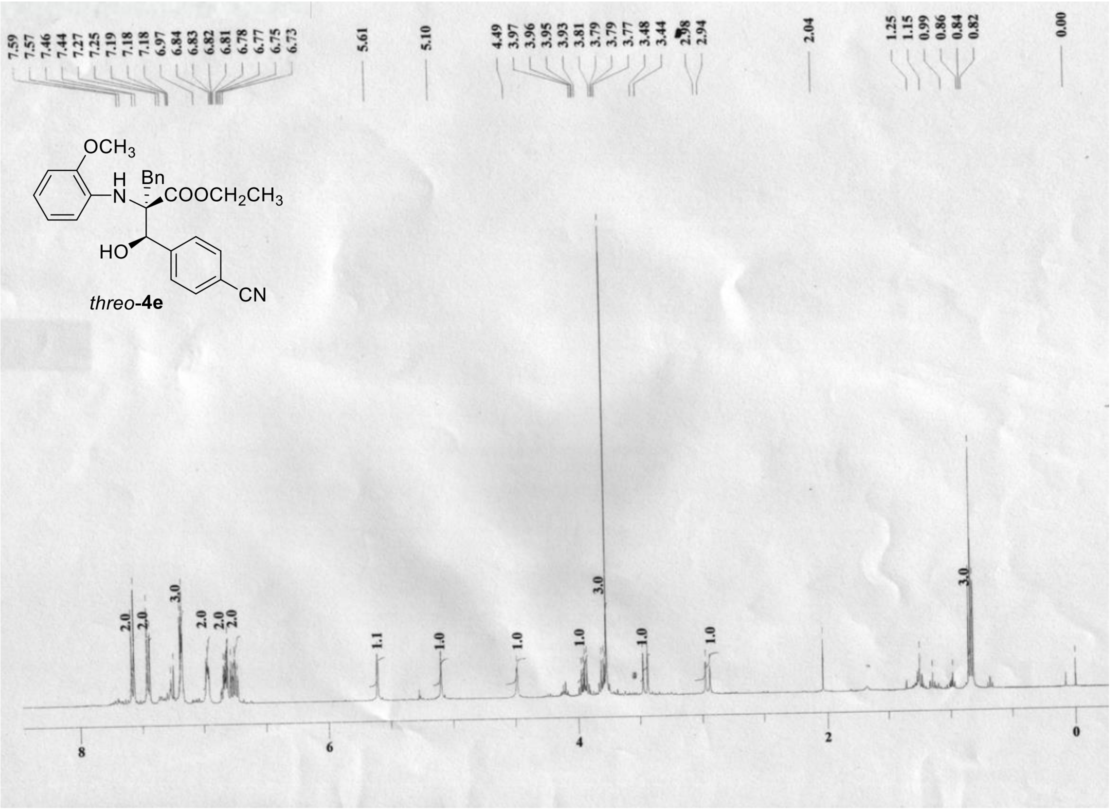

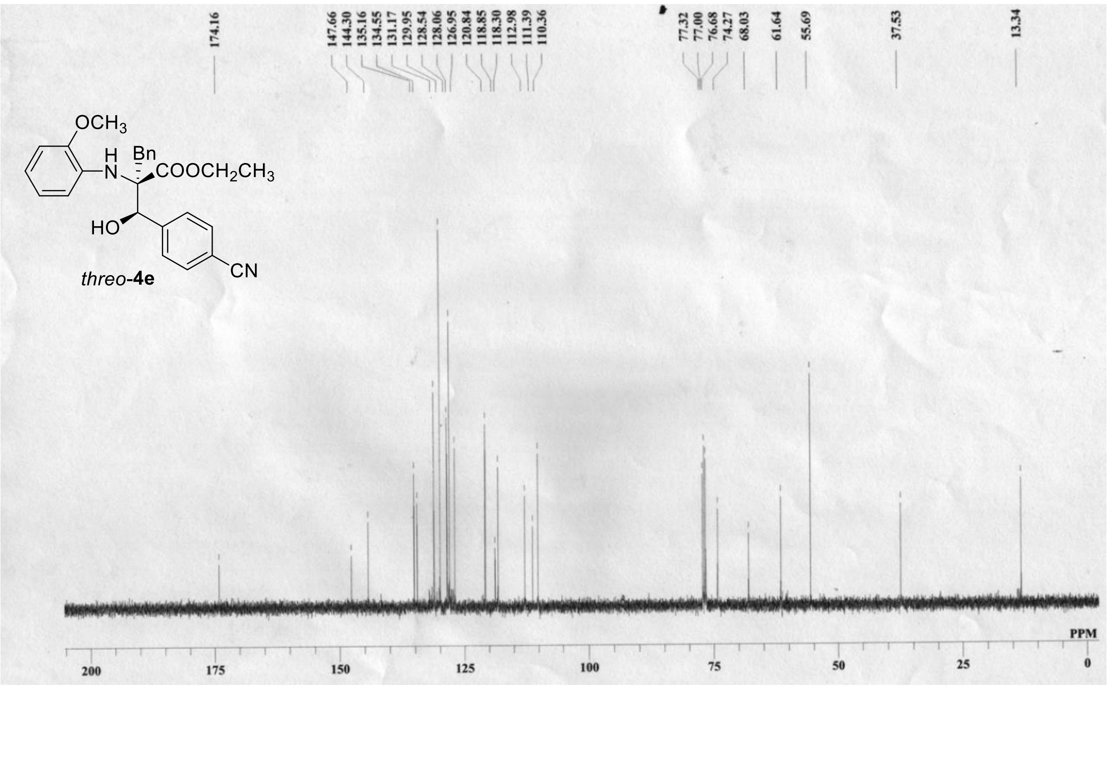

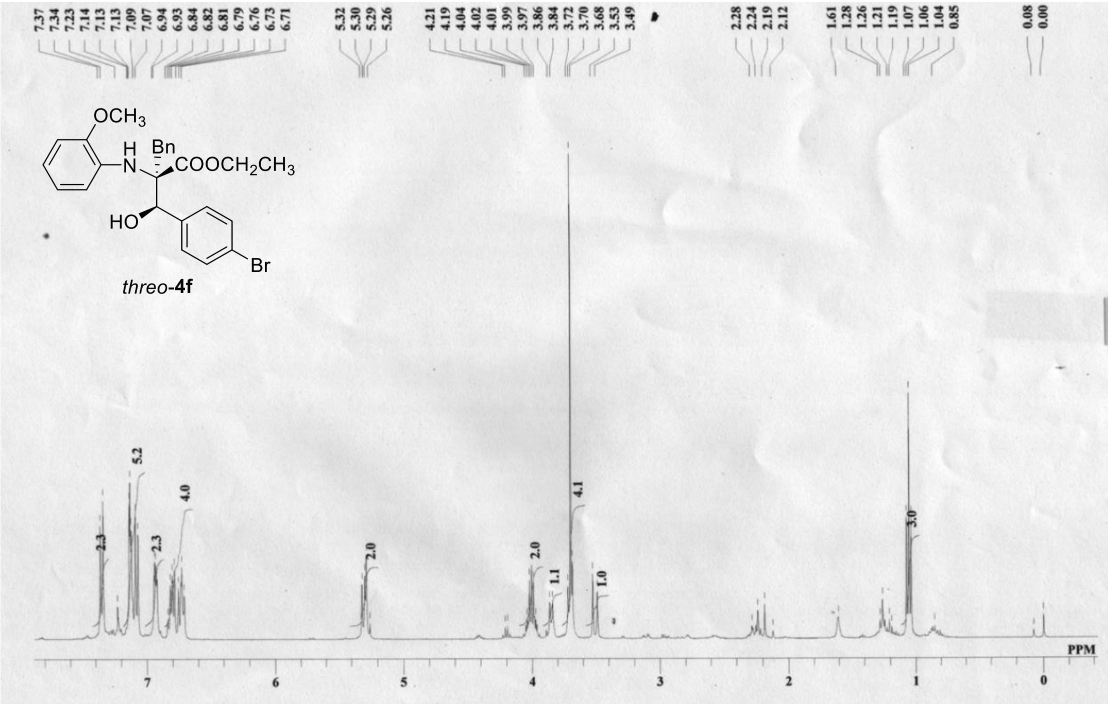
7.37 7.34 7.23 7.14 7.13 7.13 7.09 7.07 6.94 6.93 6.84 6.82 6.81 6.79 6.76 6.73 6.71
5.32 5.30 5.29 5.26
4.21 4.19 4.04 4.02 4.01 3.99 3.97 3.86 3.84 3.72 3.70 3.68 3.53 3.49
2.28 2.24 2.19 2.12
1.61 1.28 1.26 1.21 1.19 1.07 1.06 1.04 0.85
0.08 0.00
OCH3
H
Bn
N
COOCH2CH3
HO
Br
threo-4f
2.3
5.2
2.3
4.0
2.0
2.0
1.1
4.1
1.0
3.0
PPM
7
6
5
4
3
2
1
0

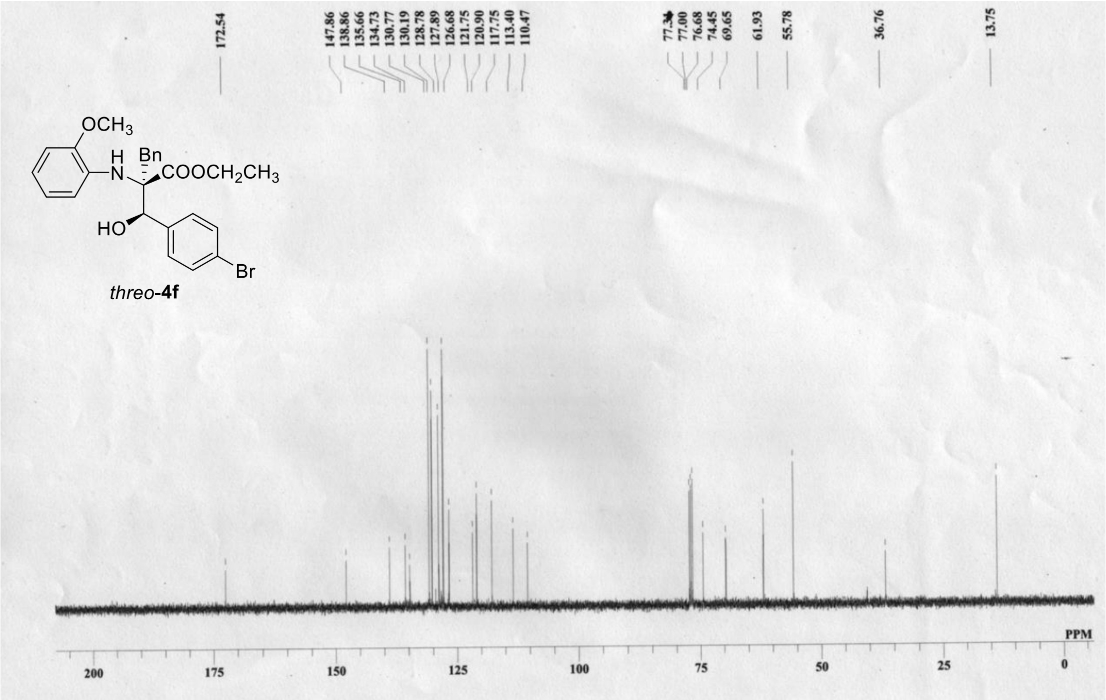
172.54
147.86 138.86 135.66 134.73 130.77 130.19 128.78 127.89 126.68 121.75 120.90 117.75 113.40 110.47
77.32 77.00 76.68 74.45 69.65 61.93 55.78
36.76
13.75
OCH3
H
Bn
N
COOCH2CH3
HO
Br
threo-4f
PPM
200
175
150
125
100
75
50
25
0

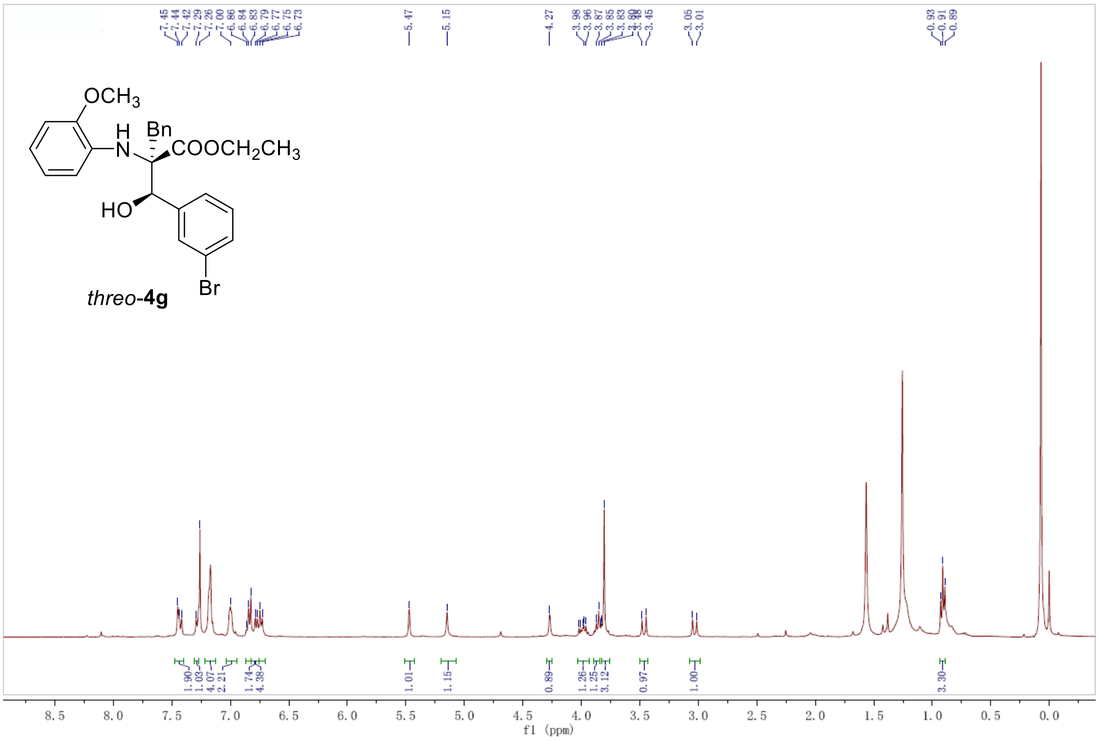
OCH3
H
N
Bn
COOCH2CH3
HO
Br
threo-4g
f1 (ppm)

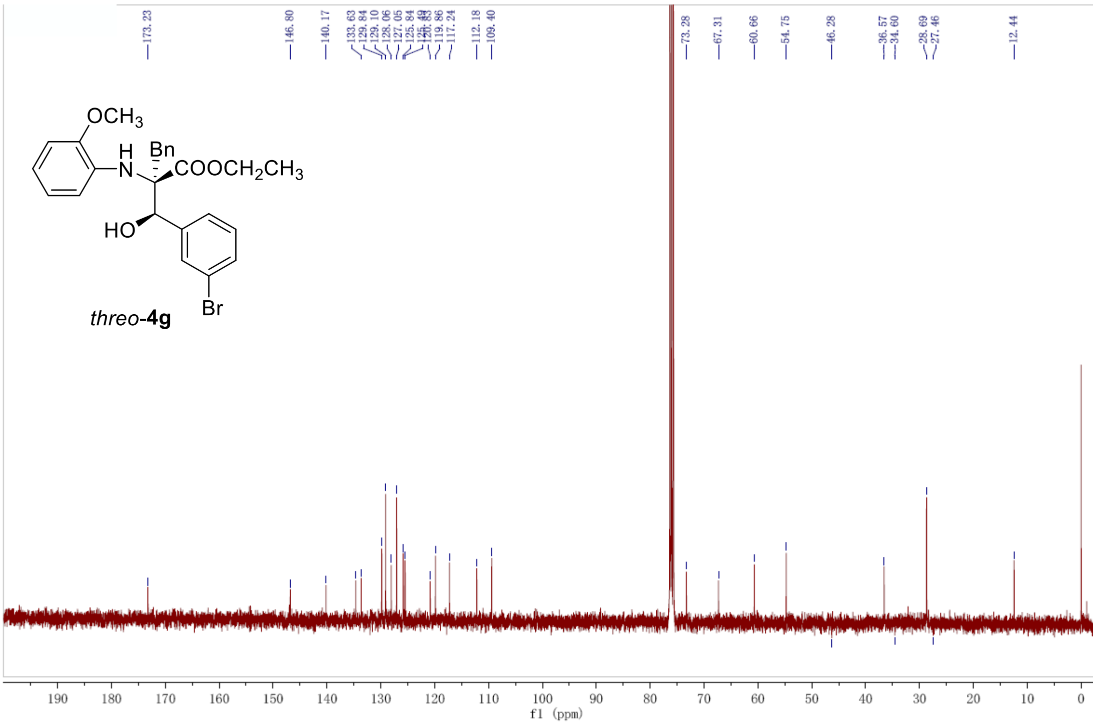
OCH3
H
N
Bn
COOCH2CH3
HO
Br
threo-4g
f1 (ppm)

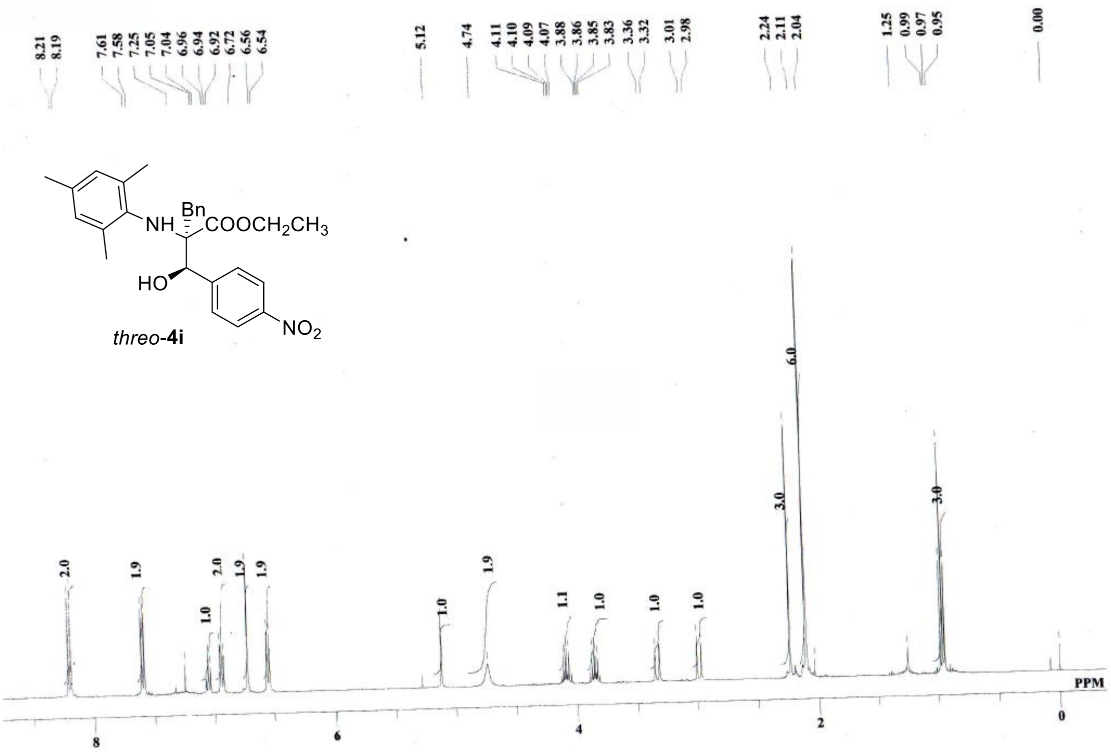
8.21 8.19 7.61 7.58 7.25 7.05 7.04 6.96 6.94 6.92 6.72 6.56 6.54 5.12 4.74 4.11 4.10 4.09 4.07 3.88 3.86 3.85 3.83 3.36 3.32 3.01 2.98 2.24 2.11 2.04 1.25 0.99 0.97 0.95 0.00
Bn
NH
COOCH2CH3
HO
NO2
threo-4i
2.0 1.9 1.0 2.0 1.9 1.9 1.0 1.9 1.1 1.0 1.0 1.0 3.0 6.0 3.0
PPM
8
6
4
2
0

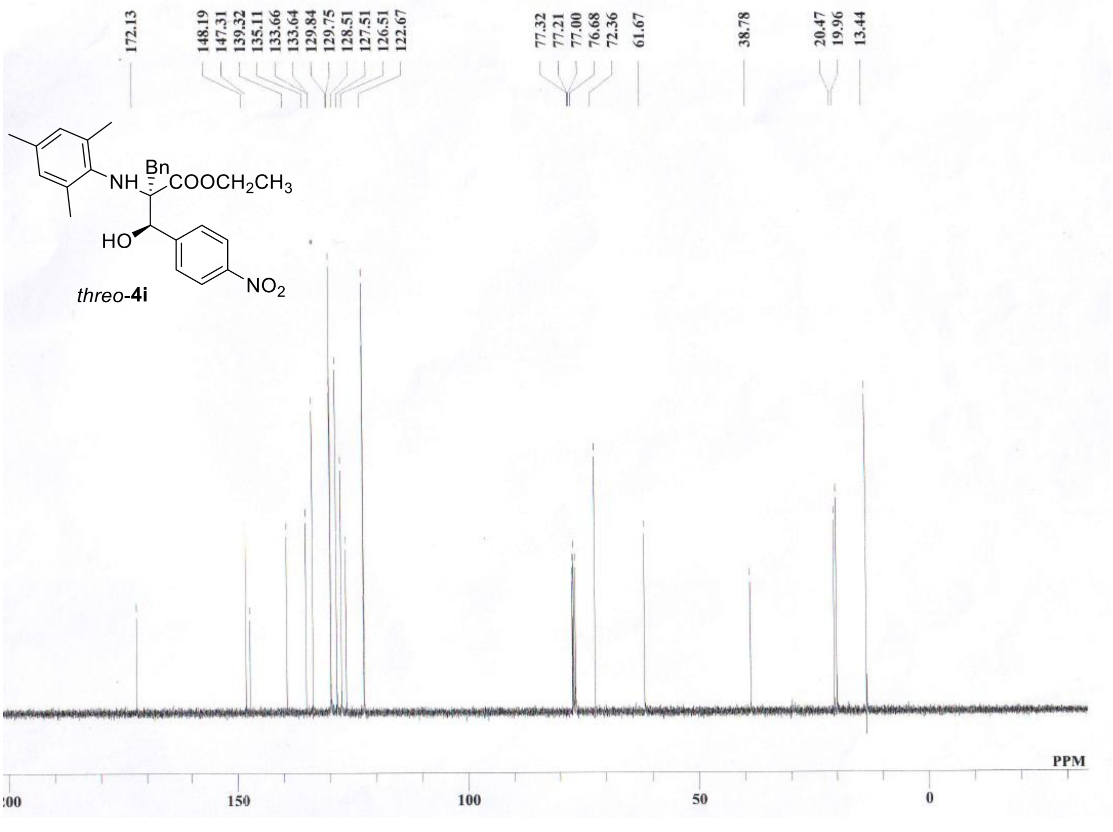
172.13 148.19 147.31 139.32 135.11 133.66 133.64 129.84 129.75 128.51 127.51 126.51 122.67 77.32 77.21 77.00 76.68 72.36 61.67 38.78 20.47 19.96 13.44
Bn
NH
COOCH2CH3
HO
NO2
threo-4i
PPM
150
100
50
0

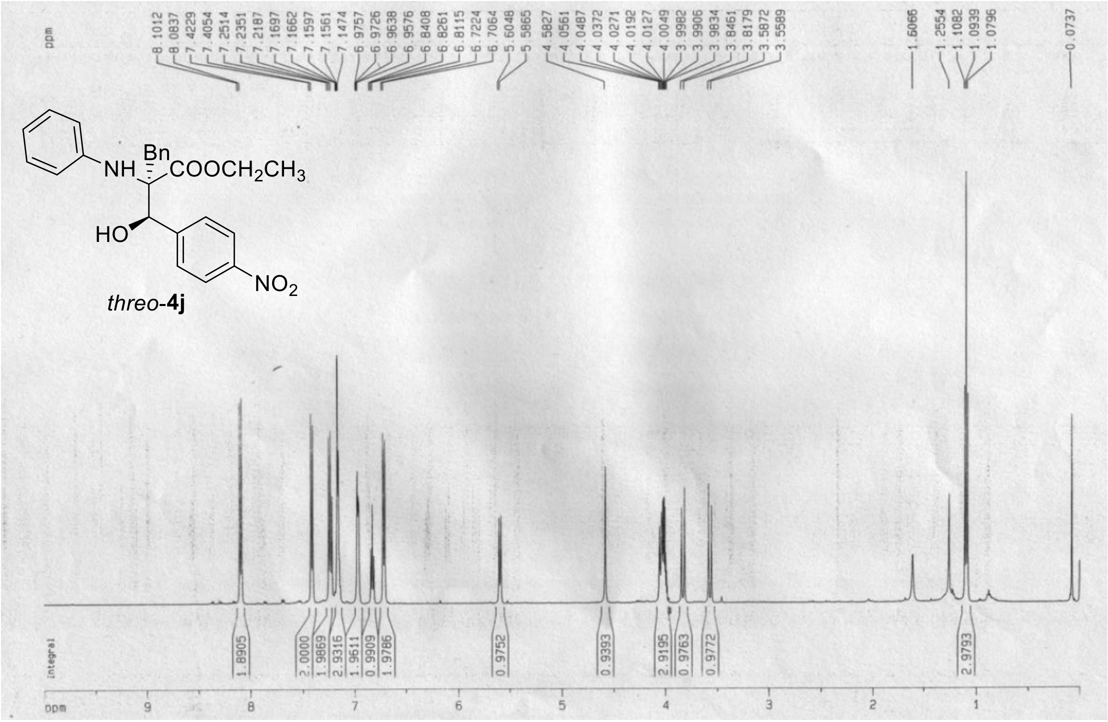
threo-4j
NO2
HO
NH
Bn
COOCH2CH3

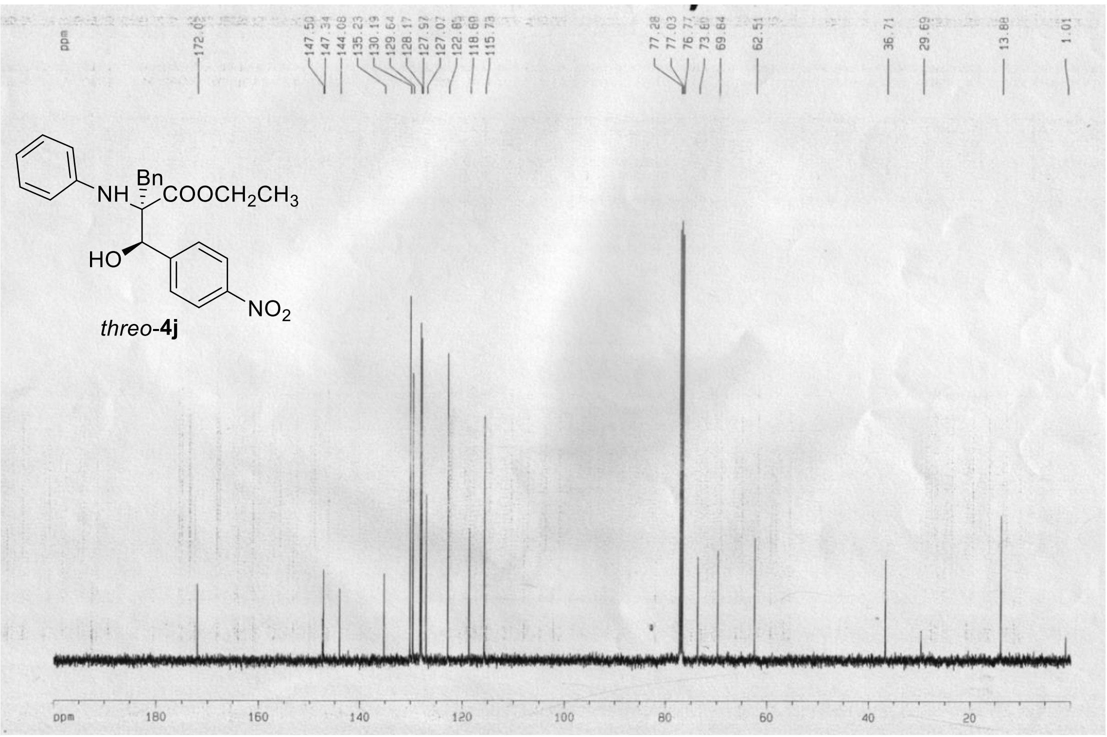
threo-4j
NO2
HO
NH
Bn
COOCH2CH3

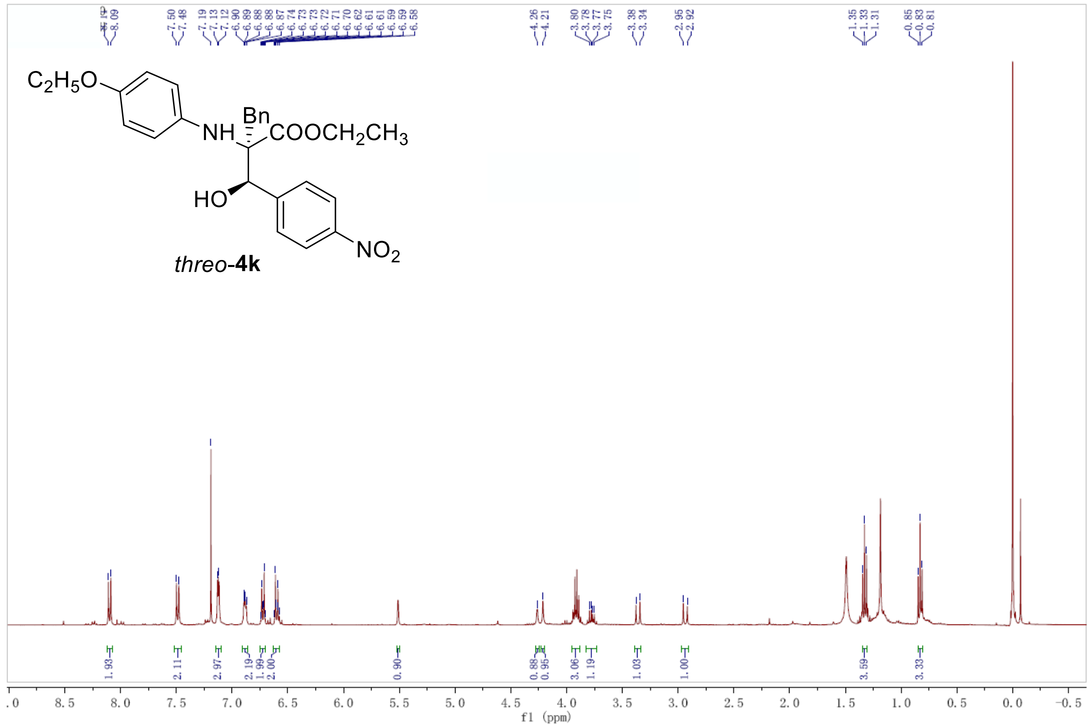
C2H5O
NH
Bn
COOCH2CH3
HO
NO2
threo-4k
f1 (ppm)

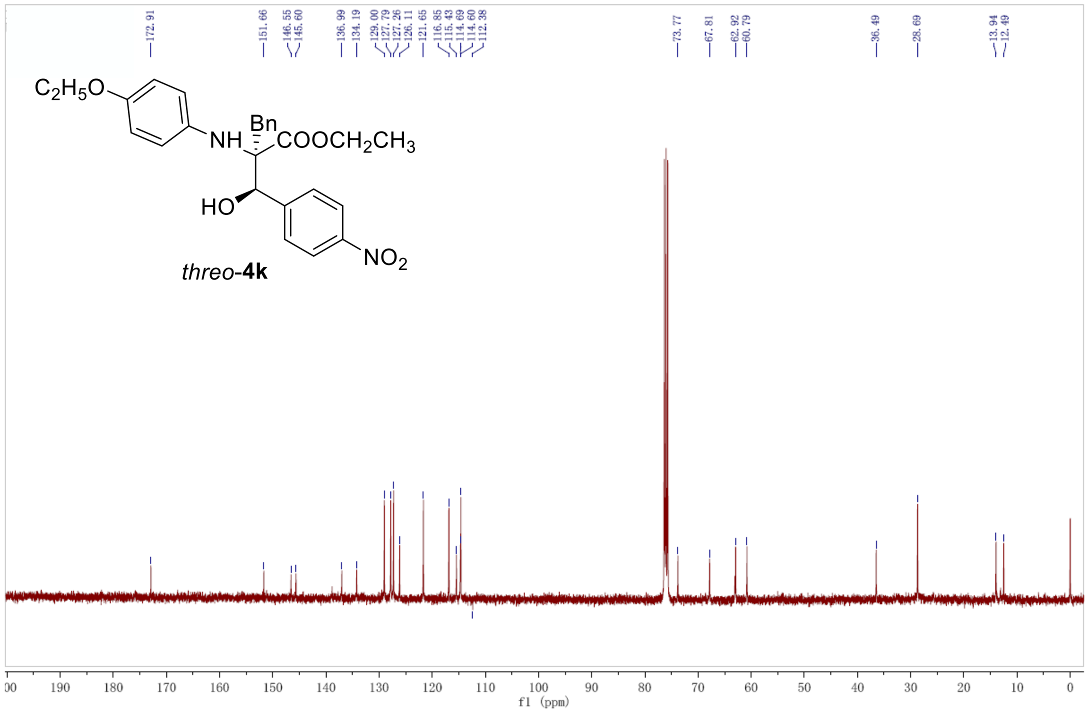
C2H5O
NH
Bn
COOCH2CH3
HO
NO2
threo-4k
f1 (ppm)

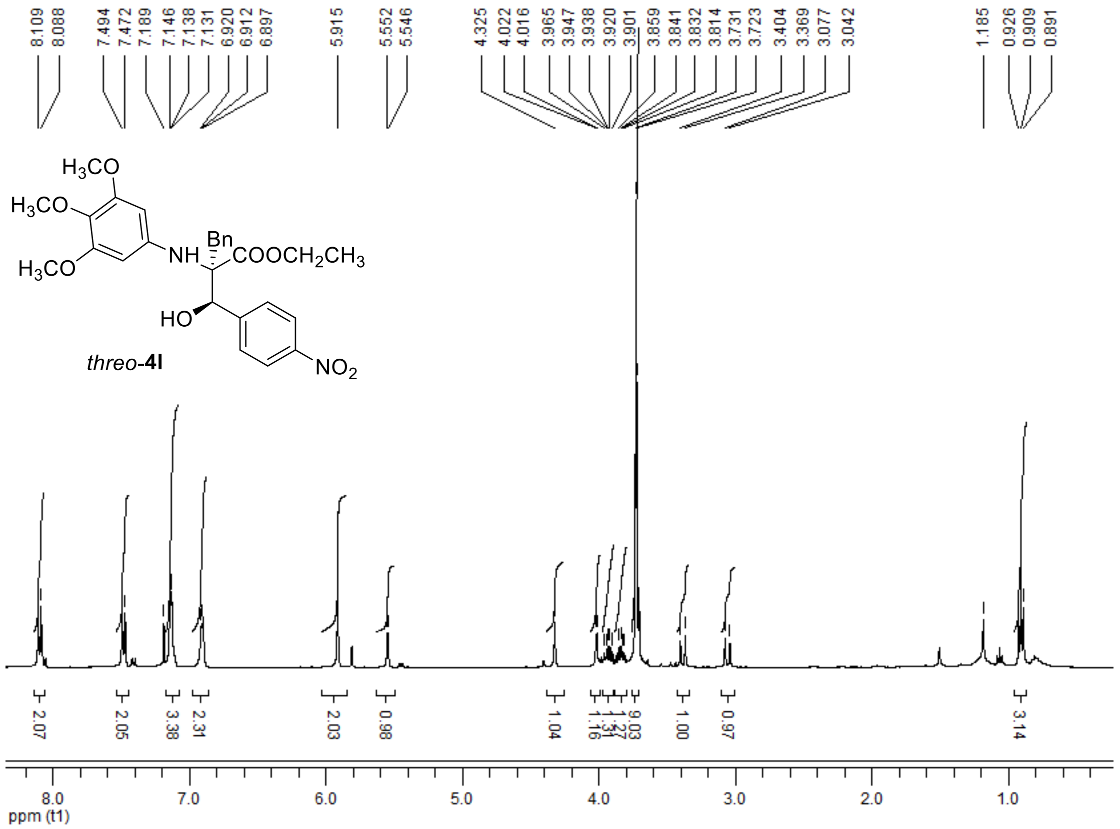
8.109
8.088
7.494
7.472
7.189
7.146
7.138
7.131
6.920
6.912
6.897
5.915
5.552
5.546
4.325
4.022
4.016
3.965
3.947
3.938
3.920
3.901
3.859
3.841
3.832
3.814
3.731
3.723
3.404
3.369
3.077
3.042
1.185
0.926
0.909
0.891
threo-4l
2.07
2.05
3.38
2.31
2.03
0.98
1.04
1.16
1.31
1.27
9.03
1.00
0.97
3.14
ppm (t1)

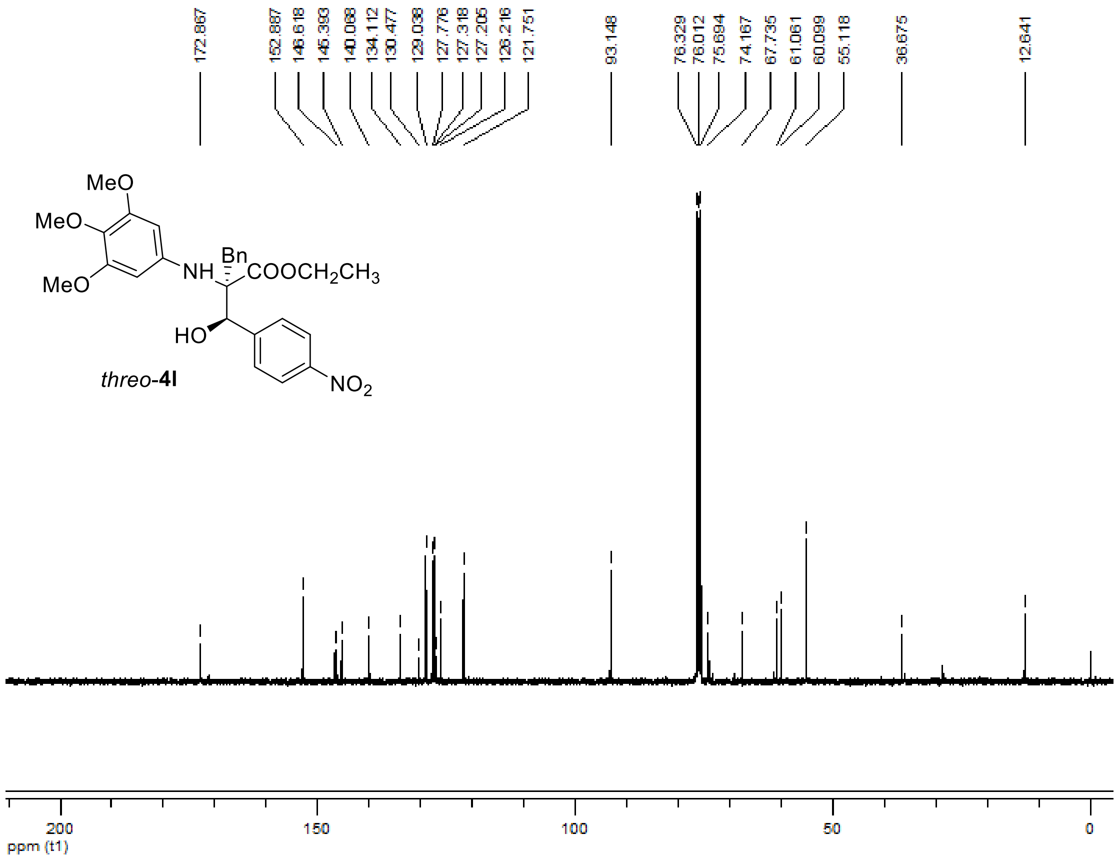
172.867
152.887
146.618
145.393
140.068
134.112
130.477
129.038
127.776
127.318
127.205
126.216
121.751
93.148
76.329
76.012
75.694
74.167
67.735
61.061
60.099
55.118
36.675
12.641
threo-4l
ppm (t1)

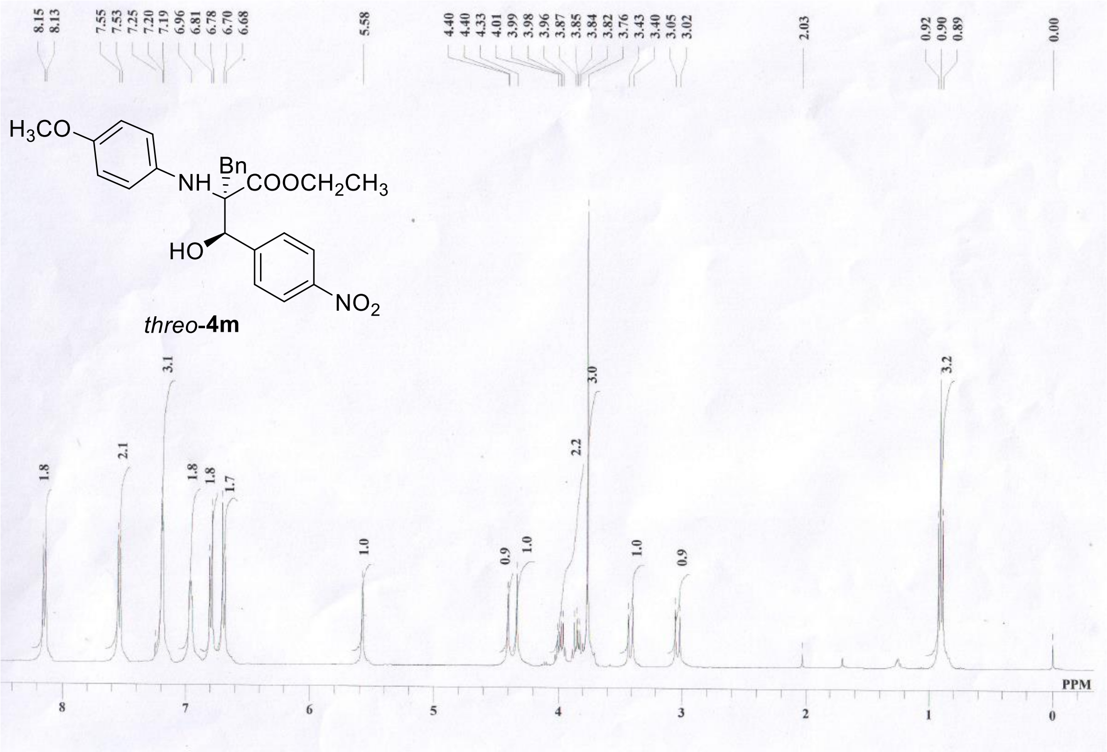
8.15
8.13
7.55
7.53
7.25
7.20
7.19
6.96
6.81
6.78
6.70
6.68
5.58
4.40
4.40
4.33
4.01
3.99
3.98
3.96
3.87
3.85
3.84
3.82
3.76
3.43
3.40
3.05
3.02
2.03
0.92
0.90
0.89
0.00
H3CO
NH
Bn
COOCH2CH3
HO
NO2
threo-4m
1.8
2.1
3.1
1.8
1.8
1.7
1.0
0.9
1.0
2.2
3.0
1.0
0.9
3.2
PPM
8
7
6
5
4
3
2
1
0

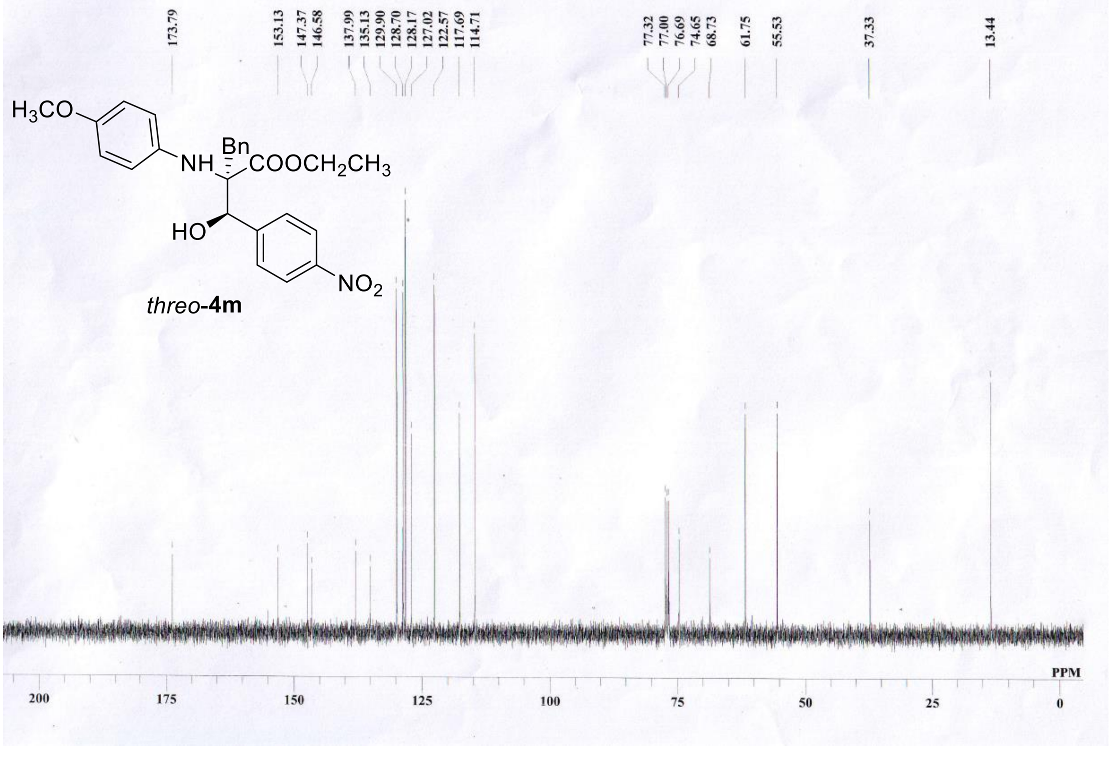
173.79
153.13
147.37
146.58
137.99
135.13
129.90
128.70
128.17
127.02
122.57
117.69
114.71
77.32
77.00
76.69
74.65
68.73
61.75
55.53
37.33
13.44
H3CO
NH
Bn
COOCH2CH3
HO
NO2
threo-4m
PPM
200
175
150
125
100
75
50
25
0

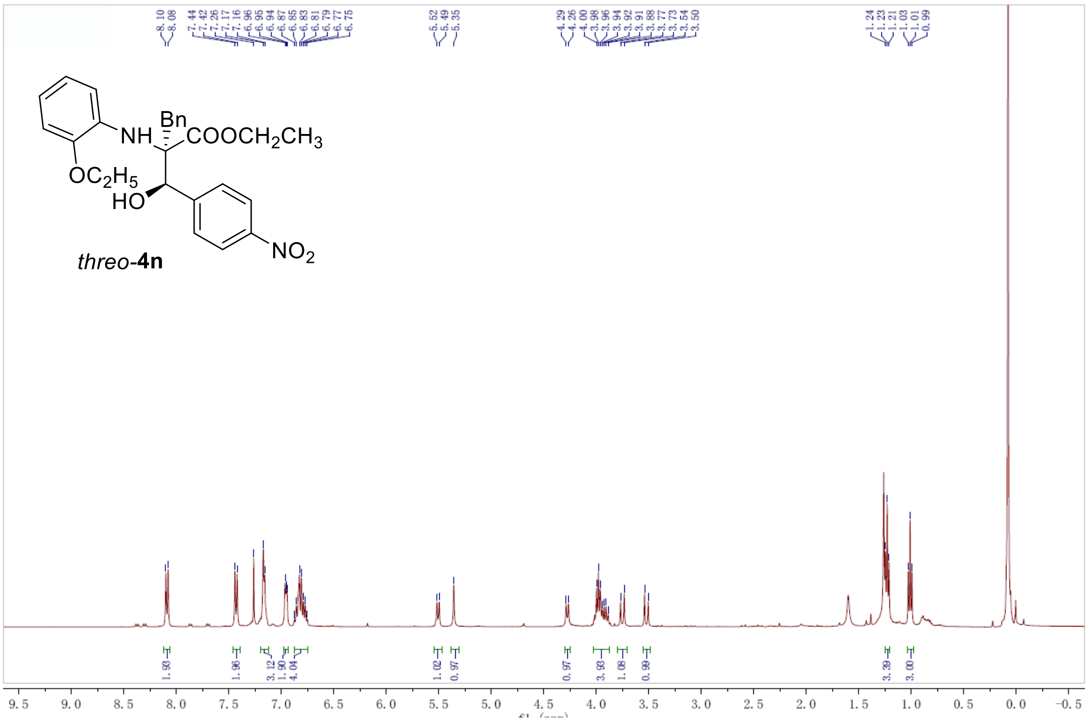
8.10
8.08
7.44
7.42
7.26
7.17
7.16
6.96
6.95
6.94
6.87
6.85
6.83
6.81
6.79
6.77
6.75
5.52
5.49
5.35
4.29
4.26
4.00
3.98
3.96
3.94
3.92
3.91
3.88
3.77
3.73
3.54
3.50
1.24
1.23
1.21
1.03
1.01
0.99
Bn
NH
COOCH2CH3
OC2H5
HO
NO2
threo-4n
1.93
1.96
3.12
1.90
4.04
1.02
0.97
0.97
3.93
1.08
0.99
3.39
3.00

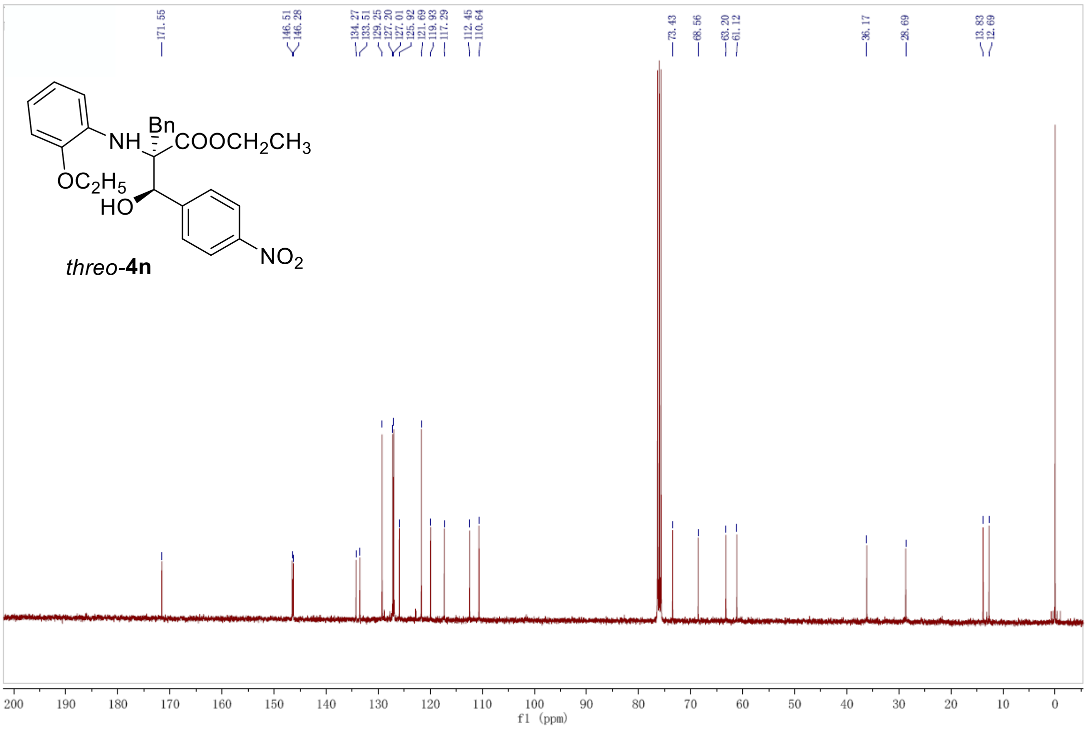
171.55
146.51
146.28
134.27
133.51
129.25
127.20
127.01
125.92
121.69
119.93
117.29
112.45
110.64
73.43
68.56
63.20
61.12
36.17
28.69
13.83
12.69
Bn
NH
COOCH2CH3
OC2H5
HO
NO2
threo-4n
f1 (ppm)

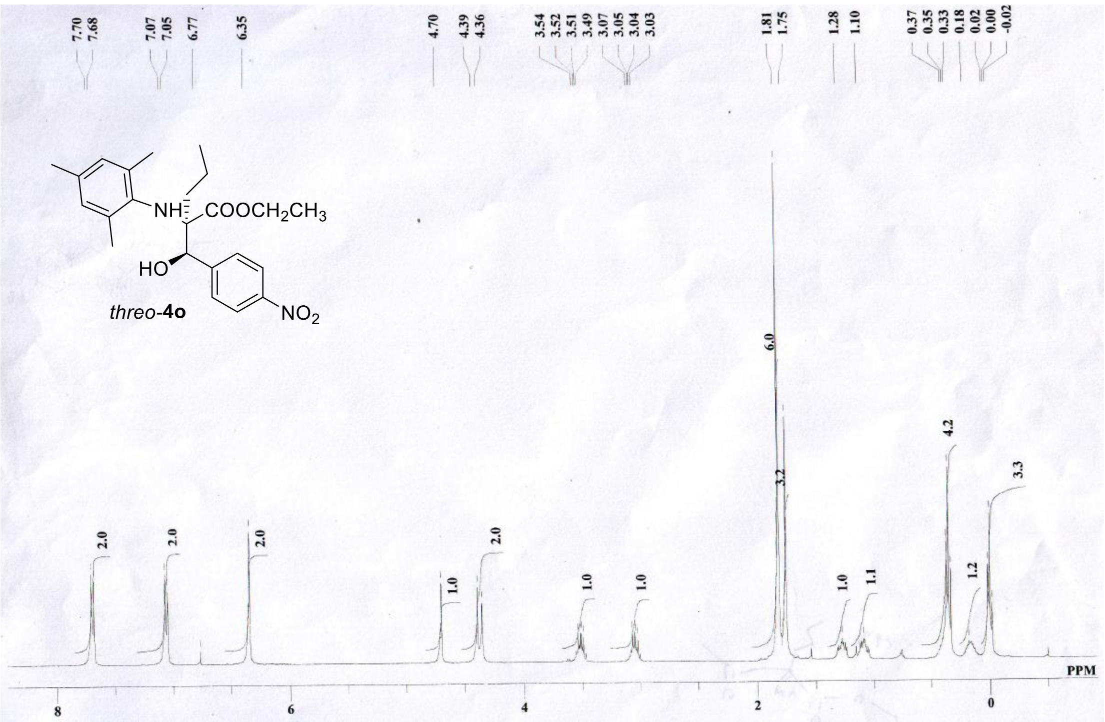
7.70
7.68
7.07
7.05
6.77
6.35
4.70
4.39
4.36
3.54
3.52
3.51
3.49
3.07
3.05
3.04
3.03
1.81
1.75
1.28
1.10
0.37
0.35
0.33
0.18
0.02
0.00
-0.02
NH
COOCH2CH3
HO
NO2
threo-4o
2.0
2.0
2.0
1.0
2.0
1.0
1.0
6.0
3.2
1.0
1.1
4.2
1.2
3.3
PPM
8
6
4
2
0

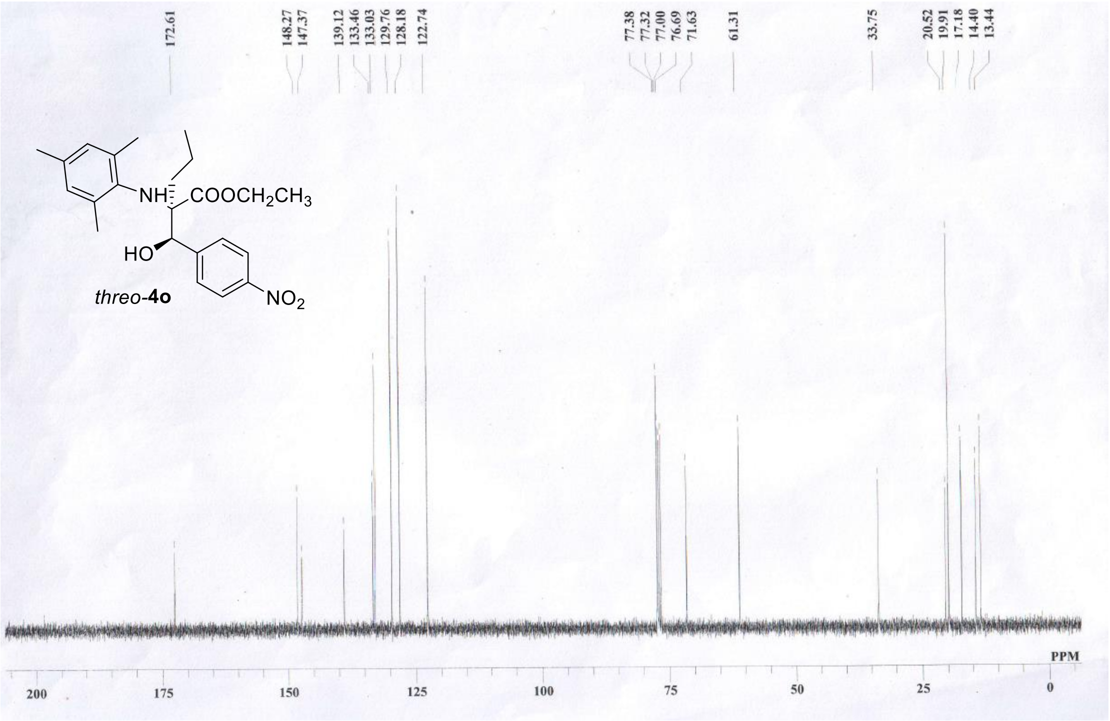
172.61
148.27
147.37
139.12
133.46
133.03
129.76
128.18
122.74
77.38
77.32
77.00
76.69
71.63
61.31
33.75
20.52
19.91
17.18
14.40
13.44
NH
COOCH2CH3
HO
NO2
threo-4o
PPM
200
175
150
125
100
75
50
25
0

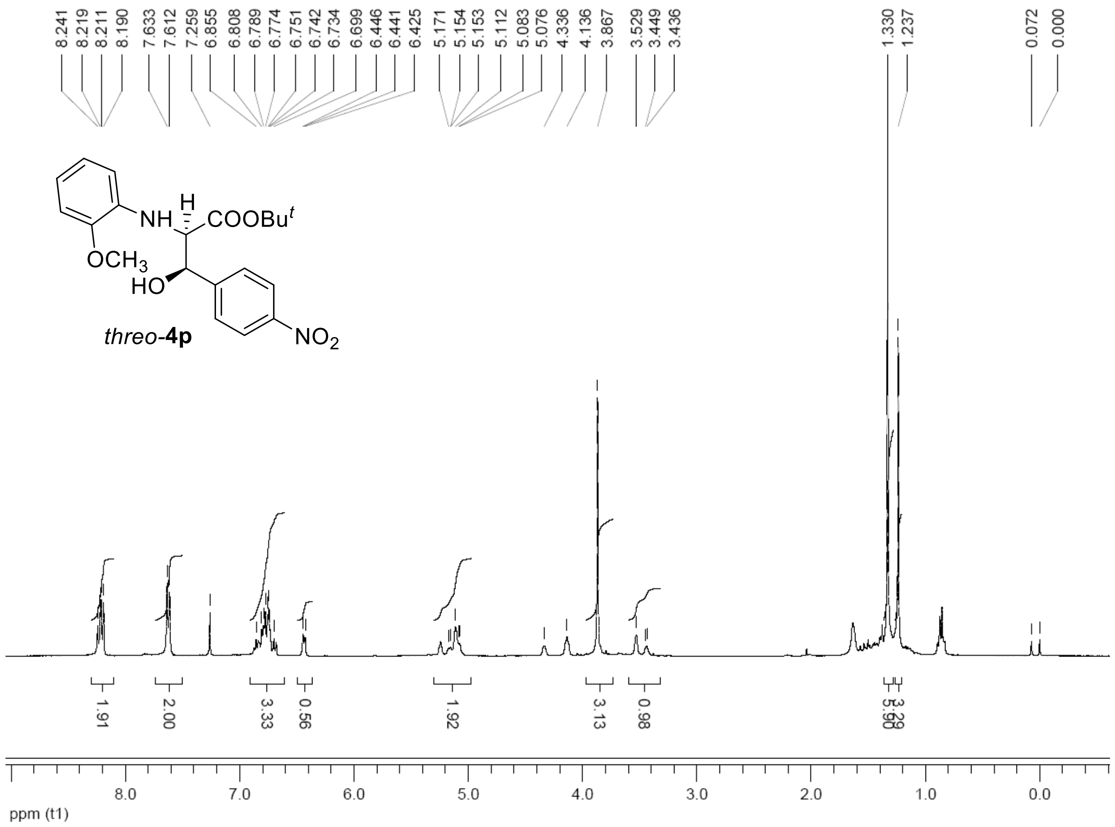
8.241 8.219 8.211 8.190 7.633 7.612 7.259 6.855 6.808 6.789 6.774 6.751 6.742 6.734 6.699 6.446 6.441 6.425 5.171 5.154 5.153 5.112 5.083 5.076 4.336 4.136 3.867 3.529 3.449 3.436 1.330 1.237 0.072 0.000
NH
H
COOBu^t
OCH$_3$
HO
threo-**4p**
NO$_2$
1.91 2.00 3.33 0.56 1.92 3.13 0.98
8.0 7.0 6.0 5.0 4.0 3.0 2.0 1.0 0.0
ppm (t1)

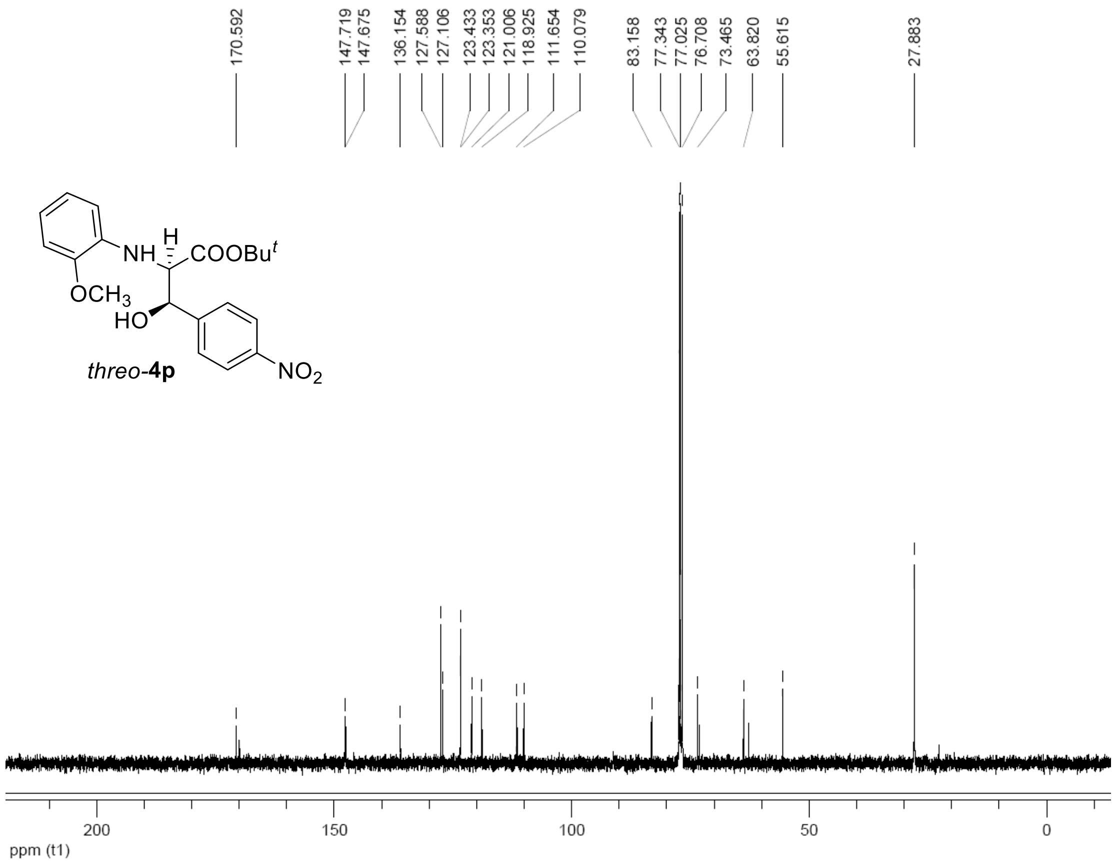
170.592 147.719 147.675 136.154 127.588 127.106 123.433 123.353 121.006 118.925 111.654 110.079 83.158 77.343 77.025 76.708 73.465 63.820 55.615 27.883
NH
H
COOBu^t
OCH$_3$
HO
threo-**4p**
NO$_2$
200 150 100 50 0
ppm (t1)

HPLC spectra of compounds

Chiralpak Column: OD-H
M.P: *n*-Hex/*i*-PrOH=5:1
UV: 254nm
0.8 ml/min
Injection Volume: 20μl

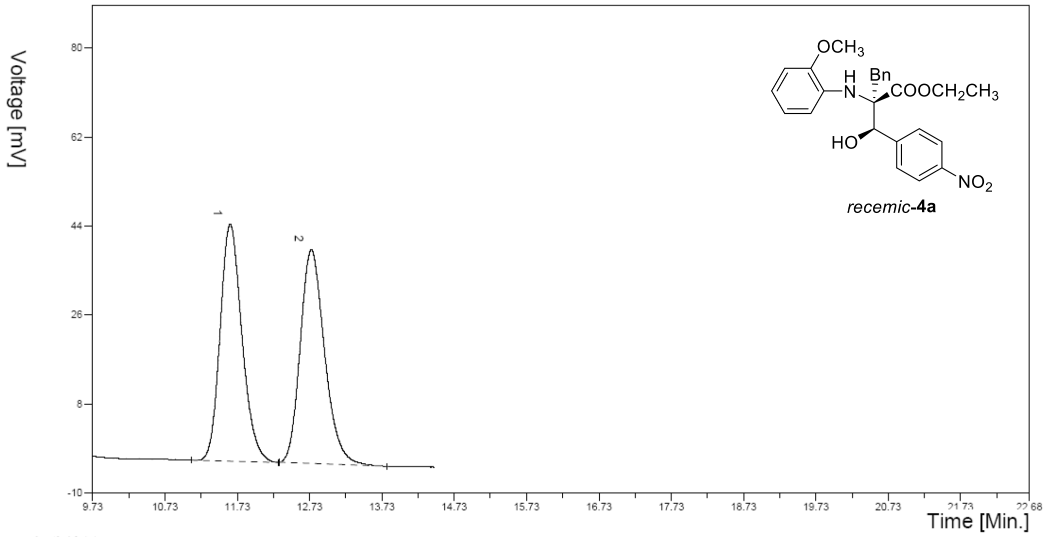

积分结果

#	组分名	保留时间(min)	峰高(mv)	峰面积(mv.sec)	面积百分比(%)
1	Unknown	11.63	47.97	1006.29	49.8596
2	Unknown	12.75	43.21	1011.96	50.1404
合计			91.18	2018.25	100

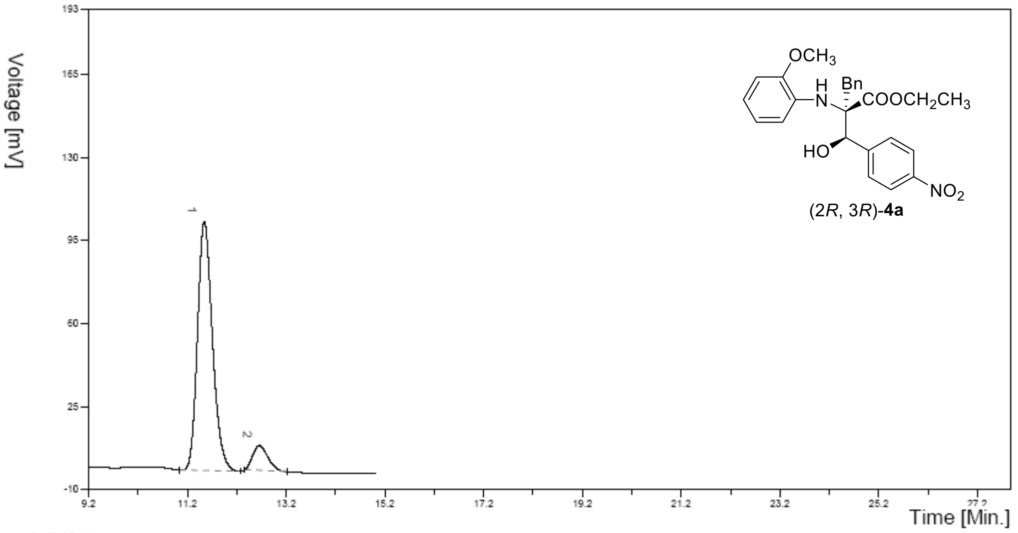

积分结果

#	组分名	保留时间(min)	峰高(mv)	峰面积(mv.sec)	面积百分比(%)
1	Unknown	11.58	104.87	2203.27	90.7988
2	Unknown	12.70	10.50	223.27	9.2012
合计			115.37	2426.54	100

Chiralpak Column: IA
M.P: *n*-Hex/*i*-PrOH=5:1
UV: 254nm
0.8 ml/min
Injection Volume: 20μl

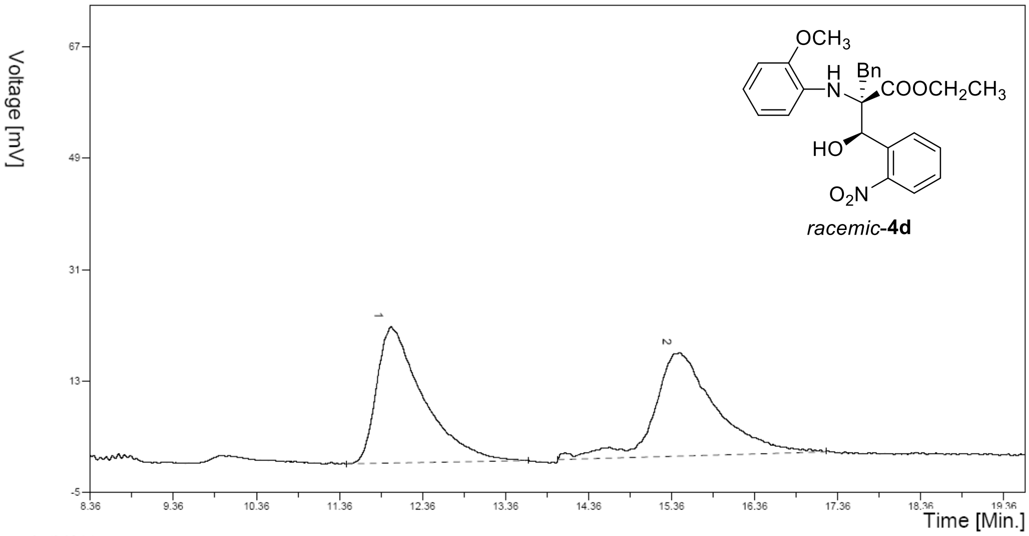

积分结果

#	组分名	保留时间(min)	峰高(mv)	峰面积(mv.sec)	面积百分比(%)
1	Unknown	11.98	22.03	854.77	49.8967
2	Unknown	15.46	16.70	858.31	50.1033
合计			38.73	1713.08	100

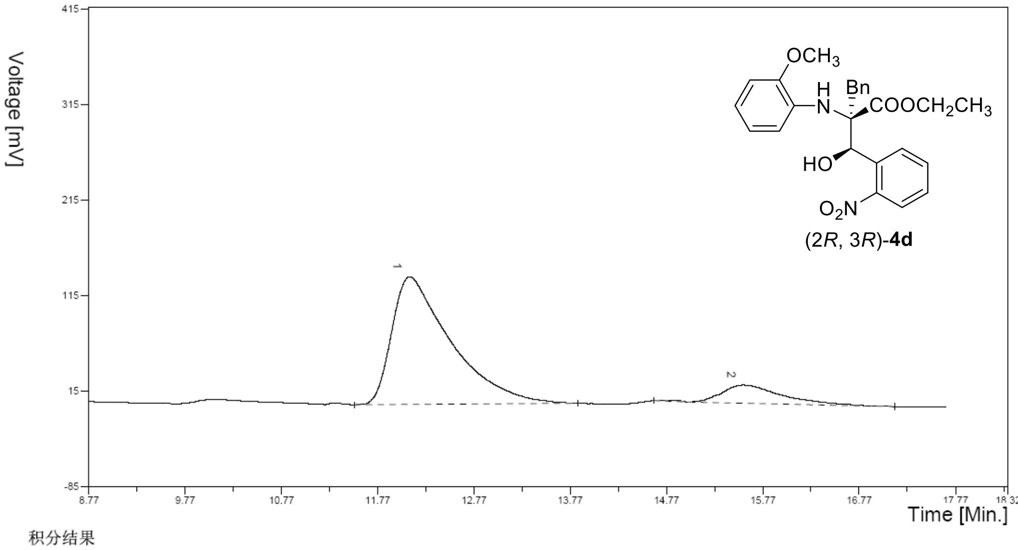

积分结果

#	组分名	保留时间(min)	峰高(mv)	峰面积(mv.sec)	面积百分比(%)
1	Unknown	12.10	133.45	5669.87	87.6914
2	Unknown	15.57	18.68	795.84	12.3086
合计			152.14	6465.71	100

Chiralpak Column: IA
M.P: *n*-Hex/*i*-PrOH=5:1
UV: 254nm
0.8 ml/min
Injection Volume: 20μl

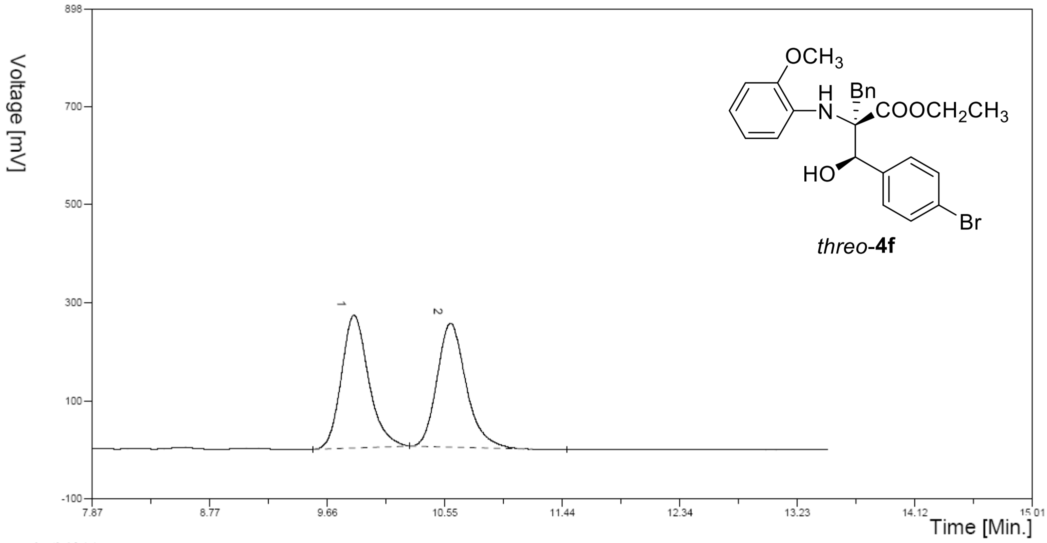

积分结果

#	组分名	保留时间(min)	峰高(mv)	峰面积(mv.sec)	面积百分比(%)
1	Unknown	9.86	270.50	3886.20	50.3582
2	Unknown	10.59	252.13	3830.91	49.6418
合计			522.63	7717.11	100

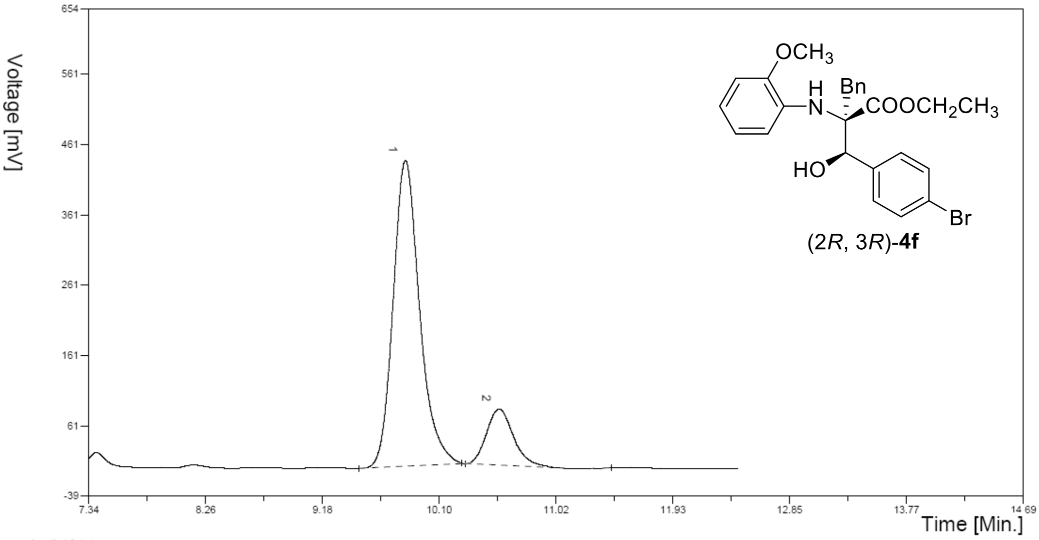

积分结果

#	组分名	保留时间(min)	峰高(mv)	峰面积(mv.sec)	面积百分比(%)
1	Unknown	9.83	434.92	6280.18	85.5787
2	Unknown	10.57	78.47	1058.30	14.4213
合计			513.39	7338.49	100

Chiralpak Column: IA
M.P: *n*-Hex/*i*-PrOH=5:1
UV: 254nm
0.8 ml/min
Injection Volume: 20μl

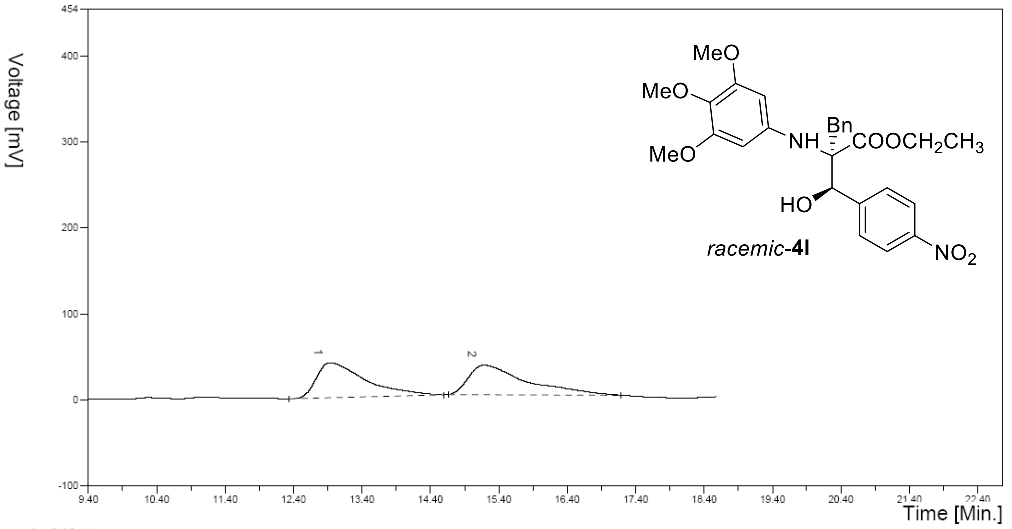

积分结果

#	组分名	保留时间(min)	峰高(mv)	峰面积(mv.sec)	面积百分比(%)
1	Unknown	12.94	40.64	2014.36	49.8618
2	Unknown	15.19	34.26	2025.52	50.1382
合计			74.91	4039.88	100

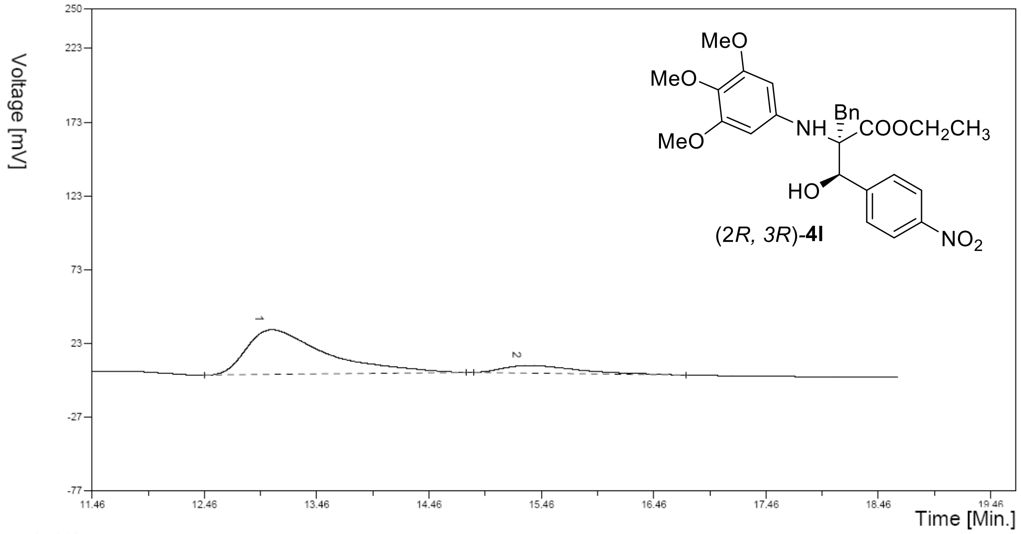

积分结果

#	组分名	保留时间(min)	峰高(mv)	峰面积(mv.sec)	面积百分比(%)
1	Unknown	13.06	30.22	1489.60	86.1703
2	Unknown	15.35	5.22	239.07	13.8297
合计			35.44	1728.67	100

DFT calculation details

The Density Functional Theory (DFT) calculations were carried out by using the quantum chemical calculation software Gaussian09.[1] The geometrical structures of transition states and intermediates are optimized at the level of the M06/Lanl2dz+6-31G* method,[2] which means that the Lanl2dz basis set[3] is used for the description of metal element Rh and the 6-31G* basis is used for the nonmetal elements C, O, N and H. The frequency analyses are performed based on the optimized structures obtained in gas phase to verify the nature of stationary points on the potential surfaces. The solvent effects of CH_2Cl_2 for the reactions are evaluated using the integral equation formalism model (PCM).[4] The intrinsic reaction coordinate (IRC)[5] calculations are conducted to assure that the transition states connect the correct products in the forward directions and reactants in the reverse directions.

References

1. Gaussian09, Revision **B.01**, M. J. Frisch, G. W. Trucks, H. B. Schlegel, G. E. Scuseria, M. A. Robb, J. R. Cheeseman, G. Scalmani, V. Barone, B. Mennucci, G. A. Petersson, H. Nakatsuji, M. Caricato, X. Li, H. P. Hratchian, A. F. Izmaylov, J. Bloino, G. Zheng, J. L. Sonnenberg, M. Hada, M. Ehara, K. Toyota, R. Fukuda, J. Hasegawa, M. Ishida, T. Nakajima, Y. Honda, O. Kitao, H. Nakai, T. Vreven, J. A. Montgomery, Jr., J. E. Peralta, F. Ogliaro, M. Bearpark, J. J. Heyd, E. Brothers, K. N. Kudin, V. N. Staroverov, R. Kobayashi, J. Normand, K. Raghavachari, A. Rendell, J. C. Burant, S. S. Iyengar, J. Tomasi, M. Cossi, N. Rega, J. M. Millam, M. Klene, J. E. Knox, J. B. Cross, V. Bakken, C. Adamo, J. Jaramillo, R. Gomperts, R. E. Stratmann, O. Yazyev, A. J. Austin, R. Cammi, C. Pomelli, J. W. Ochterski, R. L. Martin, K. Morokuma, V. G. Zakrzewski, G. A. Voth, P. Salvador, J. J. Dannenberg, S. Dapprich, A. D. Daniels, Ö. Farkas, J. B. Foresman, J. V. Ortiz, J. Cioslowski, and D. J. Fox, Gaussian, Inc., Wallingford CT, **2009**.
2. Zhao, Y.; Truhlar, D. G. *Theor. Chem. Acc*. **2008**, *120*, 215.
3. a) Hay, P. J.; Wadt, W. R. *J. Chem. Phys.* **1985**, *82*, 299; b) Wadt, W. R.; Hay, P. J. *J. Chem. Phys.* **1985**, *82*, 284.
4. Scalmani, G.; Frisch, M. J. *J. Chem. Phys.* **2010**, *132*, 114110.
5. Fukui, K. *Acc. Chem. Res.* **1981**, *14*, 363.

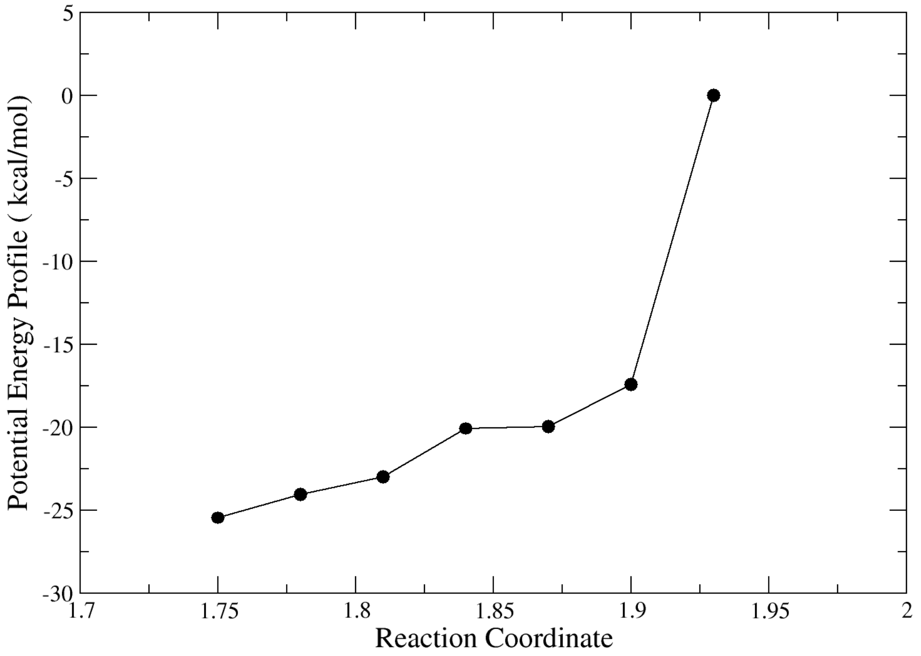

Figure S1 The constrained optimization results indicate that the attack of **A9** by aldehyde is a process that has no energy barrier. The C-C distance between the attached carbon atom in **A9** and the carbon in aldehyde is set as the reaction coordinate, with the discrete values 1.93, 1.90, 1.87, 1.84, 1.81, 1.78 and 1.75 Å adopted in the calculations, respectively. The hydrogen transfer from the NH_2 group of **A9** to the carbonyl oxygen atom of aldehyde takes place spontaneously at the distance 1.90 Å along the reaction coordinate.

Optimized structures and coordinates in Scheme 2

A3: 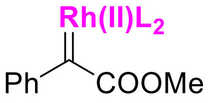

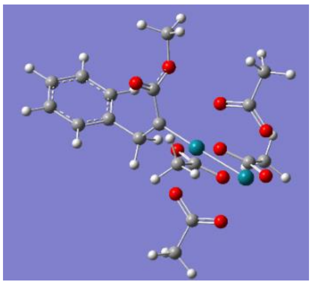

C	1.71563200	0.90276900	-0.36667700
C	1.93300900	2.35669200	-0.27474800
O	1.78211200	3.07321100	-1.23616400
O	2.27578000	2.73891000	0.95314400
Rh	-2.31358000	-0.69159800	0.42559700
Rh	-0.08236100	0.22295100	-0.08462000
C	-0.25566700	-1.40086200	2.34405200
C	-0.61424600	-2.39879400	-1.22261700
C	-1.77584000	1.95610400	1.52036400
C	-2.17355000	0.90147500	-1.99203000
O	0.57491700	-0.73720000	1.63703200
O	0.30129900	-1.50970500	-1.14731200
O	-0.61583900	1.91218700	0.99063400
O	-0.93150600	1.07271300	-1.76090600
O	-1.47943700	-1.56087700	2.11504100
O	-1.75408800	-2.35251800	-0.69891300
O	-2.65863200	1.06277500	1.48606400
O	-2.98773100	0.24222300	-1.29630700
C	0.31399500	-2.02664900	3.58645100
H	-0.38774300	-2.75402000	4.00238100
H	1.27425600	-2.50372200	3.36554400
H	0.49929900	-1.24186200	4.32980800
C	-2.09651000	3.23530100	2.24319800
H	-2.22279400	4.03911800	1.50767500
H	-3.01733000	3.12948200	2.82203100
H	-1.26340300	3.51543400	2.89666900
C	-2.68373900	1.56102100	-3.24219200
H	-2.40329200	2.61972400	-3.24239500
H	-2.20639200	1.09964000	-4.11449600
H	-3.76862100	1.45404900	-3.31729700
C	-0.26268100	-3.60949000	-2.04144800
H	-1.03997600	-4.37191200	-1.94955500
H	-0.15934800	-3.31760500	-3.09319800

H	0.70310600	-4.01211500	-1.71774800
C	2.31814300	4.15291300	1.14950400
H	3.02885400	4.61657900	0.45787300
H	1.32122000	4.57863500	0.98708200
H	2.63361200	4.30081400	2.18345200
C	2.86808300	0.00314700	-0.54905500
H	2.93089400	-0.50135300	0.43901000
H	2.57002200	-0.81286900	-1.22855500
C	4.17286700	0.64842500	-0.92668000
C	5.07180400	1.07293700	0.05220800
C	4.47351600	0.87169300	-2.27070800
C	6.26023000	1.69968200	-0.30750500
H	4.83135300	0.91258500	1.10427000
C	5.66052200	1.49888200	-2.63174200
H	3.76577400	0.55210100	-3.03654200
C	6.55713600	1.91184700	-1.65040000
H	6.95821200	2.02130700	0.46385000
H	5.88532000	1.66815400	-3.68341100
H	7.48783100	2.40112300	-1.93267100

A5: 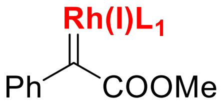

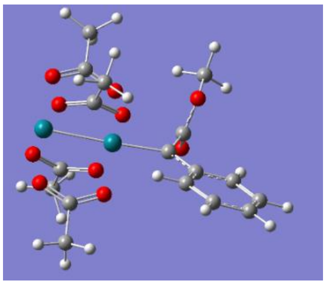

C	1.87583500	0.62440000	-0.34420800
C	2.02640400	2.09303600	-0.41983100
O	1.84828500	2.74113600	-1.42477100
O	2.35681900	2.59194900	0.77542600
Rh	-2.33403400	-0.61750300	0.44653600
Rh	-0.00243600	0.03997300	-0.02562700
C	-0.40064700	-1.50785400	2.42228300
C	-0.82808800	-2.50828900	-1.17216800
C	-1.55639100	1.96103400	1.52957800
C	-1.97626000	0.90818200	-1.98567200
O	0.50569300	-0.91069700	1.75229200
O	0.19824100	-1.76263100	-1.03471500
O	-0.41760400	1.80988500	0.97753700
O	-0.72278100	0.88598300	-1.76364800

O	-1.62193200	-1.58694700	2.13865500
O	-1.97327200	-2.31464800	-0.69285000
O	-2.51097300	1.14424900	1.53166600
O	-2.88045100	0.40546500	-1.26886100
C	0.06278400	-2.15859100	3.69635900
H	-0.68046500	-2.87766400	4.05040300
H	1.03122000	-2.64595900	3.54607100
H	0.19874400	-1.38444600	4.46143000
C	-1.75598600	3.27670100	2.23104600
H	-1.85510700	4.07018900	1.47984900
H	-2.66016300	3.25207900	2.84463800
H	-0.88160600	3.51179100	2.84757300
C	-2.38564500	1.61578900	-3.24689800
H	-2.04244600	2.65564500	-3.20905400
H	-1.89257300	1.14554900	-4.10502900
H	-3.47053100	1.58153700	-3.37330500
C	-0.62636800	-3.73050300	-2.02476400
H	-1.42262000	-4.45788000	-1.84686900
H	-0.64832400	-3.43273000	-3.08032400
H	0.35425800	-4.17594500	-1.82983000
C	2.23503400	4.00772100	0.88865100
H	2.91978100	4.51615900	0.20149900
H	1.20556200	4.30837400	0.66170700
C	3.05159500	-0.17008900	-0.47628800
C	3.04040200	-1.54428500	-0.14203200
C	4.26372400	0.40973400	-0.92827900
C	4.19869600	-2.29556800	-0.24397000
H	2.11464300	-1.98361400	0.21524300
C	5.40613500	-0.35571600	-1.05623500
H	4.27970900	1.46377100	-1.20258500
C	5.37483400	-1.70738200	-0.70660500
H	4.18941300	-3.34801100	0.03185800
H	6.32828300	0.09312800	-1.41943900
H	6.28010500	-2.30648800	-0.79288700
H	2.48415900	4.24716300	1.92406200

A5*(Conformer of A5): 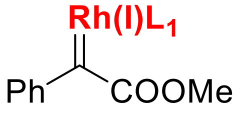

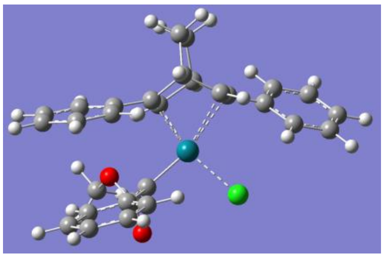

C	0.37810400	1.32214400	1.14330400
C	1.75806600	1.86201800	1.09530900
O	1.97817900	3.03942000	0.94102800
O	2.71934900	0.92968900	1.24550000
C	4.04977100	1.44176000	1.16176400
H	4.22726000	1.88687700	0.17707600
H	4.21929600	2.20549800	1.92825800
Rh	-0.30027700	0.47691300	-0.40215400
C	0.82737700	-3.11267900	-2.00636800
C	1.02363200	-1.57735800	-1.94601500
C	-1.04525200	-2.21875200	-0.57540900
C	-0.41882800	-3.49780700	-1.18713100
H	0.72409200	-3.42225100	-3.05615400
H	1.72779600	-3.60347900	-1.61273400
H	-1.16797300	-3.99878800	-1.81402100
H	-0.16281700	-4.18886800	-0.37152200
C	-0.24328900	-0.91988200	-2.46736500
C	-1.32880800	-1.23364900	-1.68983300
H	-2.34887900	-0.96819600	-1.96137500
H	-1.94951200	-2.45544200	-0.00488900
H	1.91212200	-1.25895700	-2.49992600
C	0.02191700	-1.56089400	0.28873900
C	1.12061700	-1.15956000	-0.49486600
H	2.08794700	-0.91540900	-0.05998100
Cl	-1.77752800	2.25008800	-1.10905400
C	0.05820700	-1.75987800	1.75081400
C	-1.12198800	-1.74514700	2.50364200
C	1.27348100	-1.92431900	2.42624400
C	-1.08893100	-1.87741700	3.88538600
H	-2.07756000	-1.58623800	2.00288000
C	1.30711500	-2.06247100	3.80901700
H	2.20205100	-1.94515100	1.85732700
C	0.12664200	-2.03696600	4.54481000
H	-2.01854000	-1.84243400	4.45159900
H	2.26339800	-2.19580000	4.31299800
H	0.15288900	-2.14288200	5.62816800
C	-0.29095100	-0.23359600	-3.76678800
C	0.70453700	-0.46562200	-4.72496600

C	-1.36168300	0.60511300	-4.10992000
C	0.63265600	0.11504900	-5.98580500
H	1.53943200	-1.12776500	-4.49985500
C	-1.42499100	1.19192600	-5.36482300
H	-2.12006300	0.84052800	-3.36581900
C	-0.43123100	0.94843100	-6.30997600
H	1.41438100	-0.08565100	-6.71672200
H	-2.25514700	1.85447800	-5.60301500
H	-0.48563200	1.41113800	-7.29403000
H	4.71007500	0.58716200	1.32185200
C	-0.34211700	1.47746200	2.38625600
C	0.34926100	1.51088900	3.61390400
C	-1.74876900	1.56458400	2.39411800
C	-0.34465500	1.59465500	4.80938500
H	1.43605900	1.42906100	3.62222800
C	-2.43364400	1.69126800	3.59266500
H	-2.27113800	1.57819000	1.43775000
C	-1.73524700	1.69499400	4.79882000
H	0.19667600	1.59499300	5.75370800
H	-3.51761200	1.78754300	3.58890800
H	-2.27717700	1.78257700	5.73953300

A4:

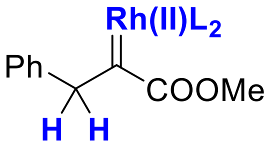

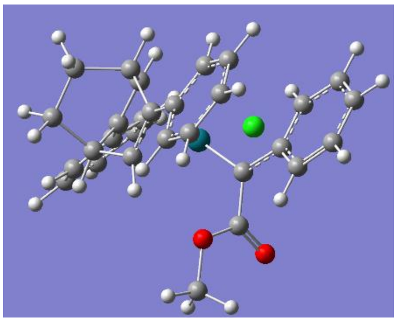

C	0.37810400	1.32214400	1.14330400
C	1.75806600	1.86201800	1.09530900
O	1.97817900	3.03942000	0.94102800
O	2.71934900	0.92968900	1.24550000
C	4.04977100	1.44176000	1.16176400
H	4.22726000	1.88687700	0.17707600
H	4.21929600	2.20549800	1.92825800
Rh	-0.30027700	0.47691300	-0.40215400
C	0.82737700	-3.11267900	-2.00636800
C	1.02363200	-1.57735800	-1.94601500

C	-1.04525200	-2.21875200	-0.57540900
C	-0.41882800	-3.49780700	-1.18713100
H	0.72409200	-3.42225100	-3.05615400
H	1.72779600	-3.60347900	-1.61273400
H	-1.16797300	-3.99878800	-1.81402100
H	-0.16281700	-4.18886800	-0.37152200
C	-0.24328900	-0.91988200	-2.46736500
C	-1.32880800	-1.23364900	-1.68983300
H	-2.34887900	-0.96819600	-1.96137500
H	-1.94951200	-2.45544200	-0.00488900
H	1.91212200	-1.25895700	-2.49992600
C	0.02191700	-1.56089400	0.28873900
C	1.12061700	-1.15956000	-0.49486600
H	2.08794700	-0.91540900	-0.05998100
Cl	-1.77752800	2.25008800	-1.10905400
C	0.05820700	-1.75987800	1.75081400
C	-1.12198800	-1.74514700	2.50364200
C	1.27348100	-1.92431900	2.42624400
C	-1.08893100	-1.87741700	3.88538600
H	-2.07756000	-1.58623800	2.00288000
C	1.30711500	-2.06247100	3.80901700
H	2.20205100	-1.94515100	1.85732700
C	0.12664200	-2.03696600	4.54481000
H	-2.01854000	-1.84243400	4.45159900
H	2.26339800	-2.19580000	4.31299800
H	0.15288900	-2.14288200	5.62816800
C	-0.29095100	-0.23359600	-3.76678800
C	0.70453700	-0.46562200	-4.72496600
C	-1.36168300	0.60511300	-4.10992000
C	0.63265600	0.11504900	-5.98580500
H	1.53943200	-1.12776500	-4.49985500
C	-1.42499100	1.19192600	-5.36482300
H	-2.12006300	0.84052800	-3.36581900
C	-0.43123100	0.94843100	-6.30997600
H	1.41438100	-0.08565100	-6.71672200
H	-2.25514700	1.85447800	-5.60301500
H	-0.48563200	1.41113800	-7.29403000
H	4.71007500	0.58716200	1.32185200
C	-0.34211700	1.47746200	2.38625600
C	0.34926100	1.51088900	3.61390400
C	-1.74876900	1.56458400	2.39411800
C	-0.34465500	1.59465500	4.80938500
H	1.43605900	1.42906100	3.62222800
C	-2.43364400	1.69126800	3.59266500

H	-2.27113800	1.57819000	1.43775000
C	-1.73524700	1.69499400	4.79882000
H	0.19667600	1.59499300	5.75370800
H	-3.51761200	1.78754300	3.58890800
H	-2.27717700	1.78257700	5.73953300

TS-1-a:

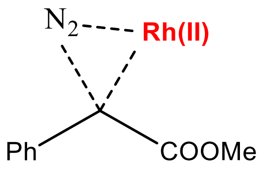

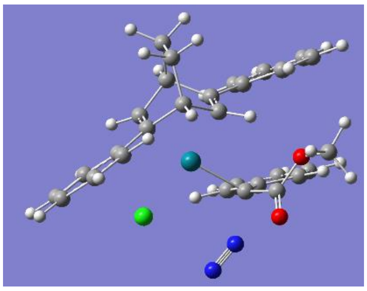

C	1.03754500	-1.33989000	0.79041100
C	1.04820900	-0.97937200	2.24414900
O	0.41840700	-1.53975700	3.11067200
O	1.79836400	0.11494400	2.47443300
C	1.73385400	0.61259000	3.80748500
H	0.70002000	0.85687800	4.07996800
H	2.11423100	-0.12894500	4.51781800
Rh	-0.53408700	-0.36059200	-0.17299000
C	-1.94601700	3.38196100	0.11774300
C	-1.85458100	1.95556800	0.70433300
C	-0.56336300	2.04925000	-1.51425100
C	-1.14892700	3.44536900	-1.19913600
H	-3.00500700	3.62987500	-0.04485800
H	-1.55641000	4.10045700	0.85188400
H	-1.78521200	3.76567100	-2.03475900
H	-0.32015000	4.16457500	-1.12868900
C	-2.40530800	0.97531600	-0.31860000
C	-1.65998200	1.00729800	-1.49570000
H	-1.99341900	0.53289600	-2.41657000
H	-0.02166300	2.05121700	-2.46631300
H	-2.36277400	1.88346000	1.66949000
C	0.33324400	1.64005400	-0.35355500
C	-0.40645200	1.54163800	0.84013400
H	0.07258400	1.54391600	1.81748600
Cl	-1.43250500	-2.34716400	-1.25566600
C	1.79007800	1.84553200	-0.44539700

C	2.49481300	1.45356000	-1.59003500
C	2.49435500	2.47340700	0.58761200
C	3.86315900	1.66594900	-1.68968400
H	1.96513700	0.94557200	-2.39681800
C	3.86369200	2.69025500	0.48786600
H	1.95362800	2.80744400	1.47288000
C	4.55304000	2.28629000	-0.65119400
H	4.39629800	1.33610200	-2.58017700
H	4.39230900	3.18862200	1.29949800
H	5.62559200	2.45617700	-0.73188000
C	-3.73324000	0.35384300	-0.16870200
C	-4.56073800	0.67026500	0.91707600
C	-4.23629200	-0.52304900	-1.14242400
C	-5.83626600	0.12891600	1.03112000
H	-4.22049900	1.35941600	1.68724100
C	-5.50536800	-1.06624900	-1.02608600
H	-3.60616400	-0.81732700	-1.97779300
C	-6.31395500	-0.74383800	0.06188800
H	-6.45674200	0.39385400	1.88576100
H	-5.86410400	-1.75578500	-1.78818900
H	-7.30983100	-1.17436000	0.15323300
H	2.35811700	1.50982500	3.81680600
C	2.29840900	-1.50198100	0.01400600
C	3.56826300	-1.23877300	0.54372000
C	2.20413600	-1.97722400	-1.30500100
C	4.70641000	-1.42122500	-0.23321500
H	3.66885100	-0.85597700	1.55528000
C	3.34577200	-2.18955200	-2.06396100
H	1.21684700	-2.19505500	-1.71574300
C	4.60181100	-1.90558400	-1.53232300
H	5.68383900	-1.19113500	0.18800700
H	3.25288000	-2.57477400	-3.07795900
H	5.49799600	-2.06416000	-2.13038900
N	0.54373100	-2.98523500	0.99944700
N	-0.01376100	-3.87773500	0.63282200

TS-1-b:

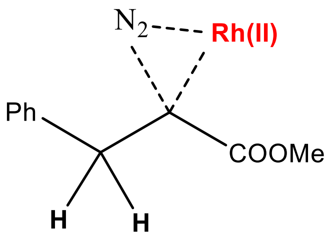

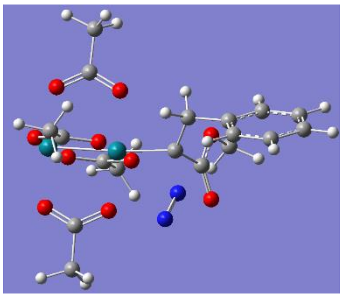

C	1.64371700	0.11920700	-0.19110500
C	2.06441100	1.41991200	-0.78738300
O	2.20147200	1.66225400	-1.96102800
O	2.16056400	2.31753300	0.20289800
Rh	-2.82498000	-0.20495900	0.29787200
Rh	-0.40168400	-0.04281000	-0.04175000
C	-1.30781600	0.38550500	2.70011200
C	-1.34623400	-2.70483900	0.60894700
C	-1.86804400	2.45317500	-0.37143800
C	-1.94231500	-0.63954300	-2.43521100
O	-0.24803900	0.34439000	1.99327300
O	-0.26949000	-2.07491500	0.33650700
O	-0.68746300	1.97624900	-0.41719800
O	-0.74677700	-0.45167900	-2.03367000
O	-2.48743700	0.20886500	2.30168800
O	-2.50567800	-2.22330600	0.67072900
O	-2.92848200	1.82862900	-0.10877500
O	-2.98424400	-0.60760100	-1.73127200
C	-1.09793500	0.68367500	4.15891000
H	-2.04501100	0.63646400	4.70179300
H	-0.38506000	-0.03198000	4.58337300
H	-0.65929100	1.68241400	4.26752600
C	-1.97607100	3.92581900	-0.65825500
H	-1.51783100	4.14616100	-1.62918700
H	-3.02121700	4.24438000	-0.65577100
H	-1.41844600	4.48557400	0.10227700
C	-2.09487200	-0.90201300	-3.90670000
H	-2.01392300	0.04844500	-4.44814600
H	-1.28782000	-1.55223100	-4.25965600
H	-3.07062600	-1.34750400	-4.11671300
C	-1.18450900	-4.17705200	0.86854100
H	-2.13272200	-4.61775200	1.18567700
H	-0.83831200	-4.67159700	-0.04668200
H	-0.41890200	-4.33790700	1.63545200
C	2.27077500	3.66978300	-0.23637200
H	3.22868500	3.82952900	-0.74378500
H	1.45426200	3.90603000	-0.92657000

H	2.20701500	4.28239300	0.66468500
C	2.46326900	-0.50776300	0.89001700
H	2.21334200	0.12303100	1.76082600
H	2.04194500	-1.49854100	1.11250600
C	3.94973200	-0.57030500	0.65752000
C	4.76829600	0.53941800	0.88173800
C	4.52854500	-1.75026000	0.18606700
C	6.13389100	0.47126200	0.63004100
H	4.32457000	1.46144100	1.25754700
C	5.89418600	-1.81889700	-0.06931100
H	3.89955300	-2.62797300	0.02656700
C	6.70006100	-0.70665700	0.15084700
H	6.76094000	1.34209900	0.81459400
H	6.33006400	-2.74683900	-0.43605800
H	7.76954700	-0.75899100	-0.04541400
N	2.01463900	-0.95473400	-1.63990000
N	1.72112500	-1.62328000	-2.47369300

TS-2-a:

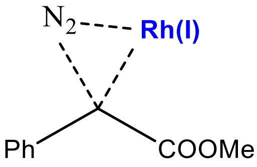

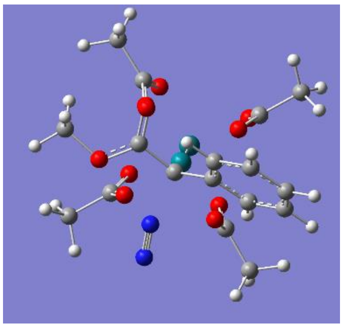

C	1.93053400	0.74922800	0.34166400
C	2.01043900	2.13534100	-0.20074000
O	2.37955900	2.34199300	-1.33393600
O	1.54290400	3.07666600	0.62439300
Rh	-2.36840400	-0.70178600	-0.39913300
Rh	-0.09108700	0.06306400	0.08267400
C	-1.01551700	-2.19313300	1.69344500
C	-0.31137300	-1.97272000	-1.98747700
C	-2.17376400	1.32850900	1.67096100
C	-1.51208800	1.56623600	-1.97140600
O	0.01956700	-1.48070300	1.47701800
O	0.53546800	-1.20976200	-1.42379300
O	-0.90682700	1.25926200	1.56694900

O	-0.43975900	1.57417600	-1.28875400
O	-2.12837600	-2.09551600	1.11679700
O	-1.54936000	-2.03326700	-1.75625100
O	-3.02258900	0.69435000	0.99034600
O	-2.45674400	0.73742200	-1.88144600
C	-0.86180100	-3.27446800	2.72653200
H	-1.84126000	-3.61391700	3.07324600
H	-0.33722700	-4.12574300	2.27484900
H	-0.25787200	-2.91833300	3.56684600
C	-2.69125200	2.25335900	2.73773000
H	-2.12750300	3.19171200	2.73036200
H	-3.75793300	2.44403900	2.59470400
H	-2.54257100	1.78691400	3.71934500
C	-1.66412000	2.68597900	-2.96261200
H	-2.16678500	3.52877500	-2.47101900
H	-0.68355200	3.02375400	-3.31159900
H	-2.28619900	2.36608900	-3.80333300
C	0.24299100	-2.88013300	-3.05008800
H	1.17937100	-3.33095800	-2.70553900
H	-0.48346500	-3.65251700	-3.31475900
H	0.47562000	-2.28475900	-3.94124500
C	1.36570300	4.35531100	0.02183500
H	2.31599900	4.73885100	-0.36482300
H	0.64622700	4.28178400	-0.80129300
C	3.08780600	-0.14239300	0.15071400
C	2.99036200	-1.48585900	0.54657300
C	4.29334600	0.31449200	-0.40740000
C	4.06825300	-2.34432300	0.39090600
H	2.05735500	-1.84035600	0.97963100
C	5.36057000	-0.55499200	-0.58007200
H	4.37899500	1.34663400	-0.73552500
C	5.25294200	-1.88329400	-0.17754400
H	3.98168000	-3.38256200	0.70640700
H	6.28508900	-0.19111200	-1.02457600
H	6.09471400	-2.56173800	-0.30771000
H	0.98247200	5.00496100	0.81075000
N	1.57821800	0.79220500	3.14465000
N	1.90228600	1.00504200	2.10362200

Optimized structures and coordinates in Scheme 3

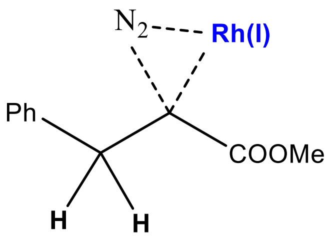

TS-2-b:

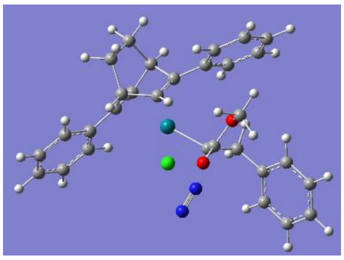

C	1.18303800	-0.73033600	0.15194200
C	1.65121400	-0.24742500	1.49230900
O	1.44244300	-0.76105900	2.56605800
O	2.30217100	0.91244900	1.32683300
C	2.80687100	1.49501500	2.52495400
H	1.99728000	1.68184700	3.24004700
H	3.54092300	0.82485400	2.98731300
Rh	-0.70901300	-0.16304700	-0.38542600
C	-3.16503600	2.74194400	0.87499900
C	-2.53359900	1.35450100	1.13009400
C	-1.79532300	2.22402800	-1.17768900
C	-2.71132100	3.27022300	-0.50019800
H	-4.26012600	2.65466600	0.92389300
H	-2.86119900	3.42564400	1.68022800
H	-3.57131700	3.47378000	-1.15178400
H	-2.15223200	4.21211500	-0.39894200
C	-2.92837100	0.41903800	-0.00290500
C	-2.49532100	0.88313200	-1.23674000
H	-2.83592900	0.45705500	-2.17888100
H	-1.46162100	2.55813100	-2.16705700
H	-2.81018200	0.95136200	2.10918400
C	-0.61209200	1.97422600	-0.24861000
C	-1.02843100	1.44984200	0.99271600
H	-0.39284700	1.48029400	1.88010400
Cl	-0.80895400	-2.12832100	-1.85682500
C	0.65092600	2.70199700	-0.47577700
C	1.38742500	2.51947600	-1.65162300
C	1.11806600	3.61254000	0.47525000
C	2.59510600	3.18041600	-1.83822300
H	1.01785800	1.82044000	-2.40491800
C	2.31642200	4.29186100	0.27999400

H	0.52871300	3.78324100	1.37780000
C	3.06748800	4.06328000	-0.86857100
H	3.17001300	3.00952100	-2.74710500
H	2.66579300	5.00214300	1.02889100
H	4.01187600	4.58390100	-1.01840700
C	-3.89818100	-0.67146300	0.19402800
C	-4.74324200	-0.67506200	1.31181200
C	-4.04956100	-1.69059000	-0.75821300
C	-5.71041600	-1.66058700	1.47358600
H	-4.66558900	0.11302600	2.05988800
C	-5.00963800	-2.67692700	-0.58942700
H	-3.36908600	-1.73756800	-1.60705500
C	-5.84566400	-2.66726500	0.52483600
H	-6.35902300	-1.63966900	2.34796600
H	-5.09880300	-3.46754800	-1.33252900
H	-6.59690400	-3.44477900	0.65429500
H	3.27202300	2.43439800	2.21846600
C	2.23307900	-0.86635200	-0.92756700
H	1.76169300	-1.33901700	-1.79978300
H	2.43866200	0.17984900	-1.20777800
C	3.50210300	-1.57477200	-0.53318400
C	3.62263200	-2.94763000	-0.76199100
C	4.55859000	-0.89703200	0.08122200
C	4.76766700	-3.63346900	-0.37118700
H	2.80853400	-3.47546000	-1.26350500
C	5.70483100	-1.58162300	0.47053100
H	4.47583000	0.17865200	0.24349100
C	5.80991900	-2.95198800	0.24973800
H	4.84875400	-4.70302200	-0.55822700
H	6.52344900	-1.04187200	0.94459400
H	6.70775000	-3.48751200	0.55393400
N	0.90087200	-2.40615300	0.65668700
N	0.47941200	-3.36944100	1.00238800

TS-4:

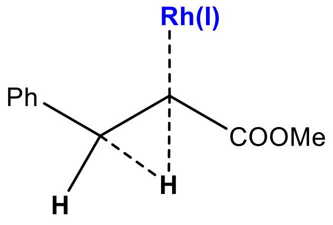

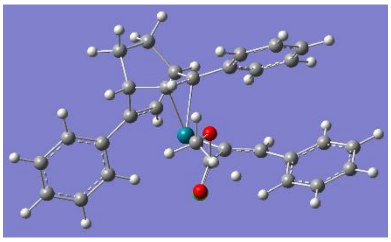

C	1.11600600	-0.86652700	-0.04048500
C	1.14634600	-1.79485400	1.11188800
O	0.87241800	-2.97235700	1.07094600
O	1.50647600	-1.14094200	2.23092200
C	1.56287500	-1.95866500	3.39660400
H	0.59732800	-2.44227300	3.57847300
H	2.32948100	-2.73432200	3.28101100
Rh	-0.73324800	-0.08506000	-0.41319200
C	-2.97093000	2.60406000	1.52775200
C	-2.44905000	1.15587900	1.42040300
C	-1.58004500	2.52359800	-0.57556400
C	-2.43671200	3.42626700	0.33931800
H	-4.07037100	2.59310200	1.53838200
H	-2.64417300	3.03328100	2.48482700
H	-3.26051200	3.85058200	-0.24987900
H	-1.82046300	4.26894900	0.68335600
C	-2.87631700	0.56609400	0.08114300
C	-2.37294400	1.29764400	-0.98738300
H	-2.71887700	1.15673700	-2.01054700
H	-1.21688000	3.08108100	-1.44509900
H	-2.78356800	0.53693400	2.25984300
C	-0.44030300	1.92433500	0.24186800
C	-0.93683100	1.14971100	1.31638800
H	-0.32611900	0.86638700	2.17309700
Cl	-0.75593500	-1.61531000	-2.35254700
C	0.94896100	2.39858200	0.09600100
C	1.43765700	2.77332300	-1.16376700
C	1.83173100	2.43359400	1.18238500
C	2.75832800	3.17118600	-1.33153400
H	0.77952800	2.72217200	-2.03127900
C	3.15573400	2.82138300	1.01401300
H	1.47403200	2.15929100	2.17369100
C	3.62545100	3.19692800	-0.24142800
H	3.11201300	3.46228600	-2.31987900
H	3.82321700	2.83966600	1.87409500
H	4.66071900	3.50942100	-0.36927500
C	-3.93088200	-0.45571700	-0.02104700
C	-4.85791300	-0.61949800	1.01587600

C	-4.06807900	-1.24623000	-1.17120700
C	-5.89570400	-1.53810700	0.90640300
H	-4.78688700	-0.00695600	1.91426000
C	-5.09912300	-2.16844600	-1.27323500
H	-3.32473300	-1.17005200	-1.96422800
C	-6.01930400	-2.31767700	-0.23798800
H	-6.61061400	-1.64380000	1.72102900
H	-5.17555700	-2.78596900	-2.16651500
H	-6.82536500	-3.04485800	-0.32200400
H	1.82175400	-1.29080400	4.22035900
C	2.25885600	-0.65191400	-0.80006600
H	1.19406800	-1.49484400	-1.17004600
H	2.13758400	0.04008200	-1.64194600
C	3.63474200	-1.11253800	-0.57405900
C	3.96859900	-2.28022500	0.12464800
C	4.65757800	-0.29948900	-1.07769800
C	5.30235200	-2.60575300	0.32978200
H	3.18885300	-2.95389600	0.47641400
C	5.98916000	-0.61835800	-0.84973600
H	4.39176100	0.60493300	-1.62624700
C	6.31341300	-1.77385100	-0.14535300
H	5.55513500	-3.52115400	0.86160300
H	6.77525400	0.03059400	-1.23119400
H	7.35679400	-2.03346000	0.02526600

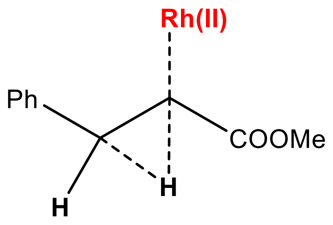

TS-3:

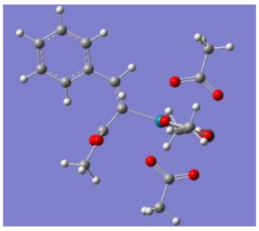

C	1.72625900	0.28026600	-0.07823800
C	2.10123400	1.68852500	-0.31195800
O	2.22240700	2.16498200	-1.41681300
O	2.19074700	2.36797000	0.83698900
Rh	-2.75115700	-0.37842700	0.01319400
Rh	-0.33057400	-0.01402300	-0.02820700
C	-1.51339700	0.06568400	2.62081800

C	-1.12561700	-2.79292900	0.26404200
C	-1.92993500	2.39681900	-0.26955600
C	-1.55211700	-0.45887300	-2.62637500
O	-0.38388100	0.16253100	2.04058100
O	-0.08115700	-2.06319200	0.18958000
O	-0.71814800	2.01028700	-0.21709400
O	-0.42580600	-0.23482300	-2.07936600
O	-2.62990200	-0.12722000	2.07322900
O	-2.32004200	-2.40429300	0.23611200
O	-2.95912000	1.67420500	-0.21460800
O	-2.66466600	-0.58677400	-2.04818300
C	-1.49313000	0.16438500	4.12182900
H	-2.44822500	0.55083900	4.48860200
H	-1.34880400	-0.83881000	4.54231800
H	-0.66546400	0.79628400	4.45665200
C	-2.12271300	3.88252200	-0.40779000
H	-1.58491500	4.23995600	-1.29335100
H	-3.18387000	4.13063700	-0.48722700
H	-1.68970900	4.38797600	0.46387200
C	-1.53256400	-0.56045900	-4.12644600
H	-1.37189100	0.43849800	-4.54951500
H	-0.69597500	-1.18881000	-4.44879900
H	-2.47938200	-0.96045500	-4.49821300
C	-0.87491800	-4.27181300	0.38093100
H	-1.80190800	-4.80183200	0.61291100
H	-0.47378700	-4.64530800	-0.56907100
H	-0.12330900	-4.46659600	1.15349300
C	2.26998600	3.78399000	0.68430500
H	3.15932600	4.06565300	0.11043800
H	1.37497300	4.14531000	0.16554900
H	2.32119300	4.19114500	1.69539400
C	2.64568000	-0.75506700	0.09921000
H	2.06718300	-0.14144800	1.11394300
H	2.18404200	-1.74054500	0.25379000
C	4.11450000	-0.74002100	0.01323400
C	4.82395700	0.25781800	-0.66687100
C	4.81388700	-1.79338600	0.61482500
C	6.21017800	0.20282200	-0.72337000
H	4.29046400	1.05410300	-1.18206100
C	6.19971400	-1.83881100	0.56400700
H	4.25711900	-2.57763700	1.12843200
C	6.89883600	-0.83697800	-0.10400500
H	6.75670200	0.97414600	-1.26243000
H	6.73519400	-2.65747700	1.04066600

H	7.98574200	-0.87307200	-0.15194000

A7:

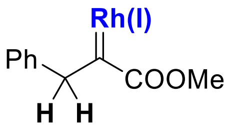

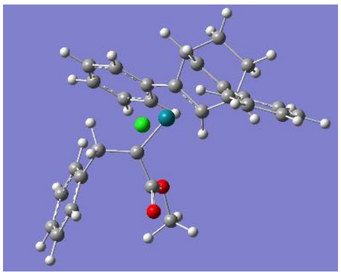

C	0.31467500	1.40953000	1.03224500
C	1.66497200	2.00558500	0.90260200
O	1.85475300	3.12213900	0.48468300
O	2.61640700	1.17682600	1.35875300
C	3.90406900	1.77972600	1.49503200
H	4.27727000	2.12328000	0.52476500
H	3.84481000	2.63481700	2.17903700
Rh	-0.36035900	0.40872300	-0.38232400
C	0.93434800	-3.12273100	-1.99112700
C	1.05070300	-1.57918000	-1.94168000
C	-0.98244400	-2.31575700	-0.56511700
C	-0.28211400	-3.56606200	-1.15657700
H	0.83703000	-3.44510900	-3.03761000
H	1.86304600	-3.56337000	-1.60370900
H	-1.00200000	-4.12550900	-1.76785900
H	0.02054700	-4.22219200	-0.32777600
C	-0.25232400	-0.99134500	-2.45964700
C	-1.32181500	-1.36491400	-1.69190700
H	-2.35313300	-1.14587100	-1.96227100
H	-1.86880000	-2.59530300	0.01404800
H	1.91626400	-1.21682600	-2.50538500
C	0.05235600	-1.59470300	0.29435200
C	1.13520700	-1.15154800	-0.49088600
H	2.09394700	-0.86732100	-0.05783300
Cl	-1.96263900	2.12902200	-0.93886400
C	0.07927200	-1.81262600	1.75416800
C	-1.10725700	-1.75829600	2.49900000
C	1.27422600	-2.09471000	2.42441000
C	-1.09913000	-1.98208100	3.86952900
H	-2.04292700	-1.51208700	1.99443100
C	1.28232200	-2.32299500	3.79678300

H	2.20296100	-2.15439100	1.85794300
C	0.09699900	-2.27121000	4.52394200
H	-2.03066300	-1.93118300	4.43077600
H	2.22145200	-2.55012500	4.29905100
H	0.10328200	-2.45536400	5.59684800
C	-0.33281700	-0.28045600	-3.74371000
C	0.64158000	-0.49250200	-4.72732200
C	-1.41069300	0.56571200	-4.04116400
C	0.53989300	0.11323200	-5.97408000
H	1.48047300	-1.15956500	-4.53154800
C	-1.50313800	1.17734800	-5.28274300
H	-2.15059000	0.78615800	-3.27271300
C	-0.53188100	0.95235700	-6.25566700
H	1.30438700	-0.07124100	-6.72715300
H	-2.33702100	1.84625400	-5.48773900
H	-0.60904700	1.43503800	-7.22855400
H	4.55456600	1.00647700	1.90735900
C	-0.45219700	1.82340500	2.24134200
H	-1.13340300	2.60531200	1.84568900
H	-1.11757700	0.99938300	2.54293300
C	0.38605000	2.32976400	3.38538600
C	0.73183700	3.67902700	3.47450100
C	0.89662100	1.43373900	4.32604400
C	1.56694000	4.12719400	4.49331400
H	0.34962600	4.37921200	2.73141000
C	1.73110700	1.88072700	5.34452600
H	0.63588300	0.37633100	4.25423700
C	2.06816900	3.22892400	5.43065100
H	1.82505900	5.18319400	4.55442400
H	2.12064100	1.17198100	6.07423600
H	2.72004300	3.57965500	6.22936900

A7*(Conformer of A7): 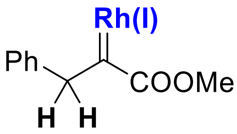

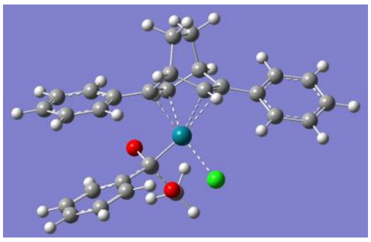

C	1.17191500	-1.21255500	0.37853800
C	1.05414800	-1.67262600	1.78219700

O	0.54022100	-2.90018200	1.85448900
O	1.37986600	-1.00151800	2.74227900
Rh	-0.36423500	-0.34813100	-0.28567000
C	-2.05713800	3.12084700	1.06133400
C	-1.94584400	1.57992700	1.17159400
C	-0.64426600	2.37524500	-0.88862000
C	-1.27595600	3.59899800	-0.17713300
H	-3.11764400	3.40310500	0.99558800
H	-1.65972000	3.57340800	1.97991700
H	-1.93173600	4.12527400	-0.88294900
H	-0.47232300	4.29523500	0.10209700
C	-2.47343300	0.97512800	-0.11929200
C	-1.73497600	1.37456600	-1.20293200
H	-2.02097500	1.15417600	-2.22975300
H	-0.09926100	2.67844900	-1.78869200
H	-2.47038800	1.19583200	2.05186600
C	0.26455800	1.68973800	0.12325000
C	-0.47945700	1.20554000	1.21729600
H	-0.01346900	0.92658400	2.16284200
Cl	-1.00507900	-2.07197400	-1.85722000
C	1.71522800	1.95832300	0.13445900
C	2.44140800	1.99716600	-1.06259800
C	2.40078800	2.16821700	1.33613500
C	3.80948200	2.23322300	-1.05832200
H	1.93019000	1.80486200	-2.00671000
C	3.77032700	2.40590700	1.34067000
H	1.85203700	2.14951000	2.27647800
C	4.48022900	2.43893500	0.14461800
H	4.35718900	2.24513500	-1.99965400
H	4.28362800	2.57124600	2.28670900
H	5.55256900	2.62752000	0.14835800
C	-3.74777700	0.24379200	-0.17848600
C	-4.70183100	0.40595500	0.83444600
C	-4.07213500	-0.56771100	-1.27560700
C	-5.94298200	-0.21472100	0.75281400
H	-4.48925100	1.04498100	1.69051800
C	-5.30706100	-1.19480300	-1.34855400
H	-3.32764900	-0.74912800	-2.04867100
C	-6.24967000	-1.01944300	-0.33814500
H	-6.67268200	-0.06709800	1.54742000
H	-5.53125000	-1.83470100	-2.20016800
H	-7.21828500	-1.51278700	-0.40055200
C	2.44783900	-1.34543600	-0.27944300
C	3.64125100	-1.45351900	0.46451300

C	2.51491500	-1.35554900	-1.68778100
C	4.86311400	-1.53169400	-0.18310100
H	3.59581100	-1.42738700	1.55235400
C	3.73872000	-1.47919500	-2.32580800
H	1.58223600	-1.31210000	-2.24990100
C	4.91156800	-1.55352800	-1.57663100
H	5.78270000	-1.58753300	0.39641900
H	3.78216500	-1.51397300	-3.41265300
H	5.87322700	-1.63555800	-2.08149400
C	0.34993700	-3.40765600	3.17311000
H	-0.08228200	-4.40141700	3.04691200
H	1.30542400	-3.46735700	3.70559200
H	-0.33143400	-2.76164800	3.73675300

Optimized structures and coordinates in Scheme 4

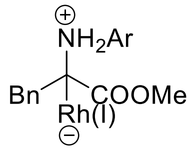

A9:

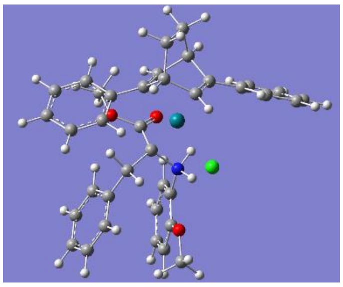

C	0.10427400	1.32557000	-0.25622900
C	0.70318100	1.26819600	-1.59171700
O	0.09172300	1.02488700	-2.62673300
O	2.03837300	1.46453000	-1.58504300
C	2.66647100	1.42866900	-2.85614400
H	2.53717300	0.45233100	-3.34023500
H	2.24990300	2.19811200	-3.51745200
Rh	0.12378100	-0.80000100	0.40081000
C	1.95384600	-4.13099100	-0.73346400
C	0.97089200	-2.98666800	-1.05704900
C	1.91292400	-2.67626800	1.32113900
C	2.56209300	-3.91212900	0.66485000
H	1.41476100	-5.08746600	-0.78636800
H	2.73582700	-4.16302100	-1.50457900
H	2.40558200	-4.78920500	1.30714600
H	3.64796000	-3.74775000	0.60400100
C	-0.12060100	-2.94292300	0.00678400

C	0.40331000	-2.77347100	1.29162100
H	-0.19042800	-2.88688500	2.19724200
H	2.29583300	-2.51746900	2.33568200
H	0.55029200	-3.09749500	-2.06262200
C	2.17479100	-1.46222200	0.42918500
C	1.63555700	-1.63817000	-0.86426100
H	1.95251500	-1.04434300	-1.72089100
Cl	-1.92793000	-0.36313700	1.74560600
C	-2.83428700	5.02823400	-0.10311800
C	-2.39336400	3.74055400	0.19253600
C	-1.84686300	2.95793600	-0.83327800
C	-1.77383800	3.43025200	-2.13369000
C	-2.21945300	4.71690300	-2.42303200
C	-2.73721900	5.50888000	-1.40627600
H	-3.24434800	5.66002400	0.68061500
H	-1.35105000	2.78873300	-2.90418400
H	-2.16037300	5.09415400	-3.44135700
H	-3.08167700	6.51847400	-1.62334900
N	-1.38515100	1.61073800	-0.48928400
H	-1.88213500	1.25062500	0.35667600
H	-1.62531800	0.97666700	-1.26300000
O	-2.44720900	3.15497400	1.40420200
C	-3.03614700	3.86224900	2.46987700
H	-2.99438100	3.19327300	3.33250800
H	-2.47704600	4.78461100	2.68770800
H	-4.08445000	4.11137700	2.24953000
H	3.72600300	1.62151100	-2.66823100
C	3.22593100	-0.49887100	0.79995300
C	4.20189100	-0.09688800	-0.11664200
C	3.26794400	0.03527700	2.09505200
C	5.17156600	0.83430700	0.23677400
H	4.20137500	-0.53196600	-1.11579200
C	4.23622800	0.96489400	2.45011800
H	2.49719600	-0.25169600	2.81218700
C	5.18892200	1.37333700	1.51935500
H	5.92259300	1.13644900	-0.49244500
H	4.24249100	1.38105100	3.45627300
H	5.94590700	2.10512800	1.79609400
C	-1.51917600	-3.27946500	-0.30782400
C	-2.08833000	-2.86907900	-1.52119300
C	-2.31702000	-3.98261800	0.59949200
C	-3.41651200	-3.14912400	-1.81299700
H	-1.48883500	-2.29152900	-2.22720000
C	-3.64439900	-4.26613500	0.30582100

H	-1.88709600	-4.31508400	1.54331900
C	-4.19995700	-3.85037400	-0.90023400
H	-3.84388800	-2.81364100	-2.75690100
H	-4.24976400	-4.81585600	1.02477400
H	-5.24118500	-4.07217700	-1.12891000
C	0.74256100	2.16200600	0.83742400
H	0.29849500	1.85923800	1.79780500
H	1.78886000	1.82322800	0.88486800
C	0.73905100	3.67596400	0.76479900
C	0.43013200	4.40425200	1.91633700
C	1.08120700	4.38725900	-0.38830700
C	0.44216400	5.79548300	1.91845800
H	0.17531900	3.86237400	2.82924800
C	1.08657200	5.77775700	-0.39504000
H	1.34557900	3.84723300	-1.29762500
C	0.76421700	6.48975200	0.75692700
H	0.19870500	6.33834600	2.83187400
H	1.34674800	6.31022200	-1.30925100
H	0.76985200	7.57863800	0.74941400

C1: 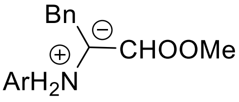

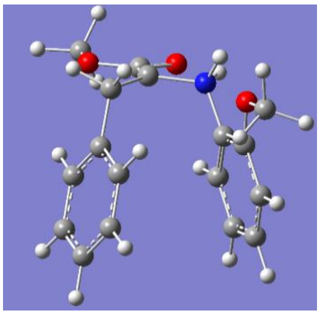

C	-6.76574500	0.45010800	1.13877500
C	-5.54356800	0.69025500	0.52038100
O	-5.39002700	0.73321600	-0.72306900
O	-4.48824300	0.86375400	1.36952800
C	-3.23663800	1.01776300	0.73054300
H	-2.97860000	0.13367700	0.13448800
H	-3.23107700	1.88973700	0.06547200
C	-11.00594100	2.28577200	0.24954600
C	-10.09961300	1.22959700	0.30174600
C	-8.76091500	1.47065800	-0.00709800
C	-8.31032100	2.73359700	-0.34128800
C	-9.21532300	3.78752400	-0.38815700
C	-10.55486100	3.55651800	-0.09621600

H	-12.05507000	2.12719000	0.48572600
H	-7.24869200	2.86964600	-0.54640100
H	-8.87382700	4.78576100	-0.65038400
H	-11.26743100	4.37833400	-0.12932300
N	-7.80739800	0.36456400	0.08989400
H	-8.34033600	-0.51166400	0.13776900
H	-7.18122600	0.35282000	-0.75670200
O	-10.40291800	-0.05060300	0.64884200
C	-11.73034200	-0.33053400	1.03204800
H	-11.75873700	-1.39134000	1.29117600
H	-12.02388200	0.26546300	1.90854300
H	-12.43435400	-0.13852500	0.20976200
H	-2.50293700	1.15286000	1.53046600
C	-7.18574800	0.55481400	2.56148300
H	-6.31468800	0.27466500	3.17169000
H	-7.96729000	-0.19189500	2.79406700
C	-7.67269900	1.92354000	2.99512400
C	-9.00919900	2.16371800	3.30473900
C	-6.77319200	2.99197400	3.05021400
C	-9.44565300	3.43881700	3.65662500
H	-9.72095800	1.33527500	3.25922200
C	-7.19992100	4.26214800	3.41291100
H	-5.72818200	2.80875700	2.79646200
C	-8.54110900	4.49196000	3.71554000
H	-10.49813300	3.60836300	3.88302100
H	-6.48328100	5.08137500	3.46033500
H	-8.87712700	5.48921700	3.99573000

A10: 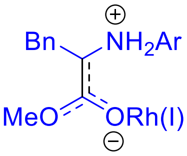

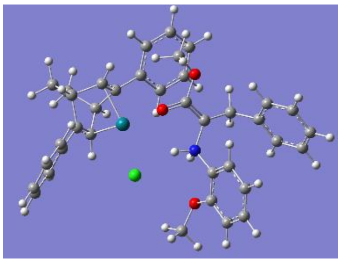

C	-2.25101100	1.05106100	0.63416500
C	-1.12557300	1.73484200	1.02964500
O	-0.03479500	1.81003700	0.37857200
O	-1.24732100	2.40212000	2.21307500
C	-0.19655300	3.28279000	2.54327500

H	0.78367200	2.80690500	2.43018700
H	-0.21662100	4.17971600	1.90870700
Rh	1.39825200	0.22134800	-0.01963100
C	4.62824700	-1.06121800	1.82587500
C	3.93103900	0.03658800	0.99868100
C	2.27327000	-1.88840900	1.47401500
C	3.62401400	-2.18289000	2.15497200
H	5.47770900	-1.44822000	1.24506300
H	5.04223500	-0.61979700	2.74265100
H	3.99710600	-3.15920900	1.81692900
H	3.45933800	-2.26177000	3.23920600
C	3.37065900	-0.57954000	-0.28215400
C	2.45208200	-1.61244900	-0.00479100
H	2.11107400	-2.31061300	-0.76956800
H	1.54700700	-2.68807900	1.66041800
H	4.61400800	0.86490200	0.77918200
C	1.74904000	-0.53521500	1.95373700
C	2.65749700	0.50749700	1.67205000
H	2.58860600	1.49248200	2.13563900
Cl	0.49856300	-0.06216700	-2.31216200
C	-4.05970300	0.17948300	-3.82674600
C	-3.28543600	-0.17600100	-2.72573800
C	-2.98961600	0.79258300	-1.76535400
C	-3.42755200	2.09523900	-1.88973700
C	-4.21344200	2.44450600	-2.98319900
C	-4.52316400	1.48764500	-3.94289600
H	-4.30006300	-0.55871900	-4.58873200
H	-3.16500000	2.81503700	-1.11718000
H	-4.58161800	3.46328300	-3.07948200
H	-5.13369900	1.75808900	-4.80267200
N	-2.10815600	0.38313400	-0.66070100
H	-2.17958400	-0.64275600	-0.58983500
H	-1.12784400	0.52953300	-1.00937400
O	-2.79196400	-1.41644800	-2.48536600
C	-2.51801000	-2.23592400	-3.60304800
H	-1.91454800	-3.06888900	-3.23463100
H	-3.44246100	-2.62890500	-4.05032800
H	-1.94327700	-1.67889900	-4.35481400
C	0.63725200	-0.45147900	2.91806900
C	0.72220100	0.36580000	4.05012000
C	-0.52774100	-1.20702900	2.72778500
C	-0.32685500	0.43535400	4.95952800
H	1.63272200	0.93951400	4.22420500
C	-1.57096400	-1.14695800	3.64148300

H	-0.61309400	-1.83541500	1.83887500
C	-1.47625000	-0.32228000	4.76028300
H	-0.24095700	1.07738100	5.83508600
H	-2.46968200	-1.73995200	3.47602900
H	-2.29600200	-0.27314000	5.47497600
C	4.01799400	-0.35231400	-1.58565700
C	4.26771700	-1.41431200	-2.45924400
C	4.40388500	0.93718500	-1.97017700
C	4.88656500	-1.19519300	-3.68417200
H	3.98403400	-2.42478400	-2.16658500
C	5.01617100	1.15751600	-3.19573300
H	4.18170900	1.77891000	-1.31324800
C	5.26254300	0.09097600	-4.05666500
H	5.07738800	-2.03497300	-4.35061900
H	5.29557900	2.16925600	-3.48533800
H	5.74430600	0.26339300	-5.01780400
H	-0.35545900	3.56500800	3.58840500
C	-3.50772700	0.80508700	1.40136100
H	-3.28291600	0.99599800	2.46061300
H	-3.78173000	-0.26649000	1.34078400
C	-4.70004700	1.63858900	0.98619700
C	-5.79525000	1.07702900	0.33503000
C	-4.68659600	3.01783600	1.21185200
C	-6.84969200	1.87408200	-0.10414800
H	-5.80906100	0.00160600	0.14558900
C	-5.73679600	3.81713300	0.77834300
H	-3.82286100	3.45555100	1.71627300
C	-6.82191300	3.24679900	0.11388300
H	-7.69297600	1.41950400	-0.62260100
H	-5.71221800	4.89115100	0.95948800
H	-7.64487700	3.87274500	-0.22813000

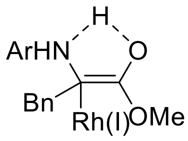

TS-5:

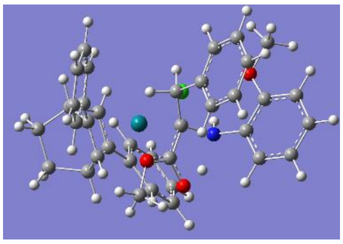

C	0.88560200	-0.11084700	0.40250200
C	0.56397900	0.24938300	1.73318700

O	0.27852400	-0.70920100	2.55612500
O	0.59476200	1.49697800	2.16502700
C	0.35087400	1.70924000	3.55247200
H	-0.62326800	1.30688400	3.85322900
H	1.13249800	1.23261000	4.15453700
Rh	-1.31612700	0.02418800	-0.31906200
C	-4.79908600	1.56705500	0.78686200
C	-3.62942400	0.60580300	1.07834600
C	-3.20331000	1.84318000	-1.14276700
C	-4.51894900	2.34371900	-0.51299300
H	-5.72761100	0.98392400	0.70970200
H	-4.92242600	2.25138100	1.63746600
H	-5.33555900	2.21489700	-1.23610600
H	-4.42928600	3.42256000	-0.32018700
C	-3.44967600	-0.34911100	-0.09640200
C	-3.21167800	0.33773600	-1.29700100
H	-3.24028700	-0.14760600	-2.27133500
H	-3.00012700	2.35001800	-2.09257900
H	-3.78626600	0.06398800	2.01774600
C	-2.06613900	2.04201200	-0.14172700
C	-2.30588600	1.34130000	1.06085100
H	-1.78651000	1.57989500	1.98733200
Cl	-0.79372300	-1.78690600	-1.91610700
C	4.62213100	-2.71570600	-0.31094000
C	3.30261600	-2.29742700	-0.47060900
C	2.52146500	-1.99991500	0.65933500
C	3.07210700	-2.14617900	1.92594600
C	4.39210200	-2.55985500	2.08810800
C	5.16059100	-2.83992600	0.96740400
H	5.23732800	-2.93176800	-1.18123600
H	2.45822900	-1.92302600	2.79744600
H	4.81044300	-2.66805600	3.08674100
H	6.19418700	-3.16381300	1.07824000
N	1.15440200	-1.57088600	0.49503200
H	0.69578700	-2.05064700	-0.29127800
H	0.51247500	-1.51453500	1.79128900
O	2.68398700	-2.13128700	-1.65754300
C	3.37582600	-2.47473300	-2.83086900
H	2.67458400	-2.30404500	-3.65172000
H	4.26867200	-1.84504600	-2.96922300
H	3.67762700	-3.53301500	-2.82400200
H	0.37591600	2.79291400	3.68904700
C	-1.10383900	3.14341400	-0.31280000
C	-0.71568200	3.94542700	0.76563100

C	-0.54471900	3.40423900	-1.57157400
C	0.21760200	4.96142100	0.59869000
H	-1.16087100	3.77031300	1.74480200
C	0.38759500	4.41921900	-1.74000400
H	-0.81311900	2.76758400	-2.41581400
C	0.77526500	5.19994600	-0.65359700
H	0.50705700	5.57496100	1.45087800
H	0.82323600	4.59466200	-2.72225800
H	1.50921300	5.99323900	-0.78414500
C	-3.76765200	-1.78150300	0.02130200
C	-3.37687100	-2.49507400	1.16179600
C	-4.43943800	-2.46040700	-0.99858900
C	-3.64691400	-3.85151000	1.27471100
H	-2.81516600	-1.98423600	1.94581000
C	-4.71507300	-3.81702400	-0.88253000
H	-4.74987800	-1.91497300	-1.88889100
C	-4.31990400	-4.51680500	0.25297600
H	-3.32447700	-4.39560400	2.16113100
H	-5.23926300	-4.33147300	-1.68635400
H	-4.53258400	-5.58097700	0.34115000
C	1.64103100	0.79672600	-0.54276900
H	1.37572200	0.51422100	-1.57196100
H	1.23244800	1.80961500	-0.39737500
C	3.14972900	0.87408800	-0.44775800
C	3.90487700	0.85643100	-1.62147400
C	3.82229000	1.01495000	0.76713700
C	5.29212800	0.95725800	-1.58820400
H	3.38870500	0.75357700	-2.57783900
C	5.20884700	1.10993300	0.80831800
H	3.25373200	1.03869800	1.69790700
C	5.95080000	1.07704100	-0.36899500
H	5.85992500	0.93820000	-2.51826100
H	5.71405900	1.20746600	1.76864600
H	7.03686900	1.14831600	-0.33453400

B1:

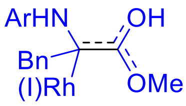

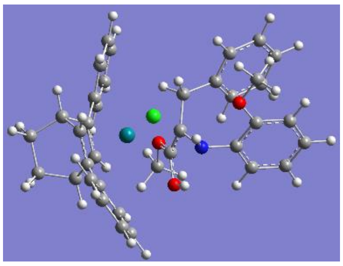

C	0.91492200	-0.10919400	0.35563500
C	0.54286700	0.34497100	1.61684500
O	0.14720600	-0.50479200	2.55836000
O	0.63924200	1.61531100	1.98309400
C	0.56632900	1.91953600	3.37016200
H	-0.39419300	1.61574100	3.80271900
H	1.37826700	1.42195300	3.91495100
Rh	-1.34314200	0.11540200	-0.32966100
C	-4.85808600	1.50030000	0.87324200
C	-3.65621600	0.56708600	1.11937200
C	-3.28925500	1.90622100	-1.05390300
C	-4.61415200	2.33879000	-0.39544700
H	-5.76796700	0.89096800	0.77901200
H	-4.99653100	2.14473900	1.75212000
H	-5.43192100	2.21377200	-1.11781900
H	-4.55760000	3.41090100	-0.15848200
C	-3.45011000	-0.33527400	-0.09164200
C	-3.24709000	0.40889000	-1.26864500
H	-3.27063600	-0.03657100	-2.26194700
H	-3.10690600	2.45658600	-1.98349900
H	-3.78840300	-0.01355000	2.03888700
C	-2.14796400	2.09521200	-0.05595200
C	-2.35474400	1.34141100	1.12099300
H	-1.83243000	1.55098500	2.05290800
Cl	-0.78128200	-1.50795200	-2.10384200
C	4.58285900	-2.82874700	-0.17465500
C	3.28429300	-2.38934900	-0.41737300
C	2.45503800	-2.00098000	0.65192600
C	2.95223400	-2.08002100	1.94754600
C	4.25425900	-2.50963800	2.19492900
C	5.06406800	-2.88300600	1.13212500
H	5.23036800	-3.11189000	-1.00124700
H	2.31005300	-1.78826400	2.77845800
H	4.62550400	-2.56039700	3.21679900
H	6.08269800	-3.22393300	1.31024700
N	1.12142500	-1.55201800	0.38897700
H	0.76170500	-1.96387000	-0.47752400
O	2.72321500	-2.27455600	-1.64301800
C	3.47492700	-2.65922100	-2.76436300
H	2.82749600	-2.49775500	-3.63036700
H	4.38513900	-2.04716700	-2.86712700
H	3.75846600	-3.72173400	-2.71568500
H	0.68290400	3.00399600	3.43383600
C	-1.19732900	3.21005600	-0.20296400

C	-0.82881900	3.99982600	0.89033000
C	-0.62742800	3.49041500	-1.45237600
C	0.09855700	5.02476600	0.74743800
H	-1.28571400	3.80816800	1.86114600
C	0.30009700	4.51362200	-1.59627700
H	-0.88260800	2.86248800	-2.30740100
C	0.66970600	5.28229700	-0.49509500
H	0.37313100	5.63070500	1.61029700
H	0.74607200	4.70452200	-2.57087700
H	1.39992500	6.08182200	-0.60710900
C	-3.70629200	-1.78312500	-0.02796900
C	-3.31694100	-2.51482500	1.10194600
C	-4.31361400	-2.46030600	-1.08911700
C	-3.52722100	-3.88492800	1.16617900
H	-2.80713200	-2.00407500	1.92079900
C	-4.53069600	-3.83039600	-1.02147800
H	-4.62037700	-1.90388900	-1.97364200
C	-4.13867900	-4.54715000	0.10477400
H	-3.20813000	-4.44123000	2.04616700
H	-5.00751900	-4.34225500	-1.85571800
H	-4.30576300	-5.62176800	0.15487300
C	1.64665700	0.77093600	-0.63713900
H	1.36613400	0.43060000	-1.64477100
H	1.25534900	1.79615600	-0.53703900
C	3.15726600	0.81421100	-0.55231800
C	3.90826200	0.72318000	-1.72519300
C	3.83707700	0.98231800	0.65516900
C	5.29812900	0.78364700	-1.69715900
H	3.38689700	0.59138600	-2.67501500
C	5.22556800	1.03767600	0.69119000
H	3.27210700	1.05444100	1.58581200
C	5.96321000	0.93601300	-0.48510000
H	5.86252700	0.70774600	-2.62649200
H	5.73572000	1.15622900	1.64651100
H	7.05102100	0.97584600	-0.45521800
H	0.20337600	-1.37594000	2.07518600

B3: 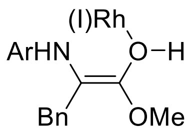

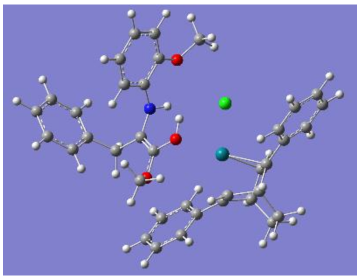

C	-2.68389900	0.44027000	0.55448700
C	-1.53580600	1.08616300	0.81195300
O	-0.53665600	1.26229900	-0.11050400
O	-1.23545800	1.61771100	2.00470600
C	-0.81727700	2.97423100	1.96910400
H	0.17990600	3.07561300	1.52114400
H	-1.53247400	3.58595500	1.39807600
Rh	1.30244900	-0.09800800	0.01174900
C	4.76976700	-0.50079900	1.84465000
C	3.86886700	0.31597500	0.89661400
C	2.57354000	-1.73650200	1.78997400
C	3.98102600	-1.69417300	2.41692400
H	5.65009500	-0.84079500	1.28085500
H	5.13735100	0.15244500	2.64739300
H	4.49751400	-2.64297600	2.21890800
H	3.87238700	-1.61077700	3.50774600
C	3.36996300	-0.58856100	-0.22568000
C	2.64254000	-1.69227000	0.27880600
H	2.39274300	-2.55695200	-0.33656300
H	1.99611500	-2.59461600	2.15165500
H	4.39609200	1.19197000	0.50389200
C	1.85751400	-0.42006500	2.08091800
C	2.56024600	0.68545900	1.56588000
H	2.34467000	1.70911000	1.87562800
Cl	0.35919900	-0.78931800	-2.12336100
C	-3.33856800	0.84206400	-4.27729500
C	-3.12564500	0.09820500	-3.12621600
C	-3.17027900	0.71518000	-1.85686600
C	-3.46036400	2.07698400	-1.78598200
C	-3.68898100	2.81519100	-2.94648800
C	-3.62290000	2.20648500	-4.19028900
H	-3.28652700	0.35902600	-5.25089700
H	-3.52089200	2.55518000	-0.80776300
H	-3.91814200	3.87637000	-2.86419300
H	-3.79193400	2.78026400	-5.09951400
N	-2.92174900	-0.08450300	-0.73901000
H	-2.54835800	-1.00032800	-0.96864600

O	-2.86990700	-1.23947200	-3.10390900
C	-2.35119600	-1.81915500	-4.27542000
H	-2.00716400	-2.82001200	-4.00056500
H	-3.11935800	-1.90503900	-5.05973500
H	-1.49776500	-1.23944700	-4.65569700
C	0.79953400	-0.34236200	3.10175200
C	0.83531400	0.64166200	4.09459500
C	-0.25810400	-1.25930000	3.09990600
C	-0.16982400	0.72059600	5.05084700
H	1.67141900	1.34107800	4.12028900
C	-1.26897300	-1.17243600	4.04710800
H	-0.30488100	-2.01888200	2.31788800
C	-1.23028100	-0.17951800	5.02343300
H	-0.12311000	1.48704700	5.82334000
H	-2.09724400	-1.87931100	4.02012200
H	-2.02153800	-0.11500200	5.76856100
C	3.91696900	-0.49449400	-1.58997100
C	4.24648000	-1.64630400	-2.31062900
C	4.12283500	0.75401500	-2.18927900
C	4.76826800	-1.55358300	-3.59465400
H	4.10466500	-2.62493900	-1.85369200
C	4.63791400	0.84682400	-3.47414100
H	3.83796900	1.65951300	-1.65255300
C	4.96458700	-0.30766900	-4.18097900
H	5.02357800	-2.46051700	-4.14027200
H	4.77796800	1.82526700	-3.93066200
H	5.37083000	-0.23468100	-5.18838900
H	-0.79091900	3.31365300	3.00845500
C	-3.77640100	0.31569100	1.58389300
H	-3.35889300	0.46961300	2.58778000
H	-4.17170100	-0.71098200	1.54236000
C	-4.88058800	1.30534200	1.31817400
C	-5.94968400	0.98753800	0.48149700
C	-4.79208000	2.59989500	1.83592200
C	-6.90484200	1.94618500	0.15798100
H	-6.01598900	-0.01707800	0.06205900
C	-5.74633800	3.55946200	1.51780700
H	-3.95566000	2.84937800	2.49253900
C	-6.80527100	3.23450300	0.67343200
H	-7.72954700	1.68623500	-0.50392400
H	-5.66579600	4.56420200	1.93125100
H	-7.55278100	3.98481400	0.42051500
H	-0.83100900	0.91142100	-0.98622300

B2:

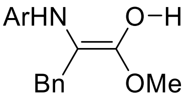

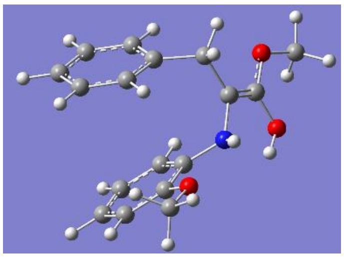

C	-2.24222900	0.86888500	0.59695700
C	-1.26199300	1.62404600	1.13137300
O	-0.15009100	1.95296200	0.45119900
O	-1.33185700	2.12130500	2.37386400
C	-0.15870500	2.70513800	2.91386800
H	0.67450000	1.99184400	2.92808300
H	0.14386700	3.59238300	2.34573400
C	-3.95766000	0.99399100	-3.89836400
C	-3.26691100	0.37825700	-2.86353000
C	-2.75140600	1.13631900	-1.79624300
C	-2.93999400	2.51268500	-1.80212200
C	-3.64260200	3.13043300	-2.83279900
C	-4.14708900	2.37610200	-3.88142300
H	-4.36202100	0.40507100	-4.71850500
H	-2.55730900	3.09512300	-0.96472400
H	-3.79398700	4.20785100	-2.80722600
H	-4.69306600	2.85224200	-4.69349800
N	-2.03939000	0.47756600	-0.76867400
H	-2.07723700	-0.52998200	-0.90478800
O	-3.05956000	-0.97108300	-2.75893500
C	-3.65590400	-1.79639000	-3.72475400
H	-3.41577100	-2.82652900	-3.44791500
H	-4.74987500	-1.67034600	-3.74049900
H	-3.26008500	-1.59448500	-4.73159700
H	-0.41601700	2.99136800	3.93646300
C	-3.49600000	0.45995100	1.31248600
H	-3.39147200	0.69551500	2.38032300
H	-3.61861800	-0.63499200	1.23925600
C	-4.71857200	1.13645700	0.74235700
C	-5.47534800	0.52704300	-0.25654500
C	-5.04615400	2.43417700	1.13626600
C	-6.52586600	1.20502500	-0.86611500
H	-5.20642500	-0.47931000	-0.58670000
C	-6.10093800	3.11223800	0.53805300
H	-4.44600000	2.91891600	1.90885600

C	-6.84092200	2.50036900	-0.47039200
H	-7.09165600	0.72240700	-1.66220700
H	-6.34497900	4.12548600	0.85479000
H	-7.66206300	3.03311700	-0.94747100
H	-0.25148500	1.47795100	-0.40089900

TS-6: 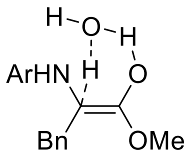

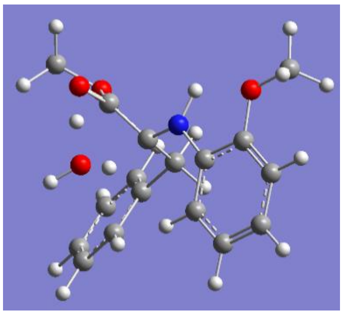

C	-7.36140500	1.27478800	1.59833700
C	-6.14884100	0.83239000	0.99324000
O	-6.08595900	0.31452800	-0.16999900
O	-5.01734500	1.08263200	1.64356300
C	-3.80074900	0.93842500	0.91764600
H	-3.62937900	-0.10978100	0.65253500
H	-3.81921000	1.53835500	0.00161100
C	-12.01708900	-0.24053600	1.90122000
C	-10.65191200	-0.46829100	1.86607000
C	-9.74590200	0.57911200	1.57383300
C	-10.27339500	1.84771300	1.33820000
C	-11.64890200	2.07425900	1.37100900
C	-12.52315200	1.03850300	1.65091800
H	-12.70133500	-1.05605000	2.12413200
H	-9.59538400	2.66790700	1.10963800
H	-12.02796500	3.07642100	1.17839800
H	-13.59738700	1.20803400	1.68101700
N	-8.39570000	0.27634900	1.53487300
H	-8.18636800	-0.63364700	1.93045900
O	-10.05810800	-1.67750400	2.10573200
C	-10.89123700	-2.75867300	2.42815100
H	-10.23633200	-3.61631700	2.60418800
H	-11.47845800	-2.55930200	3.33787400
H	-11.58192600	-2.99634800	1.60478200
H	-3.01608500	1.29660000	1.58732500
C	-7.26116300	2.07376100	2.88924900

H	-6.80711700	1.47511700	3.69752600
H	-8.29272500	2.28275500	3.21265300
C	-6.51455100	3.37538600	2.75553200
C	-6.95341800	4.36189400	1.86852700
C	-5.37304900	3.63516100	3.51380700
C	-6.27714800	5.57054200	1.74687600
H	-7.85054800	4.18194700	1.27280200
C	-4.69174200	4.84214300	3.39841500
H	-5.01309800	2.86956600	4.20235100
C	-5.14122600	5.81562300	2.51247300
H	-6.64339000	6.32849500	1.05536800
H	-3.80439900	5.02319900	4.00358200
H	-4.61168500	6.76255100	2.42049500
H	-7.03619400	0.84811600	-0.75581700
O	-7.87422200	1.69018300	-0.91532500
H	-7.75571400	1.88074100	0.20967200
H	-7.45432000	2.43242900	-1.37629200

TS-7:

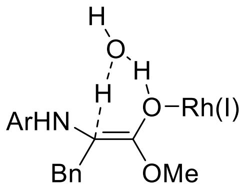

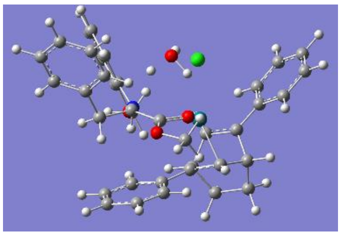

C	-2.25657200	1.85410100	2.05975900
C	-0.99756300	2.46613400	2.07546800
O	-0.04732700	2.27754500	1.24103600
O	-0.84707300	3.46725900	2.97817300
C	0.35256000	4.21316100	2.90865100
H	1.23079700	3.57219000	3.05763600
H	0.45995500	4.72234900	1.94269800
Rh	0.38504600	0.24508800	0.33973600
C	3.56109900	-1.94111200	1.15135900
C	2.97298000	-0.63932200	0.57767700
C	1.07138200	-2.13725000	1.46928200
C	2.42828200	-2.82234900	1.71130500
H	4.10924100	-2.46002700	0.35224900
H	4.29288600	-1.69385300	1.93232100
H	2.42388300	-3.81104100	1.23244100

H	2.55326100	-2.99105600	2.79040700
C	1.94819500	-0.96246600	-0.50374000
C	0.89334800	-1.75867700	0.00925600
H	0.19469500	-2.29283600	-0.63606500
H	0.24150200	-2.76120700	1.81648000
H	3.76262600	0.02145300	0.20482100
C	1.05974100	-0.75375300	2.12010100
C	2.08434200	0.06188300	1.58224700
H	2.39000500	1.00756800	2.02923200
Cl	-0.88200100	0.57483800	-1.80626500
C	-4.39101100	-1.68309300	-0.57664900
C	-3.38911600	-1.21849000	0.25979500
C	-3.35696600	0.13472500	0.66392400
C	-4.37331700	0.97894900	0.21925400
C	-5.37703200	0.50953500	-0.62869800
C	-5.38936900	-0.81527900	-1.02790600
H	-4.40306000	-2.72209100	-0.89653900
H	-4.39524400	2.01586500	0.55394800
H	-6.15421300	1.19465600	-0.96248000
H	-6.16853700	-1.19116300	-1.68798300
N	-2.28593900	0.55528000	1.44891700
H	-1.91677400	-0.20018100	2.02447600
O	-2.36740900	-1.99035900	0.74790600
C	-2.27046900	-3.29652300	0.24674300
H	-1.37665200	-3.74343200	0.69595700
H	-3.14556300	-3.90673200	0.51948400
H	-2.16310100	-3.29371900	-0.84942000
C	0.29915500	-0.45024500	3.34695700
C	0.59361400	0.68368200	4.12029300
C	-0.77789800	-1.25220200	3.75600900
C	-0.16573000	1.01605900	5.23323600
H	1.43909300	1.31469500	3.85058900
C	-1.53936200	-0.91913700	4.87143800
H	-1.05454100	-2.13209100	3.17622200
C	-1.24180700	0.21865000	5.61358400
H	0.08183000	1.91187600	5.80141200
H	-2.37661900	-1.55407600	5.15681700
H	-1.84222800	0.48250700	6.48234700
C	2.25481000	-0.78088000	-1.93299900
C	1.80932100	-1.69801900	-2.89002600
C	2.99005000	0.32941800	-2.36560900
C	2.08440000	-1.50881000	-4.23807500
H	1.24226300	-2.57238900	-2.57365800
C	3.26278900	0.52133900	-3.71238200

H	3.31638300	1.07365400	-1.63885500
C	2.81016400	-0.39801300	-4.65486900
H	1.72570700	-2.23303700	-4.96772900
H	3.82507300	1.39807700	-4.02992900
H	3.02136200	-0.24705100	-5.71223700
H	0.28886000	4.95277800	3.71084300
C	-3.27218100	2.16054100	3.14964500
H	-2.74342700	2.45999600	4.06943400
H	-3.79356700	1.22267800	3.39656500
C	-4.30249100	3.21479400	2.81649700
C	-5.66066800	2.98006600	3.03808300
C	-3.92640500	4.44668200	2.27238200
C	-6.61699300	3.94374600	2.73165000
H	-5.97212700	2.01573900	3.44295200
C	-4.87812500	5.41241400	1.96275500
H	-2.86939700	4.65072400	2.09924200
C	-6.22876600	5.16539600	2.19183400
H	-7.67138300	3.73424000	2.90802800
H	-4.56183600	6.36533200	1.53983900
H	-6.97458800	5.92030100	1.94767400
H	-0.94212400	2.98323600	-0.15690800
O	-1.88927900	2.98512200	-0.48779300
H	-1.86312000	2.16080300	-1.09509600
H	-2.33440800	2.67963800	0.42265900

B4: 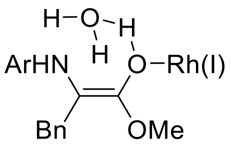

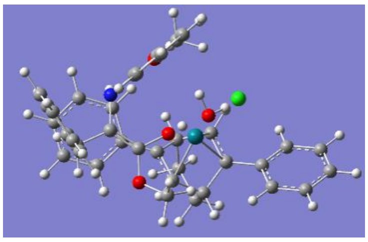

C	-1.53772900	1.25158300	1.67716000
C	-0.52549400	1.46340400	0.82241500
O	-0.36787300	0.70707100	-0.29628400
O	0.44588900	2.38423600	1.04548900
C	0.83339500	3.12188100	-0.10508400
H	1.34344200	2.48140600	-0.83900700
H	-0.03967300	3.58568600	-0.58790800
Rh	1.11565200	-0.82646700	-0.70278800
C	4.67502600	-1.68038200	0.74130900

C	3.77303200	-0.71655500	-0.05451000
C	2.38059700	-2.71941200	0.79888200
C	3.83581400	-2.85103600	1.28900300
H	5.47249100	-2.04194000	0.07672200
H	5.16410200	-1.12936800	1.55592300
H	4.24347200	-3.81723900	0.96230200
H	3.83555000	-2.85835600	2.38831300
C	3.09392000	-1.47565800	-1.19011200
C	2.31894400	-2.55531600	-0.70701500
H	1.94642500	-3.34601700	-1.35956900
H	1.76396500	-3.55699500	1.14523000
H	4.34129600	0.14286900	-0.42740700
C	1.81531400	-1.37613800	1.26233700
C	2.56863800	-0.29424700	0.76172900
H	2.45383300	0.72357200	1.14082700
Cl	0.05070900	-1.18773900	-2.90109500
C	-3.56955300	-1.15902600	-1.83467400
C	-2.95323000	-1.05799800	-0.59221900
C	-3.09975400	0.11158200	0.18489800
C	-3.86467100	1.16049500	-0.31846100
C	-4.47723800	1.06232800	-1.56892500
C	-4.33429400	-0.09768000	-2.32341100
H	-3.42684800	-2.04719400	-2.44505000
H	-3.97377400	2.06222600	0.28481400
H	-5.08064500	1.88981500	-1.93859300
H	-4.81264900	-0.18749900	-3.29714200
N	-2.47376300	0.19788000	1.43861900
H	-2.09604600	-0.70483500	1.72042500
O	-2.15395000	-2.01589800	-0.04931100
C	-1.97083300	-3.20834700	-0.78185400
H	-1.32183400	-3.84718300	-0.17222400
H	-2.92967800	-3.72454600	-0.94301800
H	-1.47449800	-3.00818900	-1.74289000
C	0.89048900	-1.29750500	2.40761500
C	1.08135100	-0.35391500	3.42388000
C	-0.17898900	-2.19577300	2.52617500
C	0.23492100	-0.31307300	4.52627100
H	1.91627900	0.34253300	3.35794600
C	-1.02938300	-2.15136200	3.62385400
H	-0.36436700	-2.91177900	1.72609000
C	-0.82352200	-1.21045400	4.63060100
H	0.40372400	0.42640200	5.30794400
H	-1.86052200	-2.85178300	3.69096500
H	-1.48546400	-1.17943000	5.49447400

C	3.51067100	-1.28729600	-2.59064500
C	3.66608500	-2.37511300	-3.45405900
C	3.74279200	0.00118900	-3.08692800
C	4.03567600	-2.18028400	-4.77946600
H	3.50052600	-3.38480800	-3.07989200
C	4.10453900	0.19716500	-4.41150600
H	3.58960500	0.86146700	-2.43377900
C	4.25271000	-0.89470300	-5.26333600
H	4.15193500	-3.03881400	-5.43883900
H	4.26244300	1.20719400	-4.78614200
H	4.53529600	-0.74191400	-6.30356700
H	1.51812800	3.89921300	0.24381600
C	-1.75790500	2.07540700	2.91129000
H	-0.86910000	2.68882100	3.10998700
H	-1.88643000	1.39162900	3.76550100
C	-2.98223100	2.93955500	2.75769600
C	-4.23215300	2.49545700	3.18819200
C	-2.89588100	4.16691900	2.09693900
C	-5.37283300	3.26234100	2.96932700
H	-4.30620000	1.52532300	3.68031500
C	-4.03225000	4.93784900	1.87855000
H	-1.92137700	4.51332200	1.74600100
C	-5.27575400	4.48639100	2.31502400
H	-6.34198500	2.89997700	3.30946800
H	-3.94832400	5.89561800	1.36641700
H	-6.16701200	5.08794400	2.14320900
H	-0.83767100	1.08185600	-1.12002700
O	-1.38872100	1.56116800	-2.50308500
H	-1.02781600	0.78557100	-2.98818600
H	-2.35038800	1.40470600	-2.47070000

B5: 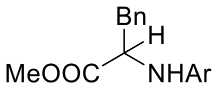

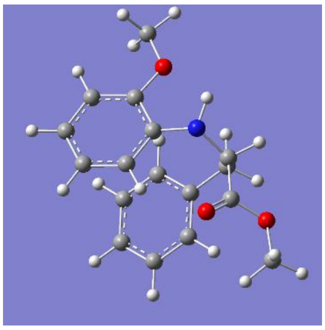

C	-6.87074000	0.18687700	1.00479400
C	-5.62096600	0.95134200	0.60267300

O	-5.50018500	1.75662400	-0.28394600
O	-4.59406500	0.57460400	1.39457300
C	-3.35908700	1.22806900	1.12285200
H	-3.03325800	1.02783300	0.09659100
H	-3.46033800	2.31244800	1.25006400
C	-11.12540800	2.17550000	0.25174100
C	-10.18834100	1.15127100	0.31961300
C	-8.83478800	1.38802000	0.00890000
C	-8.45267700	2.67531600	-0.35713500
C	-9.38463500	3.70812000	-0.40088200
C	-10.71752200	3.45966000	-0.10602200
H	-12.17021600	1.98726300	0.48768900
H	-7.40934200	2.84705900	-0.60864100
H	-9.06313700	4.70840300	-0.68468400
H	-11.45401400	4.25972300	-0.15591100
N	-7.93695600	0.29538700	0.02235800
H	-8.47684900	-0.56357800	0.04520900
O	-10.47850800	-0.13206000	0.70298600
C	-11.80796600	-0.42348600	1.04657700
H	-11.83478000	-1.48129500	1.32183400
H	-12.14321000	0.18093400	1.90435400
H	-12.49368900	-0.25235600	0.20321300
H	-2.64007900	0.82507000	1.83890600
C	-7.26739500	0.44684700	2.47996400
H	-6.46114300	0.04730800	3.11183300
H	-8.15845000	-0.16969300	2.66846000
C	-7.55298600	1.87515400	2.86309600
C	-8.85198900	2.28627600	3.16432400
C	-6.52567900	2.81922800	2.93769900
C	-9.12538200	3.60607300	3.50243300
H	-9.66504100	1.56015600	3.11008700
C	-6.79449000	4.14208600	3.26959300
H	-5.49798900	2.51011600	2.74331000
C	-8.09713900	4.54168100	3.54858700
H	-10.15007700	3.90600400	3.71704000
H	-5.98024800	4.86370200	3.31619300
H	-8.30966200	5.57810700	3.80557400
H	-6.55442000	-0.86755100	0.97218600

A11: 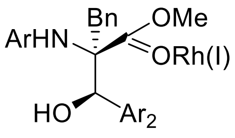

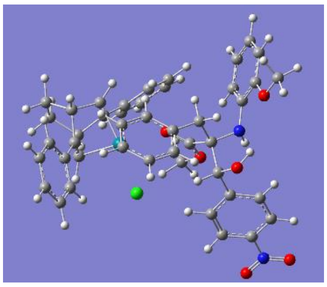

C	-3.96882200	0.18308000	4.09009100
C	-4.77575700	-0.78464400	5.02331900
N	-3.74661800	-1.46610700	5.83852900
C	-5.54389400	-1.82567400	4.17941200
C	-4.10151300	-2.66541900	6.52323700
C	-3.73953300	-3.90515500	5.95875500
C	-4.04949600	-5.08728400	6.63013400
C	-4.69598100	-5.04329500	7.86350200
C	-5.04379500	-3.82781900	8.43472800
C	-4.75165300	-2.64780200	7.75458900
C	-3.15997900	1.25678600	4.79384900
C	-1.82703000	1.02526500	5.14310700
C	-1.08055800	2.00234100	5.78687500
C	-1.68216200	3.22181700	6.06418000
C	-2.99725500	3.49355400	5.70290800
C	-3.72849000	2.50666100	5.06082600
C	-5.75781200	-0.05490200	5.93913700
O	-6.96674400	-0.22401600	5.91270400
O	-5.14849300	0.71093500	6.82675500
N	-0.90228000	4.25716100	6.74848200
O	-1.46203300	5.31192000	7.00453200
O	0.25966200	4.00173600	7.02644200
H	-3.77885800	-6.04971300	6.20349500
H	-4.92031800	-5.97585600	8.37832400
H	-5.53072500	-3.79503700	9.40822500
H	-1.36151600	0.07631900	4.88688200
H	-0.04426100	1.84024000	6.06893000
H	-3.42393900	4.46913800	5.91739300
H	-4.76415600	2.69872400	4.77169500
C	-5.96681800	1.51710100	7.68480000
H	-5.26646700	2.10066200	8.28524700
H	-6.59593900	2.17470100	7.07125100
H	-6.59206200	0.87701000	8.31879200
O	-3.09000600	-3.85871200	4.76987500
C	-2.69101800	-5.07735300	4.18843800
H	-3.55627400	-5.71339600	3.95012700
H	-2.17019300	-4.81935900	3.26314200

H	-2.00608200	-5.63254300	4.84618700
H	-5.02465300	-1.68256000	8.18768200
C	-6.51498900	-1.30986700	3.14682400
C	-6.07778900	-0.76975900	1.93357400
C	-7.89064300	-1.43682300	3.35431800
C	-6.99051700	-0.31275900	0.98679900
H	-5.00780300	-0.71881900	1.72911700
C	-8.80319500	-0.99520100	2.40367200
H	-8.24591800	-1.89643300	4.27761600
C	-8.35752200	-0.41408600	1.22097300
H	-6.62618200	0.11708200	0.05490600
H	-9.87282600	-1.10328400	2.58417700
H	-9.07642300	-0.05315500	0.48628800
Rh	-8.81334800	0.95169600	5.45170400
C	-12.38739400	1.40493900	7.04324400
C	-10.88737200	1.75447700	7.02197500
C	-11.40784100	0.06245200	5.14807500
C	-12.70074100	0.40011500	5.91697000
H	-12.63929000	0.98757700	8.02921900
H	-12.97344400	2.32646100	6.92659300
H	-13.12809100	-0.52719100	6.32207500
H	-13.43780700	0.81252200	5.21254900
C	-10.04923800	0.48850100	7.14772800
C	-10.32022900	-0.40867300	6.08869900
H	-10.05108700	-1.46470400	6.13329400
H	-11.58776400	-0.68423200	4.36756100
H	-10.63262800	2.50102000	7.78127700
C	-10.77444000	1.33433200	4.59775700
C	-10.45626100	2.23289000	5.64817200
H	-10.24751300	3.28688500	5.46417600
Cl	-7.27814300	2.55719300	4.38705500
C	-10.89441200	1.71358600	3.17758600
C	-11.73434500	0.99673600	2.31441400
C	-10.20308800	2.81889200	2.65935800
C	-11.87239300	1.36222600	0.97949800
H	-12.30854800	0.14824400	2.68361600
C	-10.33913200	3.18016800	1.32738200
H	-9.50708100	3.36398900	3.29404200
C	-11.17219400	2.45399400	0.47897700
H	-12.53463600	0.78996400	0.33158800
H	-9.77878100	4.03200800	0.94635100
H	-11.27455800	2.73911200	-0.56684100
C	-9.34274600	0.11307800	8.38684700
C	-9.46885300	0.87479300	9.55525500

C	-8.52135100	-1.02613100	8.43399500
C	-8.79942300	0.52065200	10.72273800
H	-10.10823500	1.75567000	9.56631100
C	-7.85666600	-1.38029300	9.59875500
H	-8.36886700	-1.62275500	7.53562100
C	-7.98859600	-0.60742300	10.75155100
H	-8.91810800	1.13204300	11.61568400
H	-7.22727000	-2.26996700	9.60206500
H	-7.46311000	-0.88499000	11.66349300
H	-6.08677400	-2.47442900	4.88058200
H	-3.36299200	-0.79208200	6.49867100
H	-4.78865300	-2.45200500	3.69224500
H	-4.69661600	0.68165900	3.43230400
O	-3.14719100	-0.62242000	3.28429500
H	-2.83243000	-1.34126600	3.86638900

4:

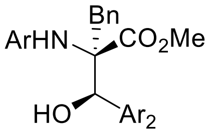

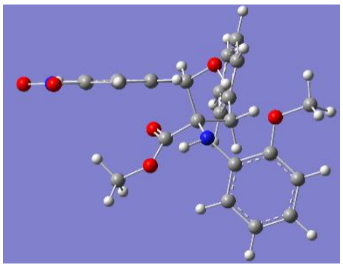

C	-3.95109500	0.03702300	4.07715700
C	-4.81115300	-0.73758900	5.13070800
N	-3.83705200	-1.42715700	5.99218300
C	-5.73248100	-1.76854500	4.43374300
C	-4.12251500	-2.69776400	6.56846100
C	-3.68285500	-3.86117800	5.90683200
C	-3.89057500	-5.11139400	6.48291400
C	-4.52091100	-5.21074200	7.72185000
C	-4.94751600	-4.07233400	8.38905500
C	-4.74876500	-2.82452600	7.80339600
C	-3.16647700	1.16320600	4.72081900
C	-1.85888500	0.96006700	5.16738600
C	-1.16007200	1.97377400	5.80616500
C	-1.78207700	3.20182800	5.98518400
C	-3.07227200	3.44550000	5.53167600
C	-3.75698800	2.42016700	4.89730200
C	-5.66498000	0.28296300	5.87717900

O	-6.53365600	0.94068100	5.35662100
O	-5.31181800	0.42788900	7.16686100
N	-1.05377500	4.27783500	6.66510400
O	-1.62713400	5.34704700	6.80660300
O	0.07934300	4.03960100	7.05307500
H	-3.55481500	-6.01409200	5.97893400
H	-4.67109100	-6.19462700	8.16265200
H	-5.43540600	-4.15029100	9.35832700
H	-1.38425600	-0.00296800	4.99705500
H	-0.14469700	1.83184500	6.16484400
H	-3.51321200	4.42827500	5.67216700
H	-4.76889800	2.59241000	4.53147300
C	-5.98694900	1.48066000	7.85493800
H	-5.59370700	1.47465400	8.87284300
H	-5.77912000	2.44025600	7.36810800
H	-7.06788000	1.30938500	7.85565300
O	-3.06776700	-3.66923400	4.70850900
C	-2.60542300	-4.80099300	4.00771900
H	-3.42857200	-5.48840400	3.76527600
H	-2.16663300	-4.42612600	3.07992900
H	-1.83870300	-5.34237800	4.58070100
H	-5.07997500	-1.91518100	8.30417100
C	-6.60289400	-1.28635400	3.30438100
C	-6.09177100	-1.18839300	2.00701900
C	-7.95011900	-0.99203500	3.51488100
C	-6.90634200	-0.79081100	0.95221900
H	-5.04045800	-1.42318100	1.83278000
C	-8.76757100	-0.59912900	2.46263400
H	-8.35873300	-1.06679300	4.52270500
C	-8.24705700	-0.49621900	1.17665000
H	-6.49098600	-0.71710000	-0.05176200
H	-9.81651500	-0.37327200	2.64809500
H	-8.88617800	-0.19070700	0.34975400
H	-6.36033700	-2.22686000	5.21237400
H	-3.52781200	-0.78432300	6.71599100
H	-5.07495900	-2.56034100	4.05574200
H	-4.63884900	0.47323500	3.33779800
O	-3.13285600	-0.88197800	3.41419900
H	-2.89773400	-1.56042700	4.08029800

TS-8: 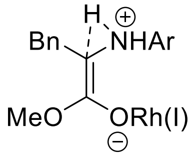

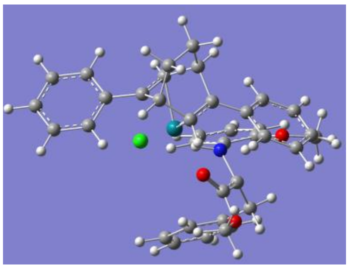

C	-2.21381500	0.86182100	-0.60175200
C	-1.21067200	1.13481300	-1.56150000
O	0.01162100	0.90113000	-1.50189500
O	-1.68923900	1.78618000	-2.64612200
C	-0.71683400	2.24377400	-3.56857900
H	-0.01371100	2.93487000	-3.08552500
H	-0.15076900	1.40934000	-3.99761900
Rh	1.62176000	0.09320800	-0.31953700
C	5.12441300	1.63806600	0.58254100
C	4.20462100	0.97580200	-0.45999300
C	2.91999100	1.29391100	1.75828200
C	4.34352900	1.86748200	1.89044200
H	5.99295700	0.98513600	0.74865300
H	5.50868000	2.58485500	0.17932600
H	4.84714800	1.38754700	2.74052200
H	4.27183600	2.93849100	2.12641900
C	3.67649100	-0.34722900	0.09573700
C	2.95518400	-0.15146000	1.29413500
H	2.68549100	-0.97412300	1.95660800
H	2.36072000	1.40814800	2.69295600
H	4.72218800	0.83300900	-1.41505900
C	2.20147000	1.94489300	0.57656800
C	2.91194800	1.74874800	-0.62802000
H	2.69297500	2.29775000	-1.54426800
Cl	0.62032000	-2.14615900	-0.33872100
C	-4.45997600	-0.63920100	2.98216200
C	-3.52443200	0.02827800	2.19769700
C	-2.64648200	-0.70370200	1.38417600
C	-2.67689300	-2.09307000	1.41132000
C	-3.59232600	-2.75929800	2.21975700
C	-4.48955800	-2.03265500	2.99083600
H	-5.15494900	-0.07820300	3.60273100
H	-1.98130000	-2.64044500	0.77581900
H	-3.61406500	-3.84669900	2.22415900
H	-5.22216700	-2.54511000	3.61178900
N	-1.70304900	-0.03275300	0.56006800
H	-1.70043100	1.19001100	0.57501200

H	-0.90710800	-0.62700500	0.27722700
O	-3.38383300	1.38175200	2.14993600
C	-4.42492100	2.16917800	2.66923100
H	-4.18252200	3.20676600	2.41862300
H	-5.39098600	1.90188900	2.21186400
H	-4.50638600	2.07226300	3.76236100
C	1.05990100	2.86294700	0.74024500
C	0.80397500	3.88323200	-0.18473100
C	0.15689300	2.69176900	1.79880700
C	-0.32138900	4.68976900	-0.06769400
H	1.50247900	4.05181500	-1.00375300
C	-0.96098300	3.50736600	1.92389600
H	0.30955000	1.88416500	2.51492000
C	-1.21020700	4.50648900	0.98729800
H	-0.49988000	5.47264600	-0.80353900
H	-1.64408700	3.34783700	2.75580000
H	-2.09022400	5.14117100	1.07982400
C	4.19371700	-1.64314200	-0.37680300
C	4.47278600	-2.67809100	0.52075500
C	4.41115200	-1.86473100	-1.74205600
C	4.95188900	-3.90088100	0.06788500
H	4.32064400	-2.51827100	1.58749200
C	4.88639900	-3.08686900	-2.19544000
H	4.16640000	-1.07951400	-2.45805600
C	5.16004300	-4.11002800	-1.29136200
H	5.16363000	-4.69463400	0.78250100
H	5.03616800	-3.24601100	-3.26214700
H	5.53315100	-5.06888100	-1.64748800
H	-1.27302200	2.76266100	-4.35315200
C	-3.66071100	0.72985700	-0.95891800
H	-3.83588700	1.39949800	-1.81194600
H	-4.30575100	1.10553900	-0.15084800
C	-4.07535200	-0.68336000	-1.30821200
C	-5.29579200	-1.18861400	-0.86050300
C	-3.23679800	-1.51756300	-2.04934000
C	-5.67732900	-2.49405700	-1.14987300
H	-5.93961800	-0.55216300	-0.24949400
C	-3.61172500	-2.82492700	-2.33833400
H	-2.26135500	-1.15437700	-2.37718800
C	-4.83455100	-3.31750800	-1.89061100
H	-6.63028900	-2.87450600	-0.78370300
H	-2.93827100	-3.46450700	-2.90681800
H	-5.12493900	-4.34381000	-2.11072000

Biological activity test:

PTP1B inhibitory assay

Recombinant human PTP1B catalytic domain was expressed and purified according to procedures described previously. In a typical 100 μL assay, mixture containing 50mM MOPS(3-[N-morpholino]propane-sulfonic acid), pH 6.5, 2mM pNPP and recombinant enzymes, PTP1B activities were continuously monitored on a VERSAmax microplate reader(Molecular Devices) at 405nm for 2min at 30 ℃ and the initial rate of the hydrolysis was determined using the early linear region of the enzymatic reaction kinetic curve. For the 50% inhibition concentration (IC_{50}) calculation, inhibition assays were performed with 30 nM recombinant enzyme, 2 mM pNPP in 50 mM MOPs at pH 6.5, and the inhibitors diluted around the estimated IC_{50} values. IC_{50} was calculated from the nonlinear curve fitting of percent inhibition (inhibition (%)) versus inhibitor concentration [I] using Graphpad Prisim 5.0 according to the formula: % Inhibition = $100/(1+[IC_{50}/[I]]k)$, where k is the Hill coefficient.

To study the inhibition selectivity on other PTP family members, human TCPTP, SHP1and LAR were prepared and assays were performed according to procedures described previously.
